# Parent-of-origin-specific allelic expression in the human placenta is limited to established imprinted loci and it is stably maintained across pregnancy

**DOI:** 10.1186/s13148-019-0692-3

**Published:** 2019-06-26

**Authors:** Diana Pilvar, Mario Reiman, Arno Pilvar, Maris Laan

**Affiliations:** 1 Institute of Biomedicine and Translational Medicine, University of Tartu, Ravila Str, 19 50411 Tartu, Estonia; 2 Veeuss OÜ, Jaama tn 185-49, 50705 Tartu, Tartu Estonia

**Keywords:** Human placenta, RNA-Seq, Parental-placental trios/duos, Imprinting, Biased parental allelic expression, Gestational dynamics, Pregnancy complications

## Abstract

**Background:**

Genomic imprinting, mediated by parent-of-origin-specific epigenetic silencing, adjusts the gene expression dosage in mammals. We aimed to clarify parental allelic expression in the human placenta for 396 claimed candidate imprinted genes and to assess the evidence for the proposed enrichment of imprinted expression in the placenta. The study utilized RNA-Seq-based transcriptome and genotyping data from 54 parental-placental samples representing the three trimesters of gestation, and term cases of preeclampsia, gestational diabetes, and fetal growth disturbances.

**Results:**

Almost half of the targeted genes (*n* = 179; 45%) were either not transcribed or showed limited expression in the human placenta. After filtering for the presence of common exonic SNPs, adequacy of sequencing reads and informative families, 91 genes were retained (43 loci form Geneimprint database; 48 recently proposed genes). Only 11/91 genes (12.1%) showed confident signals of imprinting (binomial test, Bonferroni corrected *P* < 0.05; > 90% transcripts originating from one parental allele). The confirmed imprinted genes exhibit enriched placental expression (*PHLDA2*, *H19*, *IGF2*, *MEST*, *ZFAT*, *PLAGL1*, *AIM1*) or are transcribed additionally only in the adrenal gland (*MEG3*, *RTL1*, *PEG10*, *DLK1*). Parental monoallelic expression showed extreme stability across gestation and in term pregnancy complications. A distinct group of additional 14 genes exhibited a statistically significant bias in parental allelic proportions defined as having 65–90% of reads from one parental allele (e.g., *KLHDC10*, *NLRP2*, *RHOBTB3*, *DNMT1*). Molecular mechanisms behind biased parental expression are still to be clarified. However, 66 of 91 (72.5%) analyzed candidate imprinted genes showed no signals of deviation from biallelic expression.

**Conclusions:**

As placental tissue is not included in The Genotype-Tissue Expression (GTEx) project, the study contributed to fill the gap in the knowledge concerning parental allelic expression. A catalog of parental allelic proportions and gene expression of analyzed loci across human gestation and in term pregnancy complications is provided as additional files. The study outcome suggested that true imprinting in the human placenta is restricted to well-characterized loci. High expression of imprinted genes during mid-pregnancy supports their critical role in developmental programming. Consistent with the data on other GTEx tissues, the number of human imprinted genes appears to be overestimated.

**Electronic supplementary material:**

The online version of this article (10.1186/s13148-019-0692-3) contains supplementary material, which is available to authorized users.

## Introduction

Genomic imprinting is a unique feature implicated in fine-tuning the dosage of gene expression in mammals. It is defined as an exclusive expression of either paternally or maternally derived allele of a gene, while the other allele is silenced via epigenetic reprogramming of germ cells *in utero* [1–3]. The majority of imprinted loci are localized within gene clusters and the expression of either maternal or paternal set of genes is tightly coordinated at the genomic level. For some specific tissues, such as the placenta, additional ungrouped “singleton” imprinted genes have been reported [4]. Failure in programming genomic imprints may cause severe developmental disorders and fetal growth disturbances [3, 5].

Analyses of human imprinted genes have been facilitated by the advanced ‘omics’ toolsets [6–8]. Two recent RNA sequencing (RNA-Seq) based analyses utilizing the Genotype-Tissue Expression (GTEx) dataset across diverse sets of human post-mortem tissues from 178 individuals reported only 12 and 17 novel imprinting candidate genes, respectively [7, 8]. The overall number of identified imprinted human genes was lower than initially thought, only 72 (42 high-confident, 30 suggestive) genes and 93, respectively. The data also showed a widespread tissue specificity of imprinting and/or variable maintenance of imprinted status among loci across tissues [7, 8].

Although the underlying reasons of imprinting and its ‘rationale’ in genome function remain debated, it is generally accepted that this phenomenon arose in parallel with the evolution of the mammalian placenta [1, 9]. Consistent with the evolutionary context, well-known imprinted genes are critical in regulating human placental function and fetal development, including tissue-specific imprinted microRNA clusters [9–11]. Recent studies applying genome-wide allelic DNA methylation analyses of human placentas have suggested a potential organ-specific enrichment of imprinted genes, highly variable imprinting, and possible polymorphic silencing of preferably maternal gene alleles [6, 12–14]. Whereas DNA methylation-based studies are valuable tools to identify loci exhibiting either maternal or paternal allele-specific methylation as indicative markers to imprinting, RNA-Seq enables to directly assess transcripts exhibiting parent-of-origin-specific allelic expression. As placental tissues are not included in GTEx, the analysis of parental transcripts in the human placenta has been lagging behind. So far, only two small-scale RNA-Seq studies have been published profiling of parent-of-origin expression and reporting novel imprinting candidate genes in either human term placentas (*n* = 10, [15]) or early pregnancy chorionic villus samples (*n* = 21; [14]). However, some of these claims were based on single samples, the applied criteria to define imprinting varied between the studies and the majority of novel reported candidate imprinted loci have been not identified as imprinted genes in other tissues. Thus, there are remaining uncertainties and contradictions among the claims regarding the landscape of imprinting in the human placenta and there has been a lack of transcriptome-based studies analyzing adequate numbers of parental-placental samples.

The current study aimed to clarify the parental allelic expression status in the human placenta for nearly 400 claimed candidate imprinted genes and to confirm (or reject) the evidence for the suggested enrichment of imprinted genes in placental transcriptome compared to other tissues. The study utilized RNA-Seq-based placental transcriptome data and the corresponding genotyping data from 54 parental-placental samples collected from all three trimesters of gestation, as well as term cases of preeclampsia, gestational diabetes, and fetal growth disturbances. Among 91 tested genes with adequate placental expression and available sequencing data from at least 3 informative family trios/duos, only 11 genes showed high-confidence imprinting signals, i.e., nearly monoallelic parent-of-origin determined allelic expression. Additional 14 genes exhibited transcript profiles consistent with biased proportions of parental alleles. The majority, 66 of 91 (72.5%) analyzed candidate imprinted genes were convincingly detected to be expressed in the human placenta in a biallelic manner.

## Methods

### Datasets of parental-placental trio or maternal-placental duo samples

The study exploited previously published 54 placental RNA-Seq datasets [16–18] and the corresponding genome-wide genotyping data of placental and respective parental blood samples [19, 20]. The dataset was comprised of 38 parental (mother, father)-placental trios and 16 maternal-placental duos (Table 1). Placental and parental blood samples of singleton term pregnancy cases (delivery ≥ 37th gestational week) had been collected at the delivery room during the REPROMETA study (Additional file 1: Supplementary Methods). The recruited term pregnancy groups represented cases of uncomplicated gestation (normal third trimester), maternal preeclampsia (PE), gestational diabetes (GD), delivery of a small- (SGA, < 10th birth weight centile) or large-for-gestational-age (LGA, > 90th centile) newborn according to national guidelines [21]. The dataset analyzed in the current study included 38 term pregnancy trios and 2 duos (paternal DNA samples unavailable), delivered at median gestational age (g.a.) 275.5 [260–291] days (Additional file 2: Table S1). Each group (normal third trimester; PE, GD, SGA, and LGA) was represented by eight cases that were matched for gestational age. Additional 14 maternal-placental duos represented 8 electively surgically terminated pregnancies during the first trimester (60 [51–81] gestational days (g.d.)) and 6 medically induced abortions during the second trimester due to maternal health indications (138 [126–167] g.d.) (Additional file 3: Table S2). Gross chromosomal abnormalities in the analyzed placentas had been excluded by placental karyotyping. For the second trimester terminated pregnancies, fetal anomalies were excluded by the pathology specialist assessment.

**Table 1.** Analyzed pregnancy cases and study material

	Terminated pregnancy^a^		Term pregnancy				
	First trimester	Second trimester	Normal	SGA	LGA	PE	GD
Pregnancy-specific variables							
Gestational age (days)	60.0 (51–81)	138.0 (126–167)	284.0 (260–291)	268.5 (264–289)	280.5 (275–288)	266.0 (260–271)	275.5 (268–284)
Offspring sex, F/M (*n*)	4/4	3/3	3/5	5/3	4/4	4/4	5/3
C-sect/vaginal delivery (*n*)	–	–	5/3	6/2	3/5	2/6	3/5
Maternal age (years)	25.5 (18–33)	23.0 (15–36)	33.0 (18–37)	24.5 (20–32)	30.0 (23–39)	26.5 (19–39)	32.5 (22–36)
Paternal age (years)	n.a.	n.a.	34.0 (22–38)	26.0 (23–39)	35.5 (23–50)	32 (21–46)	34 (22–43)
Study material—placental and parental blood samples							
Samples	Chorion	Full-thickness samples from the middle region of placenta					
Mat/Pat/Pl (*n*)	–	–	8	7	7	8	8
Mat/Pl (*n*)	8	6	–	1	1	–	–

Data is presented as median (range), if not indicated otherwise. Detailed information on the analyzed pregnancy cases is presented in Additional file 2: Table S1 and Additional file 3: Table S2

^a^Chorionic villi from first trimester placentas were sampled after elective surgical termination of pregnancy. Samples of second trimester placentas were derived from cases of medically induced abortion due to maternal health indications

*C-sect, Cesarean delivery; GD*, gestational diabetes; *LGA*, delivery of a large-for-gestational-age newborn; *normal*, term pregnancy without any maternal or fetal complications; *PE*, preeclampsia; *SGA*, delivery of a small-for-gestational-age newborn; *F*, female; *M*, male; *n*, number; *n.a.*, not available; *Mat*, maternal blood sample; *Pat*, maternal blood sample; *Pl*, placental sample

### Placental sampling, RNA sequencing, and genotyping

A detailed description of placental sampling, RNA extraction, sequencing procedures, and bioinformatic processing has been described previously [16–18] and is provided in Additional file 1: Supplementary Methods. Briefly, for term and second-trimester pregnancy placentas, the sampling was performed through all layers of the middle region of the placenta. Samples of the first trimester placentas were obtained immediately after surgical termination of pregnancy. The maternal tissue was removed under a stereomicroscope (Discovery V8, Zeiss) and chorionic villi containing both cyto- and syncytiotrophoblast cells were sampled. For DNA studies, the placental or chorionic villus samples were placed immediately into dry cryovial, and for the RNA studies into RNAlater solution (Thermo Fisher Scientific, Waltham, MA, USA). The samples were kept at − 80 °C until DNA/RNA isolation.

Total placental RNA was extracted using TRIzol reagent (Invitrogen, Life Technologies) and purified with RNeasy MinElute columns (Qiagen, Netherlands). rRNA depletion, preparation of RNA-Seq sequencing libraries, sequencing of transcriptomes (Illumina HiSeq2000) and basic bioinformatic processing of the raw sequencing data (QC, read alignment and transcript and gene expression estimation) were performed according to the established pipeline at the Sequencing Unit of Finnish Institute of Molecular Medicine (FIMM), University of Helsinki, Finland. Initial data analysis was conducted using the in-house RNA-Seq pipeline v2.4 (FIMM). Sequencing reads were filtered for the quality, the presence of the adaptor, rRNA, and mtDNA sequences, as well as homopolymer stretches using custom python scripts. Read alignment to human genome assembly (GRCh37.p7/hg19) was performed with TopHat version 2.0.3 [22] and read counts per gene were estimated using htseq-count [23], based on reference annotations from Ensembl v67 [24]. To compare expression among genes, transcript levels were additionally quantified as FPKM (fragments per kilobase per million), implemented in Cufflinks v 2.0.2 [25]. The complete dataset across 54 placental transcriptomes consisted of 2.28 billion paired-end reads (mean 42.3 million per sample; range 27.3–74.6 million) with an average alignment success rate of 82.6% (range 56.2–87.3%). Median estimate for the fraction of RNA originating from maternal cells was previously calculated to be 0.93% [16].

Placental and blood genomic DNA was genotyped using Illumina HumanOmniExpress-12-v1/24-v1 BeadChips (> 715,000 markers with median spacing 2.1 kb) [19, 20]. In the current study, we only analyzed exonic SNPs mapped in imprinted candidate genes and with minor allele frequency (MAF) > 10%. Genotype distributions of all analyzed SNPs were in Hardy-Weinberg equilibrium (*P* > 0.05).

### Formation and filtering of the candidate imprinted gene list

The list of human genes predicted to exhibit parent-of-origin determined allelic expression were retrieved from the Geneimprint database, the last access May 25, 2018 (*n* = 300) [26]. The list was supplemented with 96 recently reported novel candidate imprinted genes in the human placenta [6, 14, 15]. As polymorphic imprinted transcripts were not targeted in this study, the analyzed gene list did not include the respective proposed candidate loci [12, 13]. Total number of the genes entering the analysis pipeline was 396 (Additional file 4: Table S3).

To determine the parental origin of analyzed placental transcripts with high confidence, a stringent filtering pipeline, and data QC were applied (Additional file 4: Table S3). The first step included checking the gene annotations in the human genome assembly (GRCh37.p7/hg19) and assessing the sufficiency of placental gene expression using empirically assigned threshold (median normalized expression < 50 reads across all samples [16]. For the retained 207 genes, Ensembl Biomart tool [27] was implemented to identify common (1000 Genomes Project dataset: MAF > 10%) biallelic exonic SNPs within the available parental-placental genotyping dataset (dropout 9 genes). Custom scripts were developed to identify informative family trios/duos for each SNP to assess the parental origin of the expressed transcripts. Family trios/duos were defined as informative if the placenta had heterozygous genotype of the SNP and at least one of the parents had homozygous genotype of this variant (Additional file 5: Figure S1). Retained SNPs had to be informative for at least 3 family trios/duos (dropout 47 genes). Next, the maternal and paternal read counts at the selected marker SNP positions for each gene were called from the placental RNA-Seq dataset of the informative families (BAM files). Samtools mpileup command [28] was applied with the following parameters: -ABQ 0 (reference genome GRCh37.p7). Upon manual inspection of RNA-seq reads visualized using the IGV 3.0 software [29], SNPs located within alternative exons overlapping with introns of the main transcript and SNPs with < 3 median reads at the variant position across all informative placentas, were discarded (dropout 17 and 43 genes, respectively). The final analyzed dataset was comprised of 91 genes and 227 SNPs. It included 43 genes listed in the Geneimprint database and 48 genes derived from recent publications (9, 19, and 20 genes from ref. [6], ref [15], and ref. [14] respectively).

### Analysis of parental transcript ratios and gene imprinting status

For each gene, the proportions of maternal (Mat) and paternal (Pat) reads across all samples were calculated and the outcome was expressed as Mat/Pat reads ratio along with the estimated 95% confidence interval (CI). The observed parental transcript ratios were statistically tested under the assumption that both alleles are expressed at equal levels, using binomial test implemented in R. Statistical significance level was defined *P*<0.05 after application of Bonferroni correction for the number of conducted tests (one test per gene, total 91). A gene was defined as imprinted if at least 90% of the RNA-Seq reads were assigned to only one parental allele, i.e., close to monoallelic expression in the parent-of-origin-specific manner. Among the rest of the genes with statistically significant deviation from the expected maternal/paternal transcript ratio, loci with ≥ 65%, but < 90% reads originating from one parental allele were defined to exhibit biased parental allelic expression. A gene was considered biallelic when the proportions of parental reads did not differ significantly from the expected ratio (*P*_corr_ > 0.05) and/or the estimated proportions of both parental allelic reads fall within 35–65%.

### Validation of the parental origin of transcripts

Validation of the maternal allelic expression of *RTL1* was performed on three placental-parental trios informative for two SNP alleles using RT-PCR, cloning, and sequencing of the region. *DLK1* served as a reference of a paternally expressed gene and the *PAPPA2* (RNA-Seq: biallelic expression) and *RHOBTB3* (paternally biased expression) transcripts were cloned as positive controls for the capture of bi-parental expression, if present. cDNA was synthesized from 1 μg total placental RNA according to the manufacturer’s instructions (SuperScript III Reverse Transcriptase, Life Technologies). cDNA fragments were amplified by PCR from placental samples using PCR primers provided in Additional file 6: Table S4. To reach high-confidence conclusions about the transcribe allele of the *RTL1* gene, long-range PCR (2357 bp) was designed, incorporating simultaneously two marker SNPs (rs3825569, rs6575805). Purification, cloning, and sequencing of PCR products are detailed in Additional file 1: Supplementary Methods. RT-PCR, cloning, and sequencing experiments analyzed at least 10 clones per SNP. DNA sequences were visualized and analyzed using the Bioedit software [30].

## Results

### Half of the candidate imprinted genes have no or low expression in the human placenta

**Fig. 1:**
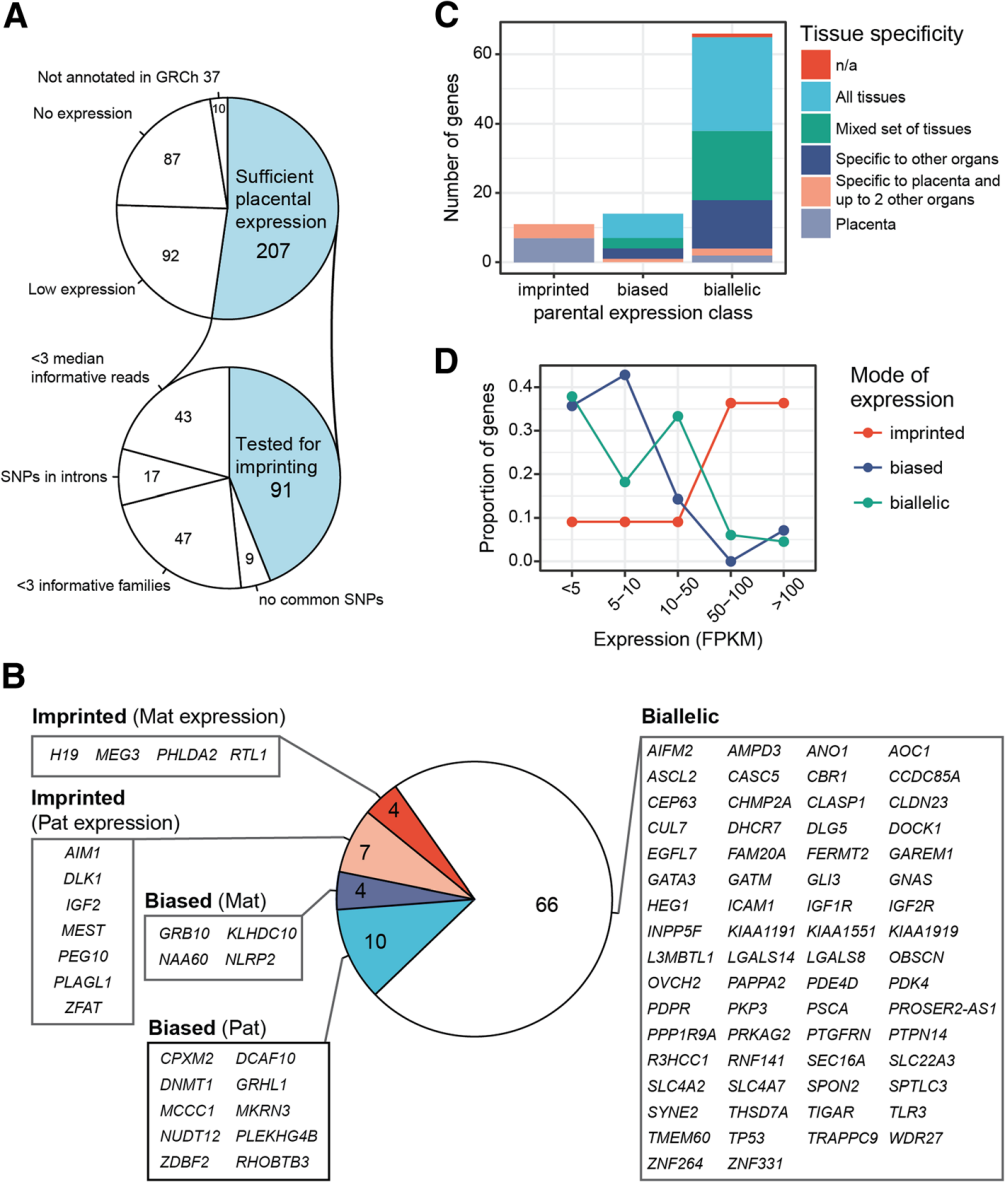
Placental expression profile of the candidate imprinted genes. **a** Filtering 396 candidate imprinted genes for the inclusion to the high-confidence analysis for the parental allelic expression (details provided in Additional file 4: Table S3). The list of 300 human genes predicted to exhibit parent-of-origin determined allelic expression were retrieved from the Geneimprint database [26]. The list was supplemented with 96 recently reported novel candidate imprinted genes in the human placenta [6, 14, 15]. **b** The analyzed geneset included 11 true imprinted genes with parent-of-origin-specific transcription, 14 genes with biased parental allelic expression, and 66 biallelic loci. **c** Expressional breadth across human tissues and **d** the abundance of placental transcripts of the analyzed genes stratified based on the parental allelic expression. Human tissue data was derived from the Protein Atlas database [31]. FPKM, fragments per kilobase of transcript per million mapped reads; Mat, maternal; n/a, not available; Pat, paternal.

The initial list of 396 candidate imprinted genes was assembled based on the Geneimprint database and recent reports on potential novel placental imprinted genes [6, 14, 15]. The analyzed RNA-Seq dataset of 54 placental samples covered a broad spectrum of pregnancy scenarios, including uncomplicated gestations across all three trimesters and adverse pregnancy outcomes at term (cases of PE, GD, SGA, LGA; Table 1; Additional file 2: Table S1; Additional file 3: Table S2). In total 189 genes (47.7%) were filtered out in the first step as they were not properly annotated (10 genes), had no (87) or limited (92) placental expression in our dataset (Fig. 1a; Additional file 4: Table S3). The retained 207 genes were further assessed for the presence of common genotyped SNPs in coding regions and their unambiguous exonic location, adequacy of read counts at the variant position and the availability of minimum three informative family trios/duos in our dataset to determine the parental origin of transcribed alleles (Additional file 5: Figure S1). The set of loci that passed all QC criteria for the analysis of the parental allelic expression comprised of 91 genes and 227 SNPs (Fig. 1a; Additional file 4: Table S3; Additional file 7: Table S5).

### Parental monoallelic expression is limited to well-known placental imprinted genes

**Fig. 2:**
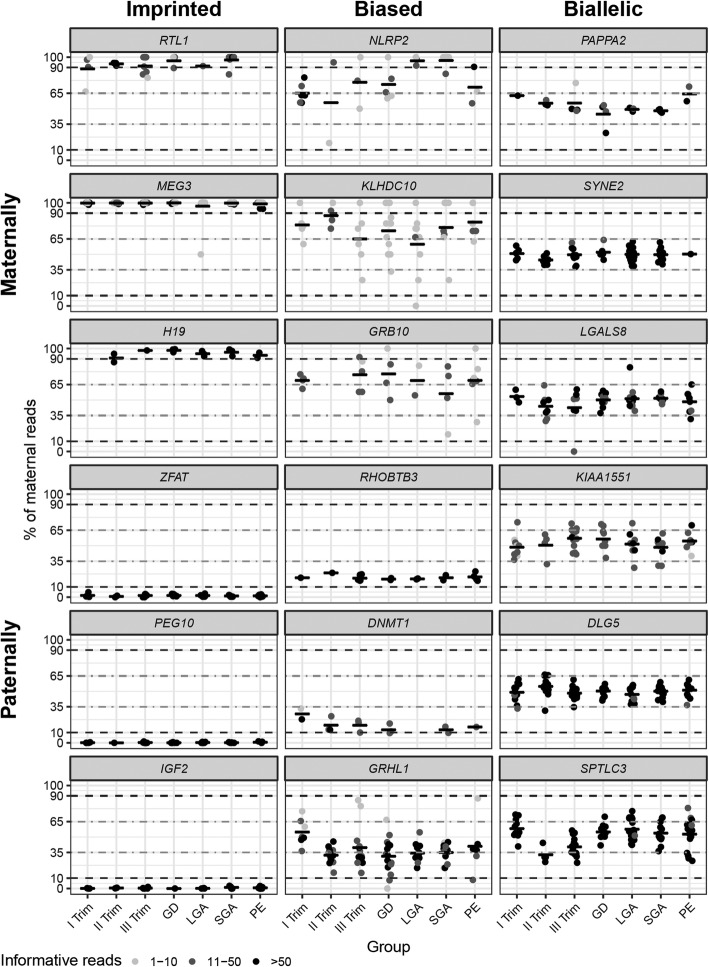
Examples of analyzed candidate imprinted genes stratified based on the proportions of transcribed parental alleles. A gene was confirmed as imprinted, when it was expressed in a high-confidence parent-of-origin-specific manner (binomial test, *P*_corr_ < 0.05; > 90% transcripts originating from one parental allele). Biased parental allelic expression was defined when a significant deviation from the equal proportions of transcribed parental alleles was observed, but it did not correspond to exclusive monoallelic transcription (binomial test, *P*_corr_ < 0.05; 65–90% of reads from one parental allele). A gene was confirmed as biallelically expressed when the proportions of parental reads did not differ significantly from the expected ratio (*P*_corr_ > 0.05) and/or the estimated proportions of both parental allelic reads fall within 35–65%. Detailed information on all analyzed genes is provided in Additional file 8: Table S6 and in the assembled gene-based catalog (Additional file 9: Figure S2), including data of parental allelic proportions and expression for all analyzed clinical subgroups. GD, gestational diabetes; LGA, large-for-gestational-age newborn; PE, preeclampsia; SGA, small-for-gestational-age newborn; Trim, trimester

Only 11 of 91 (12.1%) analyzed genes were expressed in the human placenta in an exclusive parent-of-origin manner and were classified as high confidence imprinted genes (binomial test, *P*_corr_ < 0.05; > 90% transcripts originating from one parental allele; Fig. 1b, Table 2, Additional file 7: Table S5; Additional file 8: Table S6). The median fraction of reads detected from the preferred parental allele was as high as 97.6% and for all confirmed imprinted genes the proportions of parental transcripts showed an extremely stable pattern across three trimesters of normal gestation and in all analyzed term pregnancy complications (Fig. 2; Additional file 9: Figure S2). Among paternally expressed genes, the most stringent level of imprinting was identified for *PEG10* and the least conservative for *AIM1* (99.8% and 93.7 % of paternal reads, respectively). Among maternal genes, the constraint for parental monoallelic expression was the highest for *MEG*3 (99.5% of maternal reads) and the lowest for *H19* (93.6%). Interestingly, there were more paternally than maternally expressed imprinted genes identified (Fig. 1b). Except for *RTL1*, the parental origin of transcripts was consistent with the literature data. Although previously reported to be paternally expressed in the mouse placenta [32], our RNAseq data and subsequent experimental validation showed that *RTL1* is a maternally expressed gene in the human placenta (Additional file 10: Table S7). All but one (*ZFAT*) of the high-confident imprinted genes expressed in the placenta are also imprinted in the mouse (Table 2).

**Table 2:** Genes with parent-of-origin driven allelic expression in the human placenta

Gene	FPKM Mean (SD)^a^	RNA-Seq reads			Binominal test corrected *P* value^b^	Locus class	Expressed allele		Imprinting in mouse (expressed allele)^d^
		Mat	Pat	Maternal read proportion (95%CI)			This study	Previous studies ^c^	
*MEG3*	104.0 (83.7)	5002	23	1 (0.99–1)	< 2.2 × 10^−300^	Imprinted	Mat	Mat	Imprinted (Mat)
*PHLDA2*	5.9 (4.1)	154	5	0.97 (0.94–0.99)	2.2 × 10^−37^	Imprinted	Mat	Mat	Imprinted (Mat)
*RTL1*	4.0 (3.0)	687	35	0.95 (0.94–0.96)	4.3 × 10^−156^	Imprinted	Mat^e^	Pat	Imprinted (Pat)
*H19*	498.0 (319.3)	20522	1393	0.94 (0.93–0.94)	4.7 × 10^−322^	Imprinted	Mat	Mat	Imprinted (Mat)
*PEG10*	102.1 (49.8)	13	6101	0 (0–0)	<2.2 × 10^−300^	Imprinted	Pat	Pat	Imprinted (Pat)
*IGF2*	84.5 (54.8)	52	6695	0.01 (0.01–0.01)	<2.2 × 10^−300^	Imprinted	Pat	Pat	Imprinted (Pat)
*MEST*	97.3 (52.3)	10	850	0.01 (0.01–0.02)	1.5 × 10^−234^	Imprinted	Pat	Pat	Imprinted (Pat)
*ZFAT*	101.8 (24.0)	138	9652	0.01 (0.01–0.02)	4.7 × 10^−322^	imprinted	Pat	Pat	Biallelic
*PLAGL1*	22.7 (9.5)	10	402	0.02 (0.01–0.04)	6.5 × 10^−103^	Imprinted	Pat	Pat	Imprinted (Pat)
*DLK1*	54.9 (41.7)	50	1467	0.03 (0.03–0.04)	<2.2 × 10^−300^	Imprinted	Pat	Pat	Imprinted (Pat)
*AIM1*	55.6 (17.8)	65	971	0.06 (0.05–0.08)	4.3 × 10^−206^	Imprinted	Pat	Pat	n.a.
*KLHDC10*	5.8 (1.4)	248	83	0.75 (0.71–0.79)	3.0 × 10^−18^	Biased	Mat	Mat	n.a.
*NLRP2*	14.9 (5.8)	697	282	0.71 (0.69–0.74)	3.3 × 10^−39^	Biased	Mat	Mat	n.a.
*GRB10*	9.9 (4.7)	302	146	0.67 (0.64–0.71)	1.3 × 10^−11^	Biased	Mat	Isoform dependent	Imprinted (isoform dependent)
*NAA60*	6.0 (1.3)	164	82	0.67 (0.61–0.72)	1.8 × 10^−5^	Biased	Mat	Mat	n.a.
*CPXM2*	9.1 (10.79)	72	588	0.11 (0.09–0.13)	1.36 × 10^−99^	Biased	Pat	Pat	n.a.
*MCCC1*	4.3 (1.0)	14	101	0.12 (0.08–0.18)	1.9 × 10^−15^	Biased	Pat	Pat	n.a.
*PLEKHG4B*	0.9 (0.7)	52	292	0.15 (0.12–0.19)	1.1 × 10^−39^	Biased	Pat	Pat	n.a.
*DCAF10*	4.2 (0.7)	15	78	0.16 (0.1–0.24)	1.9 × 10^−9^	Biased	Pat	Pat	n.a.
*DNMT1*	8.9 (4.7)	71	353	0.17 (0.14–0.2)	4.5 × 10^−44^	Biased	Pat	Pat	n.a.
*NUDT12*	2.1 (0.9)	5	24	0.17 (0.07–0.33)	5 × 10^−2^	Biased	Pat	Pat	n.a.
*RHOBTB3*	358.0 (130.8)	2264	10119	0.18 (0.18–0.19)	4.7 × 10^−322^	Biased	Pat	Pat	n.a.
*ZDBF2*	8.9 (2.4)	226	878	0.2 (0.18–0.23)	3.7 × 10^−89^	Biased	Pat	Pat	Imprinted (Pat)
*MKRN3*	3.5 (1.0)	44	102	0.3 (0.24–0.37)	1.7 × 10^−4^	Biased	Pat	Pat	Imprinted (Pat)
*GRHL1*	28.7 (7.0)	1825	3399	0.35 (0.34–0.36)	1.2 × 10^−104^	Biased	Pat	Pat	n.a.

Results of the binominal test, parental allelic proportions, and expressional information for the full analyzed dataset of 91 genes is provided in Additional file 7: Table S5 and Additional file 8: Table S6

^a^Gene expression level across all analyzed placental samples, including the three trimesters of uncomplicated pregnancy and four clinical subgroups of complicated pregnancies (preeclampsia, gestational diabetes, delivery of a small- or large-for gestational age newborn)

^b^The observed parental transcript ratios were statistically tested under the assumption that both alleles are expressed at equal levels, using binomial test implemented in R. Statistical significance level was defined *P* < 0.05 after application of Bonferroni correction for the number of conducted tests (*n* = 91)

^c^Sources of the previously reported parental allelic expression information are provided in Additional file 4: Table S3

^d^ Data from Geneimprint database (http://www.geneimprint.com/)

*FPKM*, fragments per kilobase of transcript per million mapped reads; *Mat*, maternal; *Pat*, paternal; *SD*, standard deviation; *n.a*., not available

^e^Maternal allelic expression was experimentally confirmed by RT-PCR, cloning, and sequencing (Additional file 10: Table S7)

**Fig. 3:**
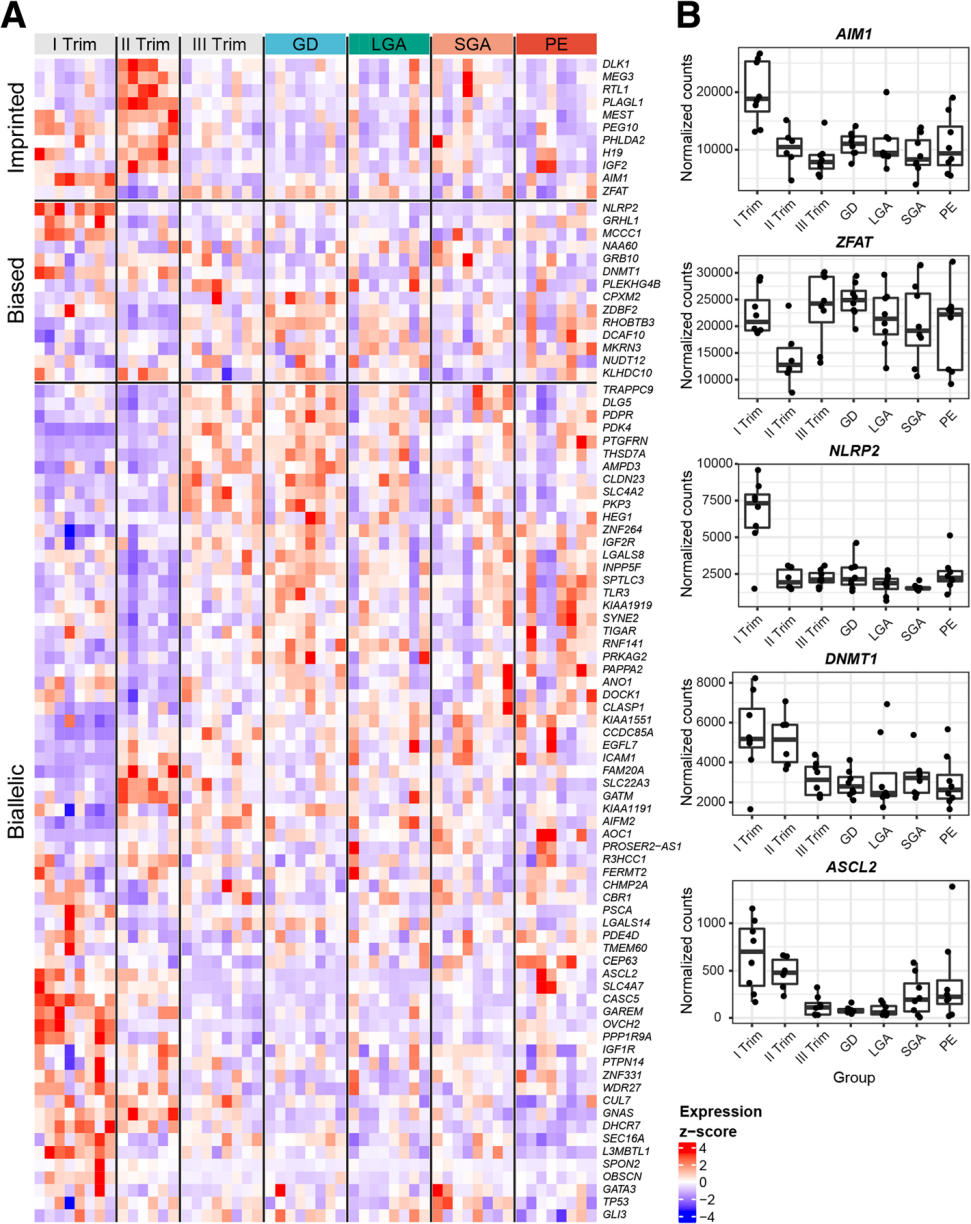
Expression of 91 analyzed genes across 54 placentas in normal gestation and term pregnancy complications. **a** Heatmap with hierarchical clustering of the analyzed genes was generated based on transformed read counts. Gene expression levels were subjected to variance stabilizing transformation in DESeq2 and standardized by subtracting the mean expression across all samples from its value for a given sample and then dividing by the standard deviation across all the samples. The scaled expression value is denoted as the row *Z*-score. Clustering of the genes (rows) within each of the three parental allelic expression class (imprinted, biased, biallelic) and samples (columns) within the clinical subgroups was based on Minkowski distance. **b** Expression dynamics of some examples of imprinted (*AIM1*, *ZFAT*), biased (*NLRP2, DNMT1*), and biallelic (*ASCL2*) genes expressed as normalized read counts. GD, gestational diabetes; LGA, large-for-gestational-age newborn; PE, preeclampsia; SGA, small-for-gestational-age newborn; Trim, trimester

The confirmed imprinted genes are either placenta-specific (*AIM1*, *H19*, *IGF2*, *MEST*, *PHLDA2*, *PLAGL1*, *ZFAT*) or additionally transcribed only in the adrenal gland (*DLK1*, *MEG3*, *PEG10*, *RTL1*) (Fig. 1c; Table 3). Most of them show high placental expression with the peak transcript levels during mid-gestation (Figs. 1d and 3, Additional file 9: Figure S2). The transcription of paternally expressed *AIM1* was specifically enhanced in early pregnancy, whereas *ZFAT* exhibited an unusual expression dynamics characterized by specifically reduced transcript levels during mid-gestation. None of the imprinted genes showed systematic expressional bias in the placentas from analyzed term cases of preeclampsia, gestational diabetes, and deliveries of SGA or LGA newborns.

**Table 3.** Expressional breadth and function of genes exhibiting either imprinting or biased parental allelic expression in the human placenta

Gene	Tissues with imprinted expression/all tissues with available expression data in GTEx [7]	Tissue expression	Function
Confirmed placental imprinted genes			
*MEG3* (ncRNA)	Imprinting in all 33 available tissues	Placenta and adrenal enriched	Tumor suppressor; angiogenesis inhibitor
*PHLDA2*	Limited transcription in other organs than placenta and biallelic expression in all these tissues	Placenta enriched	Trophoblast function
*RTL1*	Imprinting in adrenal, brain, pituitary tissues	Placenta and adrenal enriched	Maintenance of the fetal capillaries
*H19* (ncRNA)	Imprinting in 34/35 tissues	Placenta enriched	Tumor suppressor
*PEG10*	Imprinting in 26/30 tissues	Placenta and adrenal enriched	Cell proliferation, differentiation, apoptosis
*IGF2*	Imprinting in 25/33 tissues^a^	Placenta enriched	Fetal development and growth
*MEST*	Imprinting in 19/33 tissues	Placenta enriched	Invasion of extravillous trophoblast
*ZFAT*	Limited transcription in other organs than placenta and biallelic expression in all these tissues	Placenta enriched	Regulator of apoptosis and cell survival
*PLAGL1*	Imprinting in 31/34 tissues	Placenta enriched	Suppressor of cell growth
*DLK1*	Imprinting in 10/12 tissues	Placenta and adrenal enriched	Cell growth and differentiation
*AIM1*	n.a.	Placenta enhanced	Transporter mediating melanin synthesis
Genes with high-confidence biased parental allelic expression in the placenta			
*KLHDC10*	No reported imprinting signals in any tissues	All tissues	Oxidative stress-induced cell death
*NLRP2*	Non-parental monoallelic expression, but not consistent with imprinting	Mixed tissues; high in testis	Regulation of immune response, inflammation
*GRB10*	Imprinting in the brain^b^	All tissues	Interaction with insulin receptors and insulin-like growth-factor receptors
*NAA60*	No reported imprinting signals in any tissues	All tissues	Chromatin assembly, chromosome integrity
*CPXM2*	No reported imprinting signals in any tissues	Epididymis and smooth muscle enhanced	Carboxypeptidase enzyme
*MCCC1*	No reported imprinting signals in any tissues	All tissues	Carboxylation enzyme
*PLEKHG4B*	No reported imprinting signals in any tissues	Thyroid and pituitary enhanced	Guanine nucleotide exchange factor
*DCAF10*	No reported imprinting signals in any tissues	All tissues	Possibly ligase function
*DNMT1*	No reported imprinting signals in any tissues	All tissues	Maintenance of DNA methylation
*NUDT12*	No imprinting, but heterogeneous patterns of monoallelic expression	Mixed tissues	Regulation of nucleotides concentrations
*RHOBTB3*	No reported imprinting signals in any tissues	All tissues	Associated with Golgi, GTPase function
*ZDBF2*	Imprinting in 32/34 tissues	Mixed tissues	Zinc finger protein with unknown function
*MKRN3*	Imprinting in the brain, esophagus (mucosa)	Brain, placenta, testis enhanced	Inhibitor of GnRH secretion in childhood
*GRHL1*	No reported imprinting signals in any tissues	Esophagus and skin enhanced	Transcription factor critical in the development

For the majority of genes, the information on the expression in human tissues/organs was derived from Protein Atlas [31]. For RNA genes, the information on the tissue expression was derived from NCBI Gene [33]. The same database was applied to extract functional information on the analyzed genes. *n.d.*, not described

^a^Paternal allele expression in all tissues except brain with maternal allele expression

^b^Paternal expression in brain and maternal in the placenta

### Genes with biased parental allelic expression in the human placenta

Additional group of 14 candidate imprinted genes (15.4%) were detected with high confidence to exhibit biased parental allelic expression in the placenta (binomial test, *P*_corr_ < 0.05; 65–90% of reads from one parental allele; Table 2, Figs. 1 and 2; Additional file 7: Table S5; Additional file 8: Table S6). The proportions of parental reads of most biased genes showed substantial variability among the analyzed placentas. More loci were identified with paternal (10 genes) compared to maternally biased expression (4 genes). In addition, preferential transcription of maternally biased genes was less pronounced compared to the paternally biased allele genes (median 69.3% vs. 83.0% of reads from the preferred parental allele, respectively). Among genes with preferred maternal allele expression, the most skewed transcript ratio was identified for *KLHDC10* (74.9 % of maternal reads), whereas the highest paternal read counts were detected for *CPXM2* gene (89.1%). Despite that these candidate imprinted genes showed only biased (not exclusively monoallelic) parental allelic expression, the preferentially transcribed allele for all 14 genes was concordant with the data in previous reports (Table 2).

Notably, none of the genes with biased parental allelic expression is placenta-specific (Fig. 1c, Table 3). These genes (except for *MKRN3*) are either transcribed in a broad range of tissues or preferentially in some other organ, and their placental expression level tends to be modest with the exception of *RHOBTB3* and *GRHL1* (Fig. 1d, Table 2). Like imprinted genes, the placental expression of several parentally biased genes followed tight gestational dynamics, e.g., high level of paternally biased *GRHL1*, *MCCC1*, *DNMT1*, and maternally biased *NLRP2* specifically in early pregnancy (Fig. 3, Additional file 9: Figure S2). No systematic deviations from biased parental allelic expression were detected in our dataset in the placentas representing term pregnancy complications.

### The majority of candidate imprinted genes detected exhibit biallelic expression in the human placenta

Robust biallelic expression in the human placenta was detected for 66 of 91 (72.5%) analyzed candidate imprinted genes (≥ 35 % of reads from both alleles; Figs. 1 and 2; Additional file 7: Table S5; Additional file 8: Table S6). The majority (92%) of the genes that were transcribed from both parental alleles are broadly expressed across tissues (47 genes) or exhibit enhanced transcription in other organ(s) than placenta (14 genes). The transcript levels of biallelic placental genes are variable and some of these loci exhibit either placenta-specific (*PAPPA2*, *LGALS14*) or enhanced (*AOC1, ASCL2*) expression.

## Discussion

This study represents the first systematic assessment of parental allelic expression of nearly 400 candidate imprinted genes in 54 human placental samples across all three trimesters of normal gestation and in cases of term preeclampsia, gestational diabetes, and fetal growth disturbances. Almost half of the candidate genes (*n* = 179; 45%) were either not transcribed or showed limited placental expression. Initial gene list was filtered for the presence of common exonic SNPs, sequencing depth, and informative families for the parental allelic expression. In total, 91 genes were retained for the final analysis. The detailed outcome data is presented as a catalog of parental allelic proportions and gene expression of all analyzed loci across human gestation and in term pregnancy complications (Additional file 9: Figure S2).

Only 11 of 91 analyzed genes (12.1%) showed confident signals of parent-of-origin-specific allelic expression in the human placenta and the programming of imprinting for all genes was stable across the entire gestation and assessed term pregnancy scenarios (Table 2; Fig. 2; Additional file 9: Figure S2). The strict requirement of a single copy dosage of these genes in the placental function appears to be conserved among mammals. *MEG3*, *PHLDA2*, *IGF2*, *H19*, *PEG10*, *DLK1*, and *MEST* have been classified as ancient imprinted genes as they are expressed with the same parent-of-origin manner in human, mouse, and equine placentas [34]. The confirmed genes with parental monoallelic expression are expressed specifically in the placenta or additionally only in the adrenal gland. High expression of the majority of imprinted genes in the second trimester of pregnancy supports their critical role in supporting the fine-tuning of developmental programming [35]. As a pronounced temporal dynamics pattern of gene expression across pregnancy was detected for each placental imprinted gene (Fig. 3, Additional file 9: Figure S2), gestational age-specific transcription has to be regulated independently of the programmed stable epigenetic imprints.

The restricted number of imprinted genes in the human placenta is consistent with the data on the mouse placenta [36] and other human tissues. Two independent studies on human tissues cataloged in the GTEx Project reached consistent conclusions that the majority of human imprinted genes are already known and the predicted number of loci with parent-origin-specific expression has been overestimated [7, 8]. The analysis of transcriptome-wide imprinting signals in 1582 samples representing 37 primary human tissues from 178 individuals reported only 42 high-confidence imprinted genes. Widespread tissue specificity and also a tissue-specific alternative choice of expressed parental allele for some genes (e.g., *IGF2*) was observed. A parallel study on an extended dataset of 45 tissues detected imprinting signals for 93 genes, but concluded that tissue-specific imprinting is rather rare. In the current study, 8 of 11 confirmed placental imprinted genes show parental monoallelic expression in the majority of human organs [7, 8]. Across all tissues, the most stable imprinting has been detected for maternally expressed *MEG3* and *H19* (Table 3). However, two placenta-specific genes (*PHLDA2, ZFAT*) exhibit biallelic, but low expression in other tissues and for *AIM1* no data has been reported apart from the placenta.

The current study identified also a distinct class of 14 genes that showed a systematic bias towards the enrichment of transcripts from one parental allele (65–90% of reads), but the parental allelic proportions did not correspond to the generally acknowledged definition of imprinting. These genes were characterized by broad expression across tissues, diverse functions and notable inter-individual variation of parental allelic proportions (Fig. 2, Additional file 9: Figure S2). The molecular mechanisms leading to biased parental allelic expression are still to be uncovered. These may likely overlap with the programming of imprints in fetal germ cells, and reflect differences in the epigenetic reprogramming of maternal and paternal pronuclei in fertilized oocytes and/or somatic chromosomal aberrations in early embryos involving preferably one parental chromosome. There is a support to all these scenarios. Some genes with biased parental expression in the placenta have been reported as imprinted in other organs, e.g., *ZDBF2* (many tissues), *GRB10* (brain), *MKRN3* (brain, esophagus) (Table 3) [7, 8]. It is also well established that the paternally derived chromosomes are actively demethylated by the TET3 enzymes, whereas the maternally derived chromosomes undergo passive, replication-dependent demethylation achieved by nuclear exclusion of DNMT1 [37–40]. The observed more conservative pattern of paternally compared to maternally biased genes is supporting the previously reported post-fertilization differences in epigenetic reprogramming of sperm- and oocyte-derived methylation marks [13] (Fig. 2). Oocyte-derived placenta-specific transiently differentially methylated regions (DMR) have been associated with polymorphic imprinting that is characteristic to the placentas of primates [12, 13]. Interestingly, these DMRs can adopt an unusual epigenetic signature combining DNA methylation with biallelic enrichment of H3K4 histone methylation that represents typically mutually exclusive epigenetic modifications [41]. Placental genome is hypomethylated [42] and prone to the promotion of somatic genomic changes [19], resembling the generation of chromosomal rearrangements typical in tumor tissues [43]. Interestingly, the placental somatic duplications have been reported to encompass a significant enrichment of imprinted, mostly maternally expressed genes [19]. On the other hand, some placental-biased genes such as *NUTD12* (paternal) and *NLRP2* (maternal) show often monoallelic, but non-parental expression in other tissues [7]. Additionally, the utilized short-read RNA-seq data may have misclassified the loci that encode both, non-imprinted transcripts and placenta-specific imprinted isoforms (e.g., *GRB10* [44]) Mapping the co-expressional reads of several transcripts would mask isoform-specific imprinting signals and the gene may be categorized as a parentally biased locus. Development of locus-specific assays to analyze individual transcriptional isoforms would clarify this issue.

In total, 48 of the analyzed genes had been proposed as novel candidate imprinted loci in recent placental genome-wide DNA methylation or small-scale RNA-Seq based studies [6, 14, 15]. Disappointingly, the current study could not confirm explicit parental monoallelic expression for any of these genes, and a robust biallelic transcription was detected for most loci (Additional file 7: Table S5; Additional file 8: Table S6; Additional file 9: Figure S2). Only a small fraction of these genes showed reliable evidence for biased parental allelic expression. Among the genes reported to harbor maternal differentially methylated regions (mDMR) [6, 45], preferred expression of paternal transcripts was detected for *MCCC1*, *DCAF10*, *DNMT1*, *NUDT12*, and *RHOBTB3* (Table 2)*.* Interestingly, for *KLHDC10* showing clearly maternally biased expression, mDMR has been reported within the gene body [14]. The discrepancy between the reported parent-of-origin allelic methylation vs. transcription is supported by the emerging evidence that in a number of genomic regions, constitutive parental DNA methylation imprints are actually decoupled from the parent of origin expression effects [13, 46]. Several studies have shown that candidate loci associated with placenta-specific maternal methylation are associated with actual parental allelic transcriptional bias at only half the loci [6, 13, 14]. Additionally, allelic imbalances in DNA methylation may reflect the underlying differences in primary DNA sequence [47, 48]. Concerning RNA-Seq-based studies, spurious claims of parental monoallelic expression may arise from modest informative sample sets, random sampling errors of transcript pools entering library preparation and RNA-sequencing, insufficient read coverage and limited QC (e.g., RNA-Seq mapping or genotyping errors), and loose statistical criteria in defining imprinted genes (reviewed in [7]). This may lead to false-positive claims of parental imprinting, especially for the genes with low transcript levels that are fine-tuned at the cellular level by non-parental random monoallelic expression (RMAE) [49, 50]. Furthermore, in clonal cell lines that are typical for the placenta, RMAE may be present for a notable subset of cells [51].

Placental imprinting errors have been associated with fetal growth disturbances and with maternal preeclampsia or gestational diabetes [3, 5, 10, 52] (Additional file 11: Table S8**)**. In our dataset, no systematic link was observed between term pregnancy pathologies and deviations of parental allelic proportions or expressional dynamics of imprinted and biased genes (Figs. 2 and 3; Additional file 7: Table S5; Additional file 9: Figure S2). However, we acknowledge that a modest number of analyzed samples representing each subgroup may have limited the ability to detect rare isolated clinical cases with altered imprinting. And in the other way round, the enrichment of placentas representing various scenarios of complicated pregnancies in our dataset may have skewed the analysis due to possible loss-of-imprinting in adverse gestational outcomes.

Also, the limitations of the study have to be acknowledged. The study approach relied on genotyped (vs. imputed) SNPs and applied stringent QC and filtering to minimize false positives claims and detect high-confidence imprinted genes. These procedures excluded from the analysis of 116 imprinting candidate genes (29.3% of the initial list) that are adequately expressed in the placenta.

## Conclusions

The study outcome suggested that true imprinting, defined as > 90% transcripts originating from one parental allele, is in the human placenta restricted to well-characterized loci. These genes demonstrated highly stable silencing of one parental gene copy and monoallelic expression of the other allele across gestation and in the analyzed term pregnancy complications. A distinct group of additional 14 genes exhibited a statistically significant bias in parental allelic proportions defined as having 65–90% of reads from one parental allele. The molecular mechanisms behind this phenomenon are still to be clarified. However, nearly 2/3 of the analyzed genes showed no signals of deviation from biallelic expression. Consistent with the data on other GTEx tissues, the number of human imprinted genes appears to be overestimated.

## Additional files

Additional file 1: Supplementary methods. (PDF 120 kb)

Additional file 2: **Table S1.** Additional information on the parental and offspring characteristics of placental samples representing term pregnancy. (PDF 77 kb)

Additional file 3: **Table S2.** Additional data on the terminated pregnancy cases subjected to the collection of first and second trimester placental samples. (PDF 59 kb)

Additional file 4: **Table S3.** Filtering pipeline for the imprinting candidate genes to be included in the analysis in the current study. (XLSX 36 kb)

Additional file 5: **Figure S1.** Types of informative families for the decision making regarding the parental origin of the placenta expressed alleles. (PDF 76 kb)

Additional file 6: **Table S4.** Primers used for RT-PCR validation experiments. (PDF 51 kb)

Additional file 7: **Table S5.** Parental read counts per each analyzed SNP in the placental RNA-Seq dataset across the full study sample and in the clinical subgroups. (XLSX 141 kb)

Additional file 8: **Table S6.** Binominal test results assessing the parental allelic proportions in the placental RNA-Seq dataset and background information on the placental expression level and overall expressional breadth of the analyzed genes across tissues. (XLSX 29 kb)

Additional file 9: **Figure S2.** Catalog of the parental allelic proportions and gene expression level of all analyzed 91 candidate imprinted genes across gestation (first, second, and third trimester normal pregnancy) and in cases of term pregnancy complications (preeclampsia, gestational diabetes, delivery of a small- or large-for-gestational-age newborn). (PDF 2660 kb)

Additional file 10: **Table S7.** Experimental validation of parent-of-origin-specific or biallelic expression of selected genes using RT-PCR, cloning, and sequencing. (PDF 68 kb)

Additional file 11: **Table S8.** Genes that exhibit imprinting or biased parental allelic expression: literature evidence for the link to pregnancy, fetal disorders, or human disease. (PDF 107 kb)

## Data Availability

All data generated or analyzed during this study are included in this published article and its supplementary information files.

## References

[1] Peters J (2014). The role of genomic imprinting in biology and disease: an expanding view. Nat Rev Genet..

[2] Maupetit-Méhouas S, Montibus B, Nury D, Tayama C, Wassef M, Kota SK (2016). Imprinting control regions (ICRs) are marked by mono-allelic bivalent chromatin when transcriptionally inactive. Nucleic Acids Res..

[3] Monk D, Mackay DJGG, Eggermann T, Maher ER, Riccio A (2019). Genomic imprinting disorders: lessons on how genome, epigenome and environment interact. Nat Rev Genet..

[4] Sanchez-Delgado M, Martin-Trujillo A, Tayama C, Vidal E, Esteller M, Iglesias-Platas I (2015). Absence of maternal methylation in biparental hydatidiform moles from women with NLRP7 maternal-effect mutations reveals widespread placenta-specific imprinting. PLOS Genet..

[5] Eggermann T, Perez de Nanclares G, Maher ER, Temple IK, Tümer Z, Monk D (2015). Imprinting disorders: a group of congenital disorders with overlapping patterns of molecular changes affecting imprinted loci. Clin Epigenetics..

[6] Court F, Tayama C, Romanelli V, Martin-Trujillo A, Iglesias-Platas I, Okamura K (2014). Genome-wide parent-of-origin DNA methylation analysis reveals the intricacies of human imprinting and suggests a germline methylation-independent mechanism of establishment. Genome Res..

[7] Baran Y, Subramaniam M, Biton A, Tukiainen T, Tsang EK, Rivas MA (2015). The landscape of genomic imprinting across diverse adult human tissues. Genome Res..

[8] Babak T, Deveale B, Tsang EK, Zhou Y, Li X, Smith KS (2015). Genetic conflict reflected in tissue-specific maps of genomic imprinting in human and mouse. Nat Genet..

[9] John RM (2017). Imprinted genes and the regulation of placental endocrine function: Pregnancy and beyond. Placenta..

[10] Monk D (2015). Genomic imprinting in the human placenta. Am J Obstet Gynecol..

[11] Noguer-Dance M, Abu-Amero S, Al-Khtib M, Lefevre A, Coullin P, Moore GE (2010). The primate-specific microRNA gene cluster (C19MC) is imprinted in the placenta. Hum Mol Genet..

[12] Hanna CW, Peñaherrera MS, Saadeh H, Andrews S, McFadden DE, Kelsey G (2016). Pervasive polymorphic imprinted methylation in the human placenta. Genome Res..

[13] Sanchez-Delgado M, Court F, Vidal E, Medrano J, Monteagudo-Sánchez A, Martin-Trujillo A (2016). Human oocyte-derived methylation differences persist in the placenta revealing widespread transient imprinting. PLoS Genet..

[14] Hamada H, Okae H, Toh H, Chiba H, Hiura H, Shirane K (2016). Allele-specific methylome and transcriptome analysis reveals widespread imprinting in the human placenta. Am J Hum Genet..

[15] Metsalu T, Viltrop T, Tiirats A, Rajashekar B, Reimann E, Kõks S (2014). Using RNA sequencing for identifying gene imprinting and random monoallelic expression in human placenta. Epigenetics..

[16] Sõber S, Reiman M, Kikas T, Rull K, Inno R, Vaas P (2015). Extensive shift in placental transcriptome profile in preeclampsia and placental origin of adverse pregnancy outcomes. Sci Rep..

[17] Sõber S, Rull K, Reiman M, Ilisson P, Mattila P, Laan M (2016). RNA sequencing of chorionic villi from recurrent pregnancy loss patients reveals impaired function of basic nuclear and cellular machinery. Sci Rep..

[18] Reiman M, Laan M, Rull K, Sõber S (2017). Effects of RNA integrity on transcript quantification by total RNA sequencing of clinically collected human placental samples. FASEB J..

[19] Kasak L, Rull K, Vaas P, Teesalu P, Laan M (2015). Extensive load of somatic CNVs in the human placenta. Sci Rep..

[20] Kasak L, Rull K, Sõber S, Laan M (2017). Copy number variation profile in the placental and parental genomes of recurrent pregnancy loss families. Sci Rep..

[21] Sildver K. Sünnikaalukõverad Eestis ja sünnikaalu mõjutavad tegurid : registripõhine uuring: University of Tartu; 2014. http://rahvatervis.ut.ee/bitstream/1/5829/1/Sildver2014.pdf

[22] Kim D, Pertea G, Trapnell C, Pimentel H, Kelley R, Salzberg SL (2013). TopHat2: accurate alignment of transcriptomes in the presence of insertions, deletions and gene fusions. Genome Biol..

[23] Anders S, Pyl PT, Huber W (2014). HTSeq–A Python framework to work with high-throughput sequencing data HTSeq–A Python framework to work with high-throughput sequencing data. Bioinformatics..

[24] Kersey PJ, Allen JE, Armean I, Boddu S, Bolt BJ, Carvalho-Silva D (2016). Ensembl Genomes 2016: more genomes, more complexity. Nucleic Acids Res..

[25] Trapnell C, Williams BA, Pertea G, Mortazavi A, Kwan G, Van Baren MJ (2010). Transcript assembly and quantification by RNA-Seq reveals unannotated transcripts and isoform switching during cell differentiation. Nat Biotechnol..

[26] Jirtle RL. Geneimprint. http://www.geneimprint.com/. Accessed 25 May 2018.

[27] Kinsella RJ, Kahari A, Haider S, Zamora J, Proctor G, Spudich G (2011). Ensembl BioMarts: A hub for data retrieval across taxonomic space. Database..

[28] Li H (2011). A statistical framework for SNP calling, mutation discovery, association mapping and population genetical parameter estimation from sequencing data. Bioinformatics..

[29] Robinson JT, Thorvaldsdóttir H, Winckler W, Guttman M, Lander ES, Getz G (2011). Integrative genomics viewer. Nat Biotechnol..

[30] Hall TA (1999). BioEdit: a user-friendly biological sequence alignment editor and analysis program for Windows 95/98/NT. Nucl Acids Symp Ser..

[31] Uhlén M, Fagerberg L, Hallström BM, Lindskog C, Oksvold P, Mardinoglu A (2015). Tissue-based map of the human proteome. Science.

[32] Sekita Y, Wagatsuma H, Nakamura K, Ono R, Kagami M, Wakisaka N (2008). Role of retrotransposon-derived imprinted gene, Rtl1, in the feto-maternal interface of mouse placenta. Nat Genet..

[33] NCBI Gene. https://www.ncbi.nlm.nih.gov/gene. Accessed 25 May 2018.

[34] Wang XX, Miller DC, Harman R, Antczak DF, Clark AG, Wang XX (2013). Paternally expressed genes predominate in the placenta. Proc Natl Acad Sci..

[35] Uusküla L, Männik J, Rull K, Minajeva A, Kõks S, Vaas P (2012). Mid-gestational gene expression profile in placenta and link to pregnancy complications. PLoS One..

[36] Okae H, Hiura H, Nishida Y, Funayama R, Tanaka S, Chiba H (2012). Re-investigation and RNA sequencing-based identification of genes with placenta-specific imprinted expression. Hum Mol Genet..

[37] Mayer W, Niveleau A, Walter J, Fundele R, Haaf T (2000). Demethylation of the zygotic paternal genome. Nature..

[38] Iqbal K, Jin S-G, Pfeifer GP, Szabó PE (2011). Reprogramming of the paternal genome upon fertilization involves genome-wide oxidation of 5-methylcytosine. Proc Natl Acad Sci..

[39] Gu TP, Guo F, Yang H, Wu HP, Xu GF, Liu W (2011). The role of Tet3 DNA dioxygenase in epigenetic reprogramming by oocytes. Nature..

[40] Howell CY, Bestor TH, Ding F, Latham KE, Mertineit C, Trasler JM (2001). Genomic imprinting disrupted by a maternal effect mutation in the Dnmt1 gene. Cell..

[41] Monteagudo-Sánchez A, Sánchez-Delgado M, Mora JRH, Santamaría NT, Gratacós E, Esteller M (2019). Differences in expression rather than methylation at placenta-specific imprinted loci is associated with intrauterine growth restriction. Clin Epigenetics..

[42] Schroeder DI, Blair JD, Lott P, Yu HOK, Hong D, Crary F (2013). The human placenta methylome. Proc Natl Acad Sci..

[43] Martin-Trujillo A, Vidal E, Monteagudo-Sánchez A, Sanchez-Delgado M, Moran S, Hernandez Mora JR (2017). Copy number rather than epigenetic alterations are the major dictator of imprinted methylation in tumors. Nat Commun..

[44] Monk D, Arnaud P, Frost J, Hills FA, Stanier P, Feil R (2009). Reciprocal imprinting of human GRB10 in placental trophoblast and brain: evolutionary conservation of reversed allelic expression. Hum Mol Genet..

[45] Yuen RKKCK, Jiang R, Peñaherrera MS, McFadden DE, Robinson WP, Peaherrera MS (2011). Genome-wide mapping of imprinted differentially methylated regions by DNA methylation profiling of human placentas from triploidies. Epigenetics Chromatin..

[46] de Sá Machado Araújo G, da Silva Francisco Junior R, dos Santos Ferreira C, Mozer Rodrigues PT, Terra Machado D, Louvain de Souza T (2018). Maternal 5mCpG imprints at the PARD6G-AS1 and GCSAML differentially methylated regions are decoupled from parent-of-origin expression effects in multiple human tissues. Front Genet..

[47] Onuchic V, Lurie E, Carrero I, Pawliczek P, Patel RYY, Rozowsky J (2018). Allele-specific epigenome maps reveal sequence-dependent stochastic switching at regulatory loci. Science.

[48] Delahaye F, Do C, Kong Y, Ashkar R, Salas M, Tycko B (2018). Genetic variants influence on the placenta regulatory landscape. PLoS Genet..

[49] Chess A (2012). Mechanisms and consequences of widespread random monoallelic expression. Nat Rev Genet..

[50] Reinius B, Sandberg R (2015). Random monoallelic expression of autosomal genes: stochastic transcription and allele-level regulation. Nat Rev Genet..

[51] Morcos L, Ge B, Koka V, Lam KCCL, Pokholok DK, Gunderson KL (2011). Genome-wide assessment of imprinted expression in human cells. Genome Biol..

[52] Moore GE, Ishida M, Demetriou C, Al-Olabi L, Leon LJ, Thomas AC (2015). The role and interaction of imprinted genes in human fetal growth. Philos Trans R Soc B Biol Sci..

