# Supplementary material for: Parent-of-origin-specific allelic expression in the human placenta is limited to established imprinted loci and it is stably maintained across pregnancy
Source: Clin Epigenetics. 2019 Jun 26;11:94. doi: 10.1186/s13148-019-0692-3 (PMC6595585; doi:10.1186/s13148-019-0692-3)
Supplement: Supplementary file 1 — Supplementary methods. (PDF 120 kb) [file 13148_2019_692_MOESM1_ESM.pdf]

SUPPLEMENTARY METHODS

REPROMETA study material representing term pregnancies

REPROMETA (REPROgrammed fetal and/or maternal METAbolism) study material was collected at the Women's Clinic, Tartu University Hospital (TUH), Estonia (2006-2011) and it includes clinical information and biological samples from singleton term pregnancies delivered at 37-41 gestational weeks. The parental couples were recruited at the delivery room. The REPROMETA study aimed to recruit families representing diverse pregnancy outcomes at term regarding the absence/presence of maternal pregnancy-specific complications and the birth weight of a newborn. The sampleset comprises of five clinical subgroups: uncomplicated pregnancies resulting in the birth of newborn with the weight appropriate for-gestational age (normal term, birth-weight between 10–90 percentiles); maternal preeclampsia (PE); gestational diabetes (GD); delivery of a small- (SGA, birth weight <10th percentile) or a large-for-gestational age (LGA, birth weight >90th percentile) newborns. The weight percentiles for defining SGA and LGA were calculated on the basis of data from Estonian Medical Birth Registry Study [1]. All preeclampsia cases represented the severe form of late-onset PE pregnancies and were diagnosed in the presence of hypertension (systolic blood pressure ≥160 mmHg and/or diastolic blood pressure ≥110 mmHg) and/or proteinuria of ≥5 g in 24 hours. GD was diagnosed when 75 g oral glucose tolerance test performed at 24–28 weeks of gestation revealed either a fasting venous plasma glucose level of >5.1 mmol/l, and/or at 1 h and 2 h plasma glucose level of >10 mmol/l and >8.5 mmol/l glucose, respectively.

The biological sampling in the REPROMETA study included placenta, maternal and paternal blood samples, and umbilical cord blood serum. Information about mother's diseases, smoking, somatometric data, and childbirth history was obtained from medical records during the course of pregnancy and after birth. Fetal outcome data from delivery included weeks of gestation, birth weight, birth length, head and abdominal circumferences, and placental weight. Cases with documented fetal anomalies, chromosomal abnormalities, families with history of inherited diseases and patients with known pre-existing diabetes mellitus, chronic hypertension and chronic renal disease were excluded. Details of the REPROMETA pregnancies included into the current study are provided in **Additional file 2: Table S1**.

Placental sampling

Placental sampling was previously described in detail [2–4]. For term and 2nd trimester pregnancy sampling, placentas (stored at +4 °C for less than 1 h after delivery) were placed with the fetal side up on an absorbent pad. Approximately 2x2 cm block was excised through all layers of the middle region of placenta. Placental blocks were washed with 1×PBS to remove contamination of maternal blood. A piece of placenta was placed immediately into dry cryovial and stored at -80°C for DNA extraction. The rest of the sample was placed into 10 ml RNAlater solution (AM7021; Thermo Fisher Scientific, Waltham, MA, USA) and kept at −80 °C until RNA isolation. All samples were collected by the same medical personnel.

Samples from the 1st trimester placentas were obtained immediately after elective (surgical) termination of pregnancy and the procedure to purify chorionic villi is described previously by [5, 6]. The samples were washed with solution containing 15 ml Dulbecco's Phosphate Buffered Saline (PBS), 0.3 ml penicillin-streptomycin solution 10000U/10000µg/ml and 2 drops of heparine, 5000U/ml. The maternal cells were removed under a stereomicroscope (Discovery V8, Zeiss) and chorionic villi containing both cyto- and syncytiotrophoblast cells of fetal origin were placed into dry tube (for DNA analysis) or RNAlater solution (for RNA analysis) and stored at -80°C. For the 1st trimester samples, karyotyping was applied to confirm normal male or female karyotype.

RNA extraction, RNA-Seq library preparation and basic bioinformatics of raw sequencing data

Generation of the RNA-Seq dataset has been previously published [2, 4, 5]. Total RNA was extracted from 200-300 mg of homogenized placental tissue using TRIzol reagent (Invitrogen, Life Technologies) and further purified with RNeasy MinElute columns (Qiagen, Netherlands) according to the manufacturers' protocols. Purity level and concentration of isolated total RNA was measured using NanoDrop® ND-1000 UV-Vis spectrophotometer (Thermo Fisher Scientific, Waltham, MA, USA). Preparation of RNA-Seq sequencing libraries, sequencing of transcriptomes and basic bioinformatic processing of the raw sequencing data (quality control, read alignment and transcript and gene expression estimation) were performed at the Sequencing Unit of Finnish institute of Molecular Medicine (FIMM), University of

Helsinki, Finland. High quality DNA-free total RNA (5 µg) was used for depletion of ribosomal RNA (Ribo-Zero rRNA Removal Kit, MRZH11124; Illumina, San Diego, CA, USA). The rRNA depleted RNA was purified (NucleoSpin® RNA Clean-up XS, Macherey-Nagel, Duren, Germany) and reverse transcribed to double-stranded cDNA (SuperScript™ Double-Stranded cDNA Synthesis Kit, Life Technologies, Carlsbad, CA, USA). Random hexamers (New England BioLabs, Ipswich, MA, USA) were used for priming the first strand synthesis reaction and SPRI beads (Agencourt AMPure XP, Beckman Coulter, Brea, CA, USA) for purification of cDNA. Nextera™ Technology (Illumina, San Diego, CA, USA) was used for preparation of RNA-Seq libraries. In order to add the Illumina specific bridgePCR compatible sites as well as bar codes and enrich the library, limited-cycle PCR (5 cycles) was done according to instructions of Nextera system. SPRI beads were used for purification of the PCR-products and the library QC was evaluated by Agilent Bioanalyzer (Agilent Technologies, Santa Clara, CA, USA). C-Bot (TruSeq PE Cluster Kit v3, Illumina, San Diego, CA, USA) was used for cluster generation and Illumina HiSeq2000 platform (HiSeq TruSeq v3 reagent kit) for paired end sequencing with 2 x 46 bp read length (101 bp for two term placental samples). Each transcriptome library was loaded to occupy 1/3 of the lane capacity in a flow cell. Two samples were sequenced separately at ½ lane capacity and 2 x 101 bp read length.

Initial data analysis and preparation was conducted by the RNA-Seq pipeline v2.4 (FIMM) consisting of FastQC version 0.10.0 [7] for quality control. Reads were filtered for adaptor, rRNA and mtDNA sequences as well as homopolymer stretches using custom python scripts. Read alignment was performed with TopHat version 2.0.3 [8] using bowtie version 0.12.7 [9]. Transcript quantification (measured as FPKM) was conducted with Cufflinks v 2.0.2 [10] with reference annotation and gene expression as raw read counts was quantified by htseq-count [11]. Human genome assembly (GRCh37.p7/hg19) from Ensembl v67 was used as a reference. Read alignment to genomic regions was estimated with Picard v1.63 [12]. The complete dataset consisted of 2.35 billion paired-end reads (mean: 42.1 million per sample; range: 27.3- 74.6 million) with average alignment success rate of 82.7% (range: 56.2- 87.3%).

Genotyping of genomic DNA extracted from the placental and parental blood samples

Details of genotyping dataset used in this study have been previously reported [3, 6]. Placental and blood genomic DNA was genotyped using Illumina HumanOmniExpress-12-v1/24-v1 BeadChips (>715,000 markers with median spacing 2.1 kb) at the institutional genotyping core facility (Estonian Genome Center; http://www.geenivaramu.ee/en/core-facility). Samples were genotyped with an average overall call rate of 99.6% (median 99.7%).

Independent validation of the parental origin of transcripts by RT-PCR, cloning and sequencing

cDNA was synthesized from 1 µg total placental RNA according to the manufacturer's instructions (SuperScript III Reverse Transcriptase, Life Technologies). cDNA fragments were amplified by PCR from placental samples using PCR primers provided in **Additional file 6: Table S4**. The PCR primers were designed using a publicly available Primer3 software [13]. Unique match of the designed primers and the predicted RT-PCR products in the human transcriptome were assessed using BLASTN tool [14] with the options 'Human genomic + transcript (H G+T)' and 'Automatically adjust parameters for short input sequences'. To reach high-confidence conclusions about the *RTL1* gene, long-range PCR (2,357 bp) was designed to incorporate simultaneously two marker SNPs (rs3825569, rs6575805). PCR products were purified with Thermo Scientific GeneJet PCR purificaton Kit or gel-puried with MACHEREY-NAGEL NucleoSpin Gel and PCR Clean-up kit. All inserts were cloned into pCR™4-TOPO® TA vector (ThermoFisher Scientific TOPO® TA Cloning® Kits for Sequencing) according to the manufacturer's instructions, and the presence of the insert was confirmed by colony PCR using gene-specific PCR primer. Plasmid DNA was extracted with a MACHEREY-NAGEL NucleoSpin® Plasmid QuickPure or NucleoSpin® Plasmid EasyPure kit according to protocol. DNA concentrations were measured using NanoDrop ND-1000 UV-Vis spectrophotometer (NanoDrop Technologies, Inc., Wilmington, DE). Presence of the insert was confirmed by PCR using gene-specific PCR primer. Cloned inserts were sequenced using M13F and/or M13R primers at the institutional genotyping core facility (Estonian Genome Center; http://www.geenivaramu.ee/en/core-facility). Obtained sequences of inserts were visualized and analyzed with Bioedit software [14].

References to Supplementary Methods

1. Sildver K. Sünnikaalukõverad Eestis ja sünnikaalu mõjutavad tegurid : registripõhine uuring. University of Tartu; 2014. http://rahvatervis.ut.ee/bitstream/1/5829/1/Sildver2014.pdf.

2. Sõber S, Reiman M, Kikas T, Rull K, Inno R, Vaas P, et al. Extensive shift in placental transcriptome profile in preeclampsia and placental origin of adverse pregnancy outcomes. Sci Rep. 2015;5:13336. doi:10.1038/srep13336.

3. Kasak L, Rull K, Vaas P, Teesalu P, Laan M. Extensive load of somatic CNVs in the human placenta. Sci Rep. 2015;5:8342. doi:10.1038/srep08342.

4. Reiman M, Laan M, Rull K, Sõber S. Effects of RNA integrity on transcript quantification by total RNA sequencing of clinically collected human placental samples. FASEB J. 2017;31:3298–308. doi:10.1096/fj.201601031RR.

5. Sõber S, Rull K, Reiman M, Ilisson P, Mattila P, Laan M. RNA sequencing of chorionic villi from recurrent pregnancy loss patients reveals impaired function of basic nuclear and cellular machinery. Sci Rep. 2016;6:38439. doi:10.1038/srep38439.

6. Kasak L, Rull K, Sõber S, Laan M. Copy number variation profile in the placental and parental genomes of recurrent pregnancy loss families. Sci Rep. 2017;7:45327. doi:10.1038/srep45327.

7. Andrews S. FastQC: a quality control tool for high throughput sequence data. 2011. https://www.bioinformatics.babraham.ac.uk/projects/fastqc/.

8. Kim D, Pertea G, Trapnell C, Pimentel H, Kelley R, Salzberg SL. TopHat2: accurate alignment of transcriptomes in the presence of insertions, deletions and gene fusions. Genome Biol. 2013;14:R36. doi:10.1186/gb-2013-14-4-r36.

9. Langmead B, Trapnell C, Pop M, Salzberg SL. Ultrafast and memory-efficient alignment of short DNA sequences to the human genome. Genome Biol. 2009;10:R25. doi:10.1186/gb-2009-10-3-r25.

10. Trapnell C, Williams BA, Pertea G, Mortazavi A, Kwan G, Van Baren MJ, et al. Transcript assembly and quantification by RNA-Seq reveals unannotated transcripts and isoform switching during cell differentiation. Nat Biotechnol. 2010;28:511–5. doi:10.1038/nbt.1621.

11. Anders S, Pyl PT, Huber W. HTSeq – A Python framework to work with high-throughput sequencing data HTSeq – A Python framework to work with high-throughput sequencing data. Bioinformatics. 2014;31:0–5.

12. Picard. http://broadinstitute.github.io/picard.

13. Kõressaar T, Remm M. Enhancements and modifications of primer design program Primer3. Bioinformatics. 2007;23(10):1289–91. http://bioinfo.ut.ee/primer3.

14. BLASTN. https://blast.ncbi.nlm.nih.gov.

15. Hall TA. BioEdit: a user-friendly biological sequence alignment editor and analysis program for Windows 95/98/NT. Nucl Acids Symp Ser. 1999;41:95–8.
