# Supplementary material for: Parent-of-origin-specific allelic expression in the human placenta is limited to established imprinted loci and it is stably maintained across pregnancy
Source: Clin Epigenetics. 2019 Jun 26;11:94. doi: 10.1186/s13148-019-0692-3 (PMC6595585; doi:10.1186/s13148-019-0692-3)
Supplement: Supplementary file 2 — Table S1. Additional information on the parental and offspring characteristics of placental samples representing term pregnancy. (PDF 77 kb) [file 13148_2019_692_MOESM2_ESM.pdf]

Table S1. Additional information on the parental and offspring characteristics of placental samples representing term pregnancy.

	Normal term	SGA	LGA	PE	GD
Maternal characteristics					
Age (years)	33.0 (18-37)	24.5 (20-32)	30.0 (23-39)	26.5 (19-39)	32.5 (22-36)
BMI before pregnancy (kg/m$^{2)}$	23.8 (17.4-30.0)	21.1 (16.5-24.3)	24.2 (19.4-30.9)	26.0 (20.1-33.5)	25.5 (18.1-43.2)
Gestational weight gain (kg)	18.8 (11-20)	12.8 (9-20)	20 (13.5-33)	10 (6-16)	13 (8.1-25)
Parity (0/1/≥2)	3/4/1	7/1/0	2/2/4	6/1/1	3/3/2
Gestational age (days)	284.0 (260-291)	268.5 (264-289)	280.5 (275-288)	266.0 (260-271)	275.5 (268-284)
C- sect/ vaginal delivery (n)	5/3	6/2	3/5	2/6	3/5
Paternal characteristics					
Age (years)	34.0 (22-38)	26.0 (23-39)	35.5 (23-50)	32 (21-46)	34 (22-43)
BMI (kg/m^2)	24.9 (19.7-30.9)	23.1 (19.0-25.0)	28.4 (23.8-37.6)	29.1 (21.4-38.0)	27.2 (20.7-35.3)
Offspring characteristics					
Weight (g)	3756 (3102-4220)	2517 (2004-2698)	4744 (4420-4986)	2803 (2170-3570)	4284 (3940-4680)
Height (cm)	51.2 (48.5-54.5)	46 (45-48)	53.3 (52-55)	48 (45-49)	52.5 (51-54)
Placental weight (g)	575 (420-770)	420 (200-470)	817.5 (610-970)	462.5 (340-720)	587.5 (500-1060)
IUGR (n)	0	5	0	4	0
Sex, F/M (n)	3/5	5/3	4/4	4/4	5/3

Data is presented as median (range) if not indicated otherwise. The cases have been described in detail previously [1–3].

Normal term, pregnancy without complications until delivery at term; SGA, small-for-gestational-age; LGA, large-for-gestational age; PE, preeclampsia; GD, Gestational diabetes; Parity, number of deliveries before current pregnancy; IUGR, intrauterine growth restriction

References for Table S1

1. Sõber S, Reiman M, Kikas T, Rull K, Inno R, Vaas P, et al. Extensive shift in placental transcriptome profile in preeclampsia and placental origin of adverse pregnancy outcomes. Sci Rep. 2015;5:13336. doi:10.1038/srep13336.
2. Kasak L, Rull K, Vaas P, Teesalu P, Laan M. Extensive load of somatic CNVs in the human placenta. Sci Rep. 2015;5:8342. doi:10.1038/srep08342.
3. Reiman M, Laan M, Rull K, Sõber S. Effects of RNA integrity on transcript quantification by total RNA sequencing of clinically collected human placental samples. FASEB J. 2017;31:3298–308. doi:10.1096/fj.201601031RR.
