# Supplementary material for: Parent-of-origin-specific allelic expression in the human placenta is limited to established imprinted loci and it is stably maintained across pregnancy
Source: Clin Epigenetics. 2019 Jun 26;11:94. doi: 10.1186/s13148-019-0692-3 (PMC6595585; doi:10.1186/s13148-019-0692-3)
Supplement: Supplementary file 3 — Table S2. Additional data on the terminated pregnancy cases subjected to the collection of first and second trimester placental samples. (PDF 59 kb) [file 13148_2019_692_MOESM3_ESM.pdf]

Table S2. Additional data on the terminated pregnancy cases subjected to the collection of 1st and 2nd trimester placental samples.

	1st trimester	2nd trimester
Maternal age (years)	25.5 (18-33)	23.0 (15-36)
Maternal BMI before pregnancy (kg/m^2)	22.2 (19.4-29.7)	21.4 (16.9-25.2)
Parity (0/1/≥2)	2/5/1	4/0/2
Gestational age (days)	60.0 (51-81)	138.0 (126-167)
Fetal Sex, F/M (n)	5/3	3/3

Data is presented as median (range) if not indicated otherwise.

[a] 1st trimester chorionic villi were sampled at the elective surgical termination of pregnancy; 2nd trimester placental samples were derived from medically induced abortions due to maternal health indications (details in [1–3]).
BMI, Body Mass Index; Parity; number of pregnancies carried to viable gestational age.

References for Table S2

1. Kasak L, Rull K, Vaas P, Teesalu P, Laan M. Extensive load of somatic CNVs in the human placenta. Sci Rep. 2015;5:8342. doi:10.1038/srep08342.

2. Sõber S, Rull K, Reiman M, Ilisson P, Mattila P, Laan M. RNA sequencing of chorionic villi from recurrent pregnancy loss patients reveals impaired function of basic nuclear and cellular machinery. Sci Rep. 2016;6:38439. doi:10.1038/srep38439.

3. Kasak L, Rull K, Sõber S, Laan M. Copy number variation profile in the placental and parental genomes of recurrent pregnancy loss families. Sci Rep. 2017;7:45327. doi:10.1038/srep45327.
