# Supplementary material for: Parent-of-origin-specific allelic expression in the human placenta is limited to established imprinted loci and it is stably maintained across pregnancy
Source: Clin Epigenetics. 2019 Jun 26;11:94. doi: 10.1186/s13148-019-0692-3 (PMC6595585; doi:10.1186/s13148-019-0692-3)
Supplement: Supplementary file 5 — Figure S1. Types of informative families for the decision making regarding the parental origin of the placenta expressed alleles. (PDF 76 kb) [file 13148_2019_692_MOESM5_ESM.pdf]

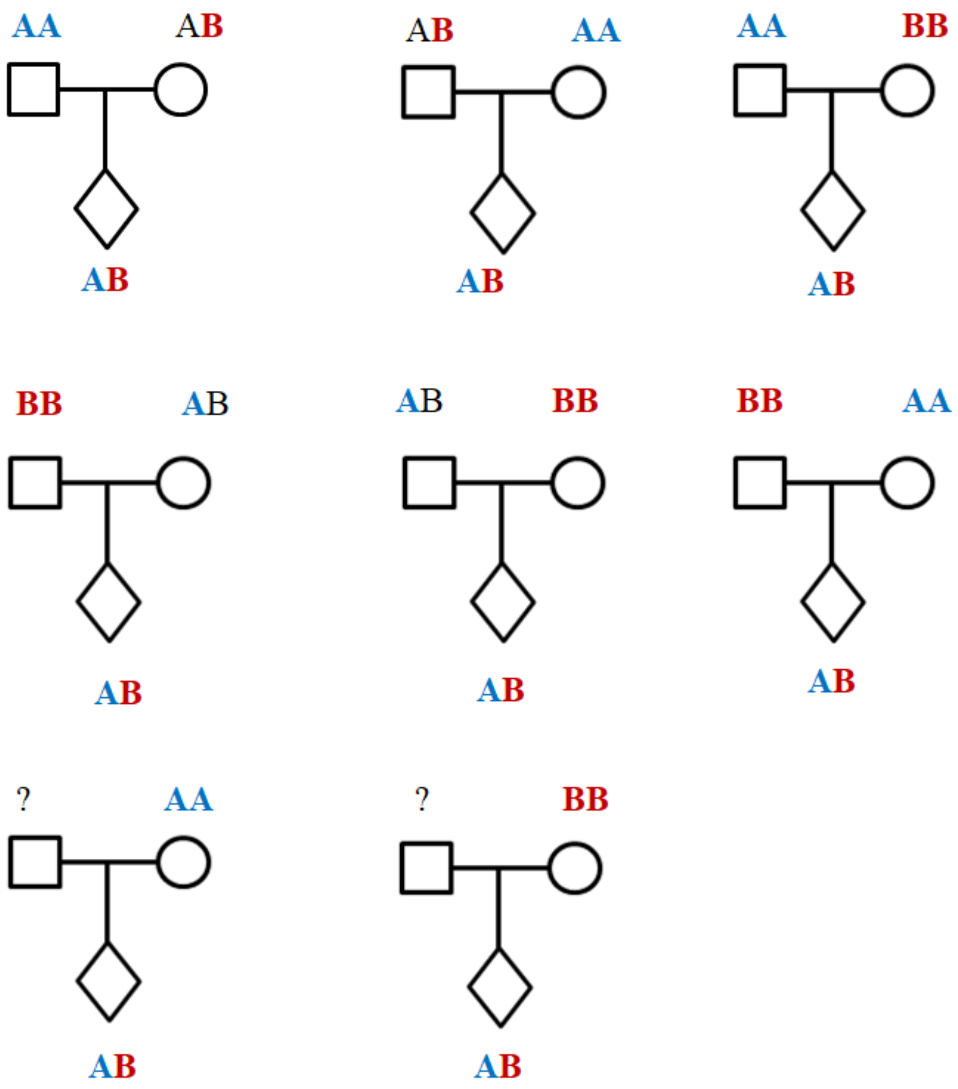

Figure S1. Types of informative families for the decision-making regarding the parental origin of the placenta expressed alleles. Family trios/duos were defined as informative if the placenta had heterozygous genotype of the SNP and at least one of the parents had homozygous genotype of this variant. Red (maternal) and blue (paternal) mark parent-of-origin of the inherited alleles.
