# Supplementary material for: Parent-of-origin-specific allelic expression in the human placenta is limited to established imprinted loci and it is stably maintained across pregnancy
Source: Clin Epigenetics. 2019 Jun 26;11:94. doi: 10.1186/s13148-019-0692-3 (PMC6595585; doi:10.1186/s13148-019-0692-3)
Supplement: Supplementary file 6 — Table S4. Primers used for RT-PCR validation experiments. (PDF 51 kb) [file 13148_2019_692_MOESM6_ESM.pdf]

Table S4. Primers used for RT-PCR validation experiments.

PCR Primer	Sequence 5' - 3'	Product length
DLK1_F	TCTGTGATAGAGATGTTCGGGC	494 bp
DLK1_R	GCGCTTCTTGACACAGGTGA	
RTL1_F	AGCACTCAACCGCAGAACTT	2,357 bp
RTL1_R	CTCGGTGTTCTCCAGGTAGC	
PAPPA2_002_F	GTATCACCACTGTCTTGTTTTGT	400 bp
PAPPA2_002_R	CCTTGACCCATAAACTCTTTTCC	
RHOBTB3_F	ATCTAATTGGGGGCGCTGAC	511 bp
RHOBTB3_R	AATACCACGTCCACACACTGG	

The PCR primers were designed using a publicly available Primer3 software (http://bioinfo.ut.ee/primer3/) (Kõressaar and Remm, 2007). Unique match of the designed primers and the predicted RT-PCR products in the human transcriptome were assessed using BLASTN platform (https://blast.ncbi.nlm.nih.gov/) with the options 'Human genomic + transcript (H G+T)' and 'Automatically adjust parameters for short input sequences'. F, Forward primer. R, Reverse primer

Reference:

Kõressaar T, Remm M. (2007) Enhancements and modifications of primer design program Primer3. Bioinformatics 23(10):1289-91.
