# Supplementary material for: Parent-of-origin-specific allelic expression in the human placenta is limited to established imprinted loci and it is stably maintained across pregnancy
Source: Clin Epigenetics. 2019 Jun 26;11:94. doi: 10.1186/s13148-019-0692-3 (PMC6595585; doi:10.1186/s13148-019-0692-3)

Figure S2. Catalogue of the parental allelic proportions and gene expression level of all analyzed 91 candidate imprinted genes across gestation (1st, 2nd and 3rd trimester normal pregnancy) and in cases of term pregnancy complications (preeclampsia, gestational diabetes, delivery of a small- or large-for-gestational-age newborn). Genes have been categorized as (A) imprinted, (B) biased and (C) biallelic loci. Within in each catergory, genes are presented in alphabetical order.

(A) Imprinted genes

AIM1

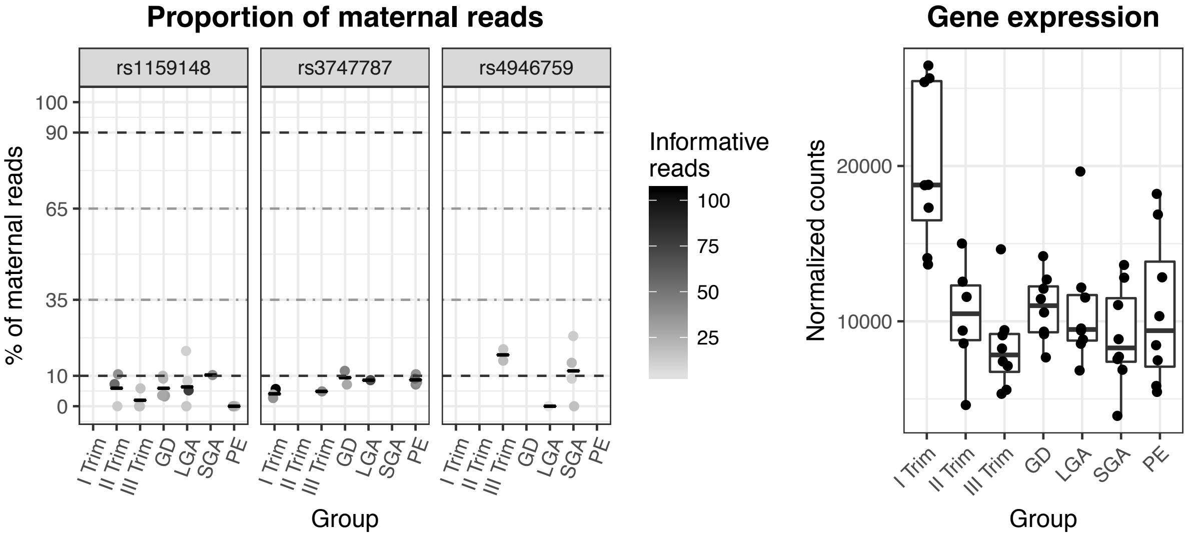

DLK1

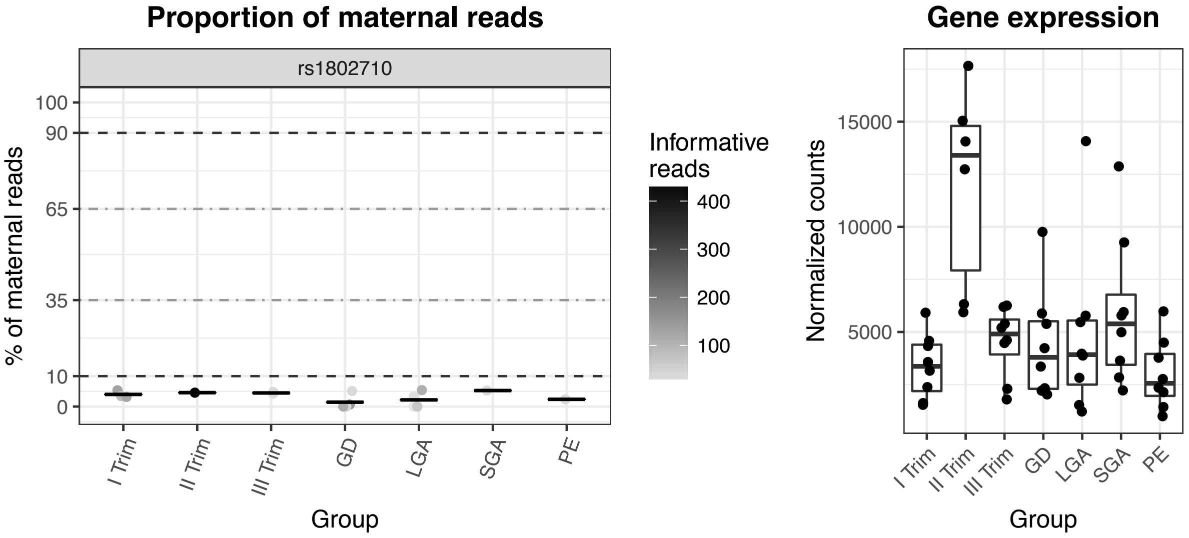

H19

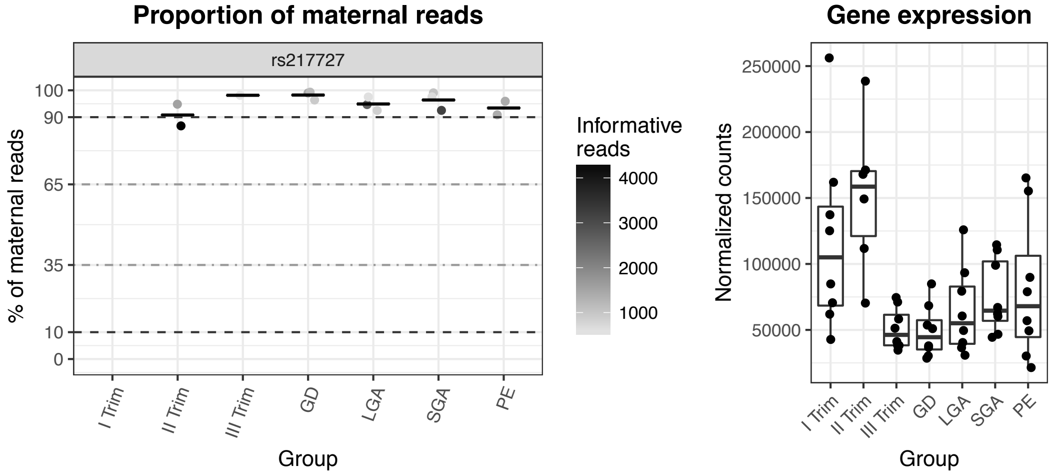

IGF2

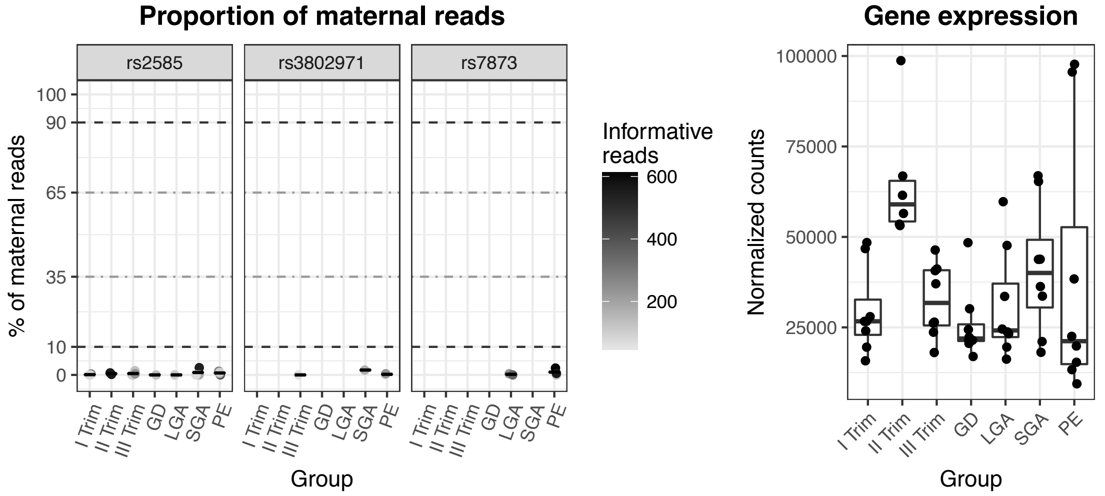

MEG3

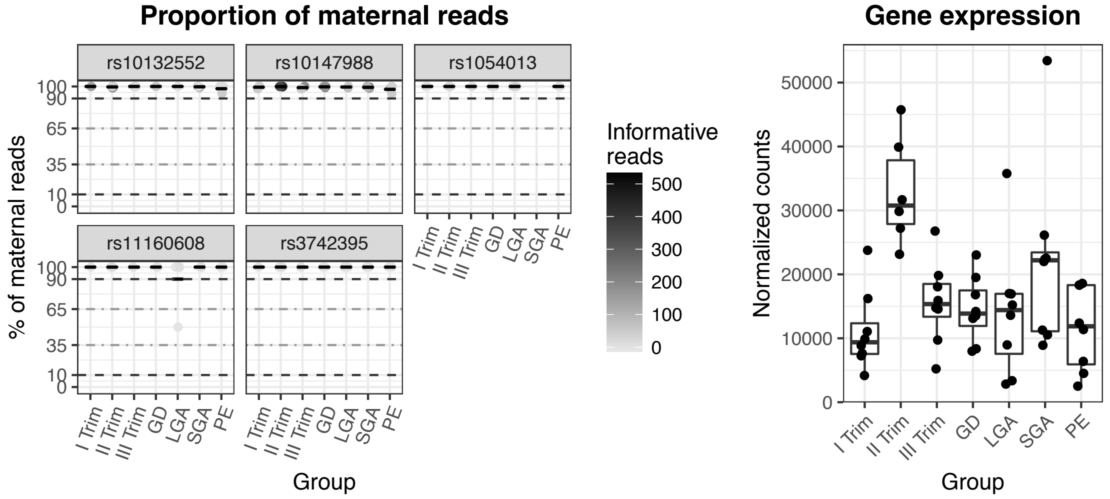

MEST

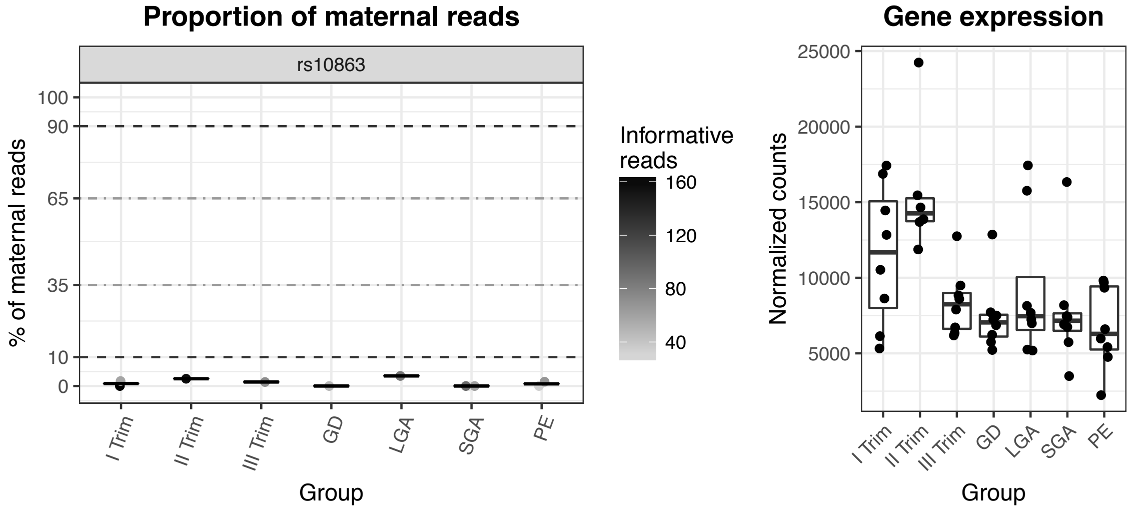

PEG10

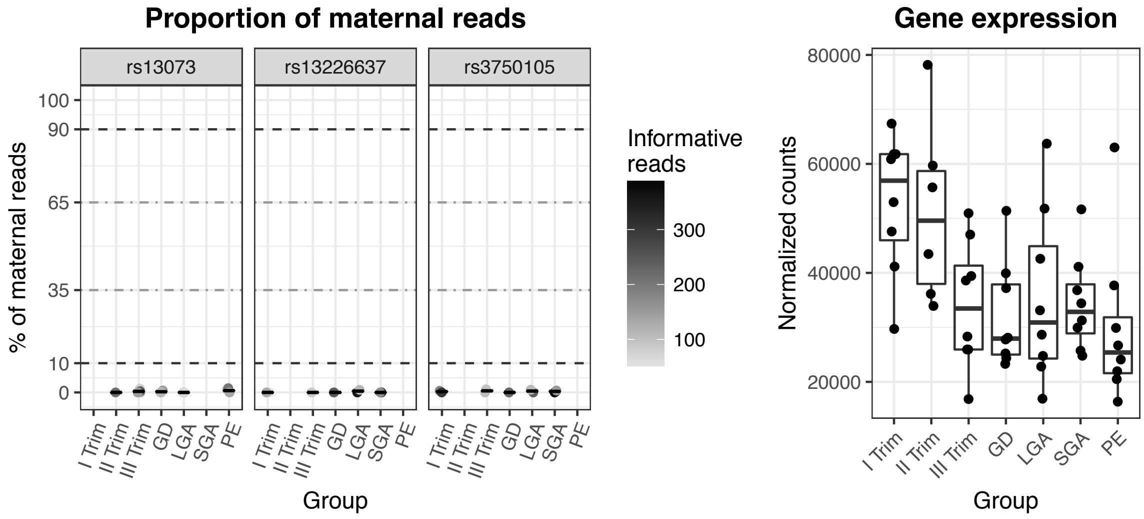

PHLDA2

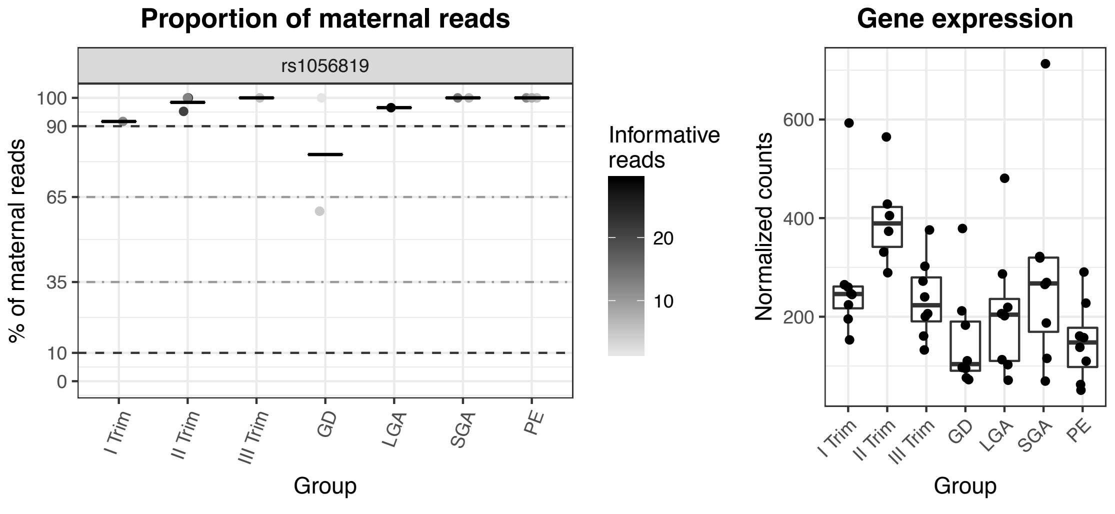

PLAGL1

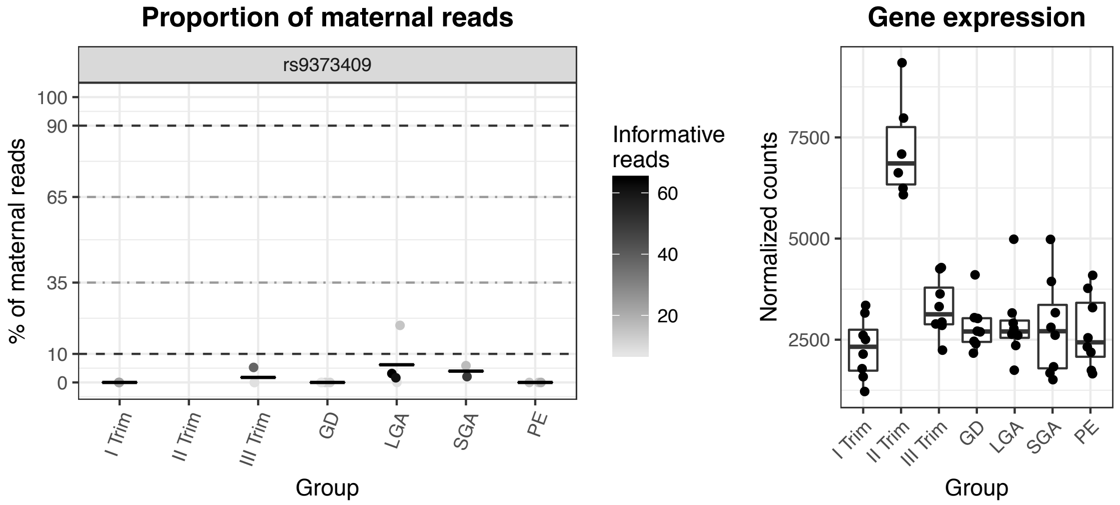

RTL1

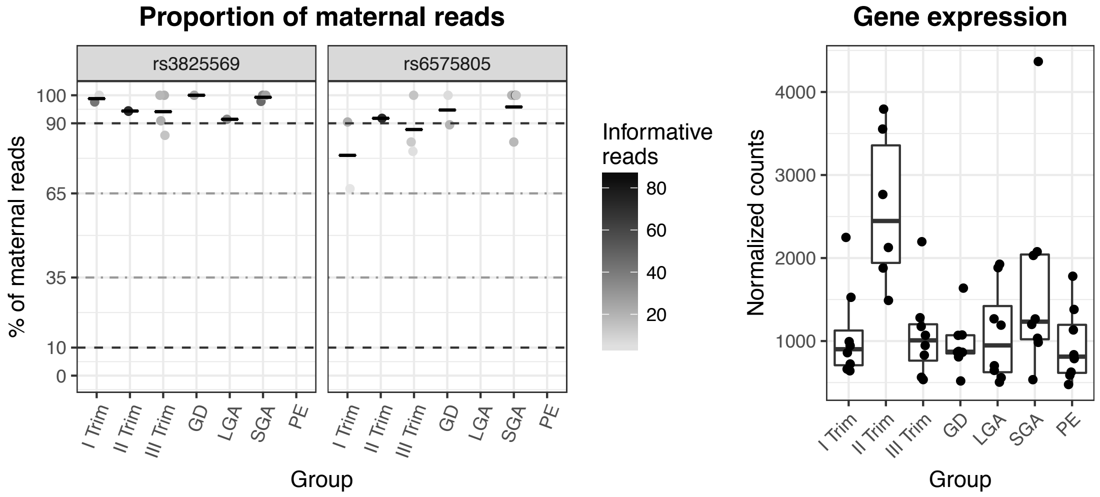

ZFAT

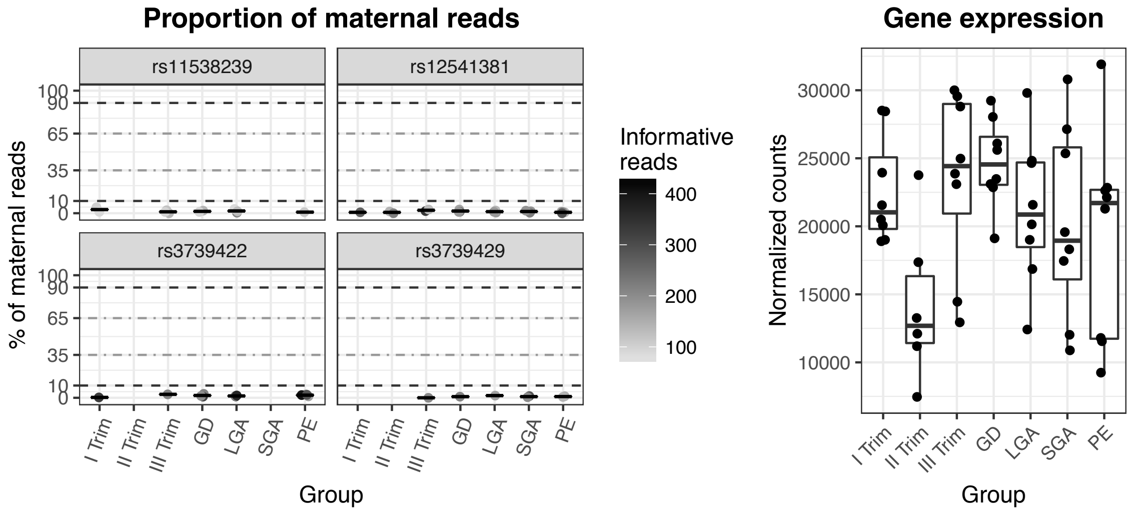

(B) Genes with biased parental allelic expression

CPXM2

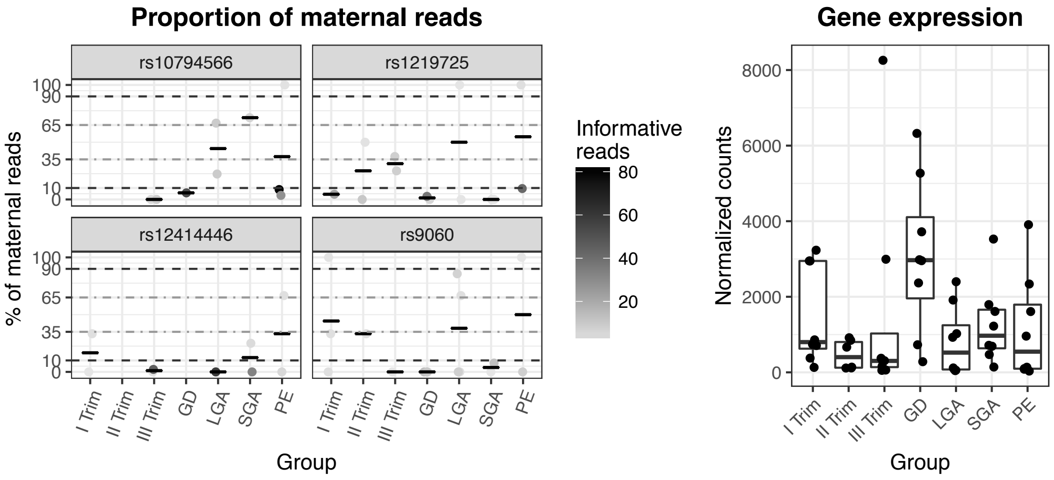

DCAF10

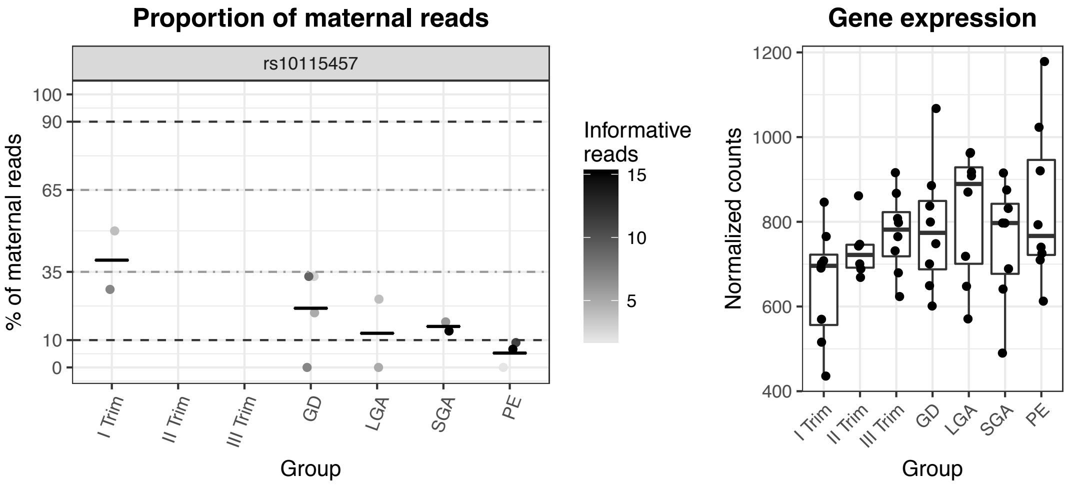

DNMT1

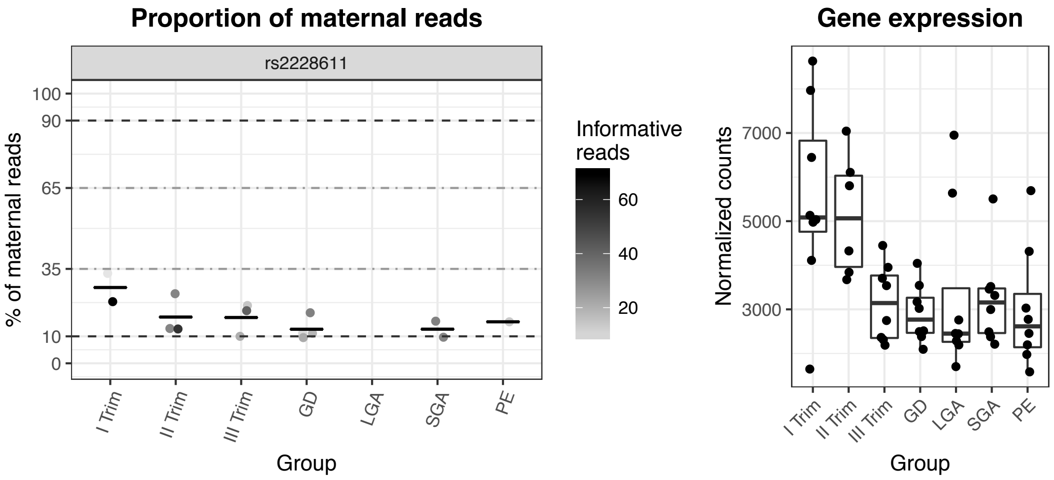

GRB10

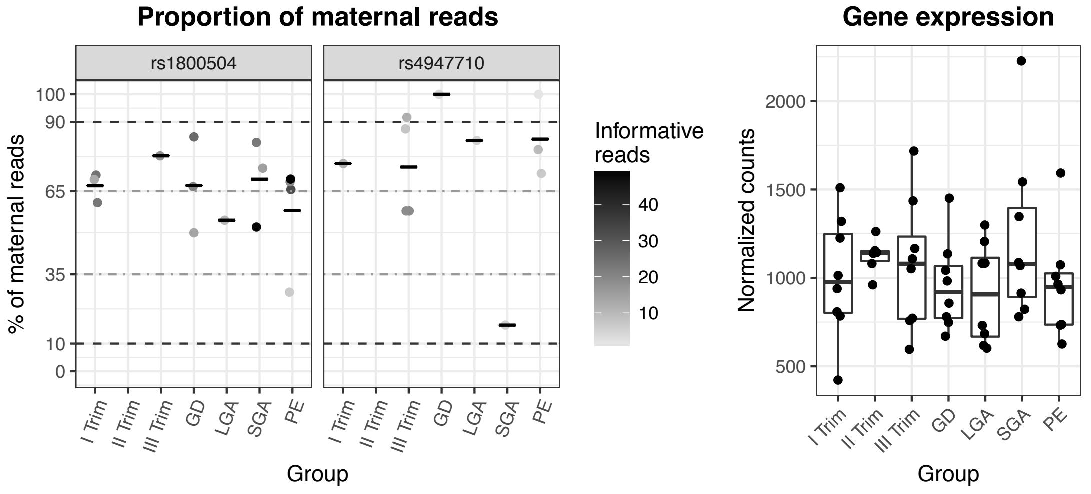

GRHL1

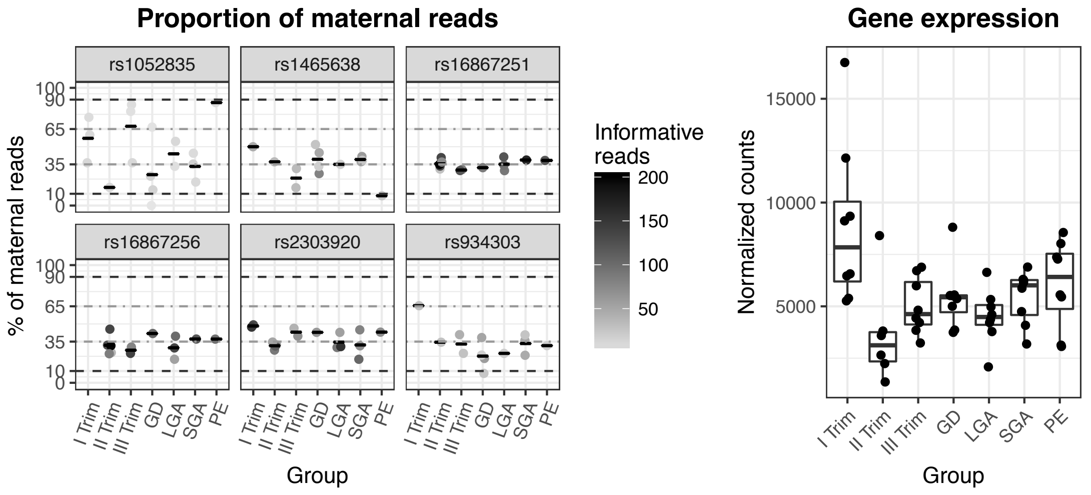

KLHDC10

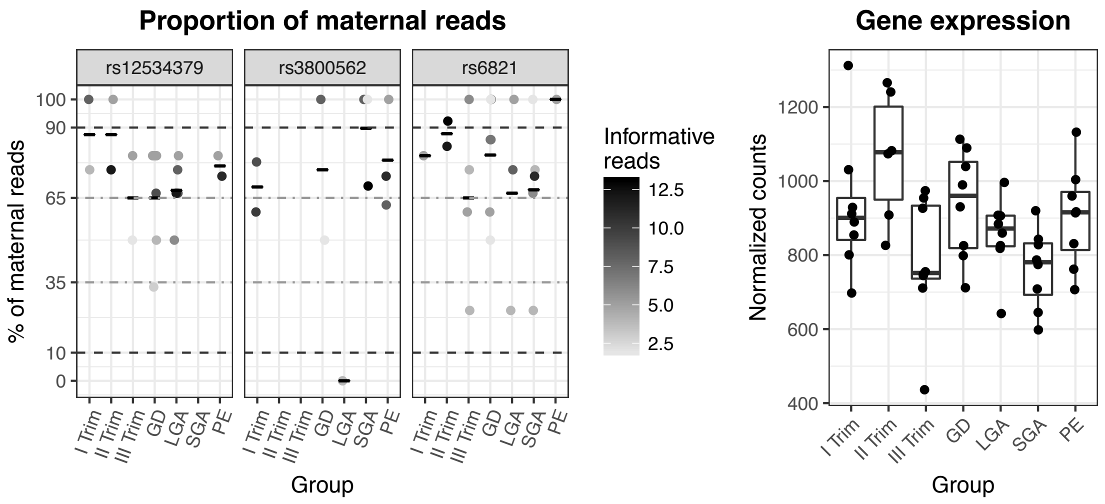

MCCC1

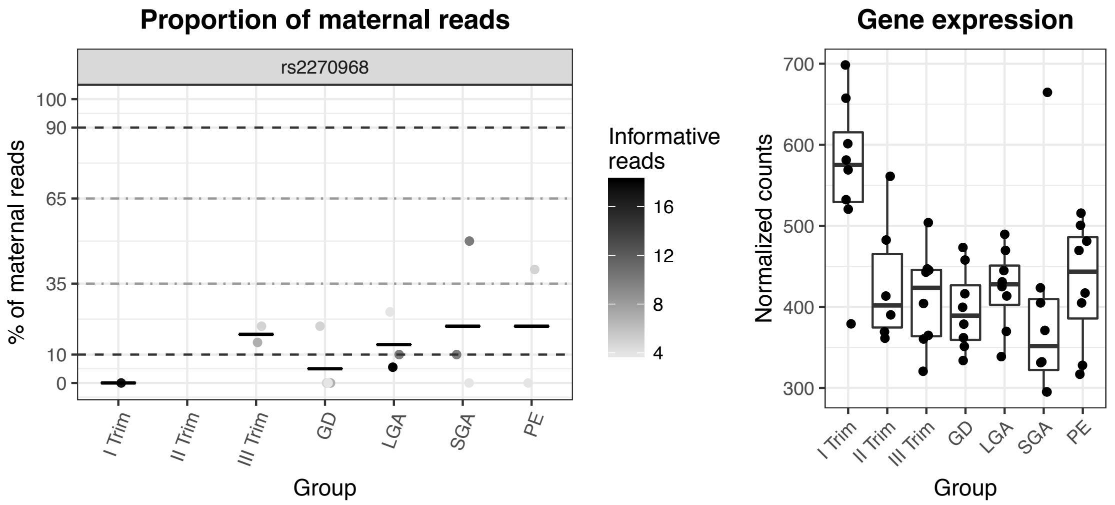

MKRN3

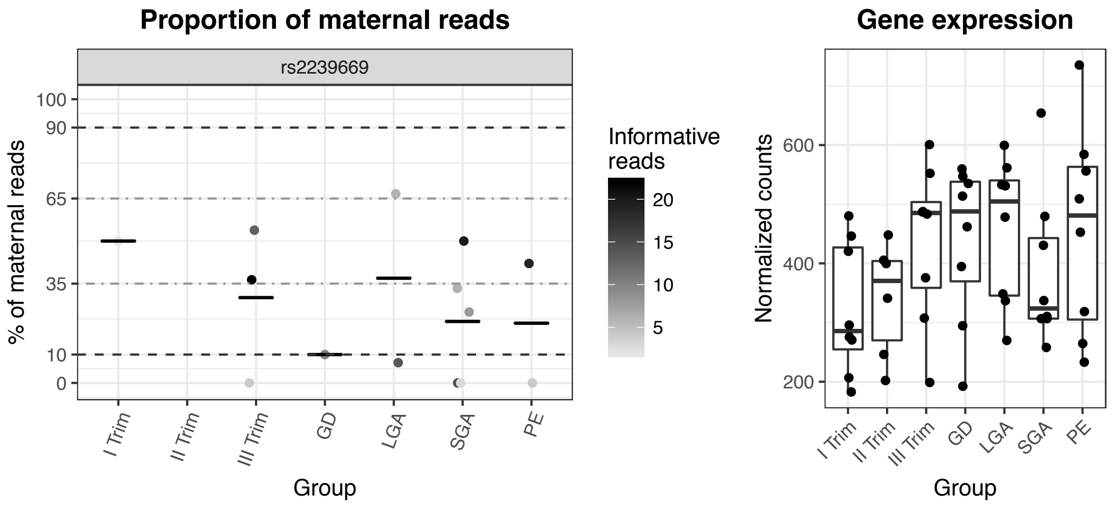

NAA60

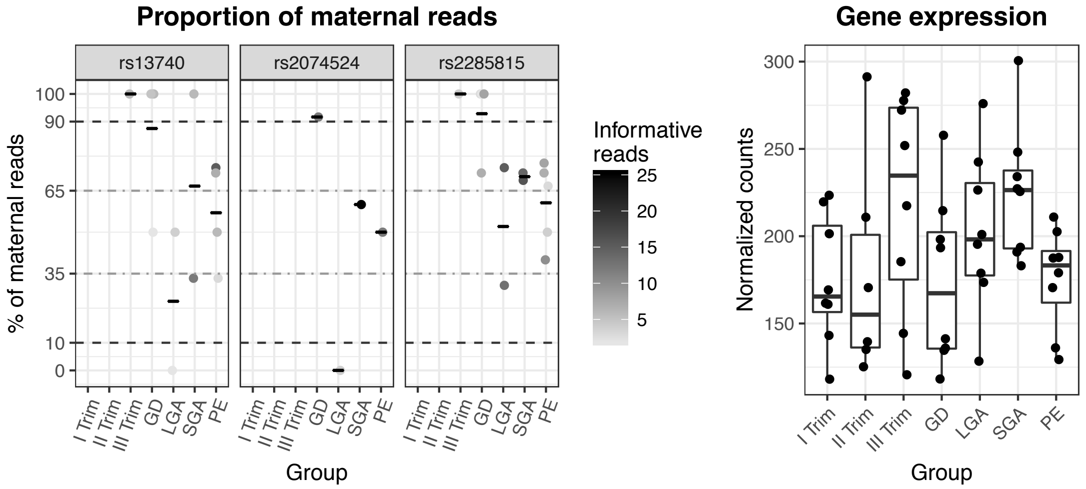

NLRP2

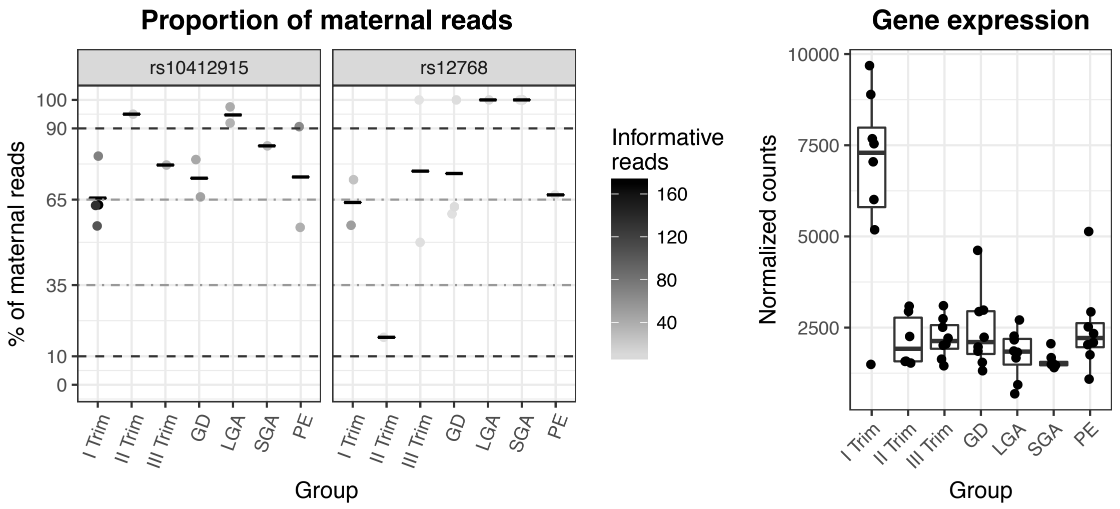

NUDT12

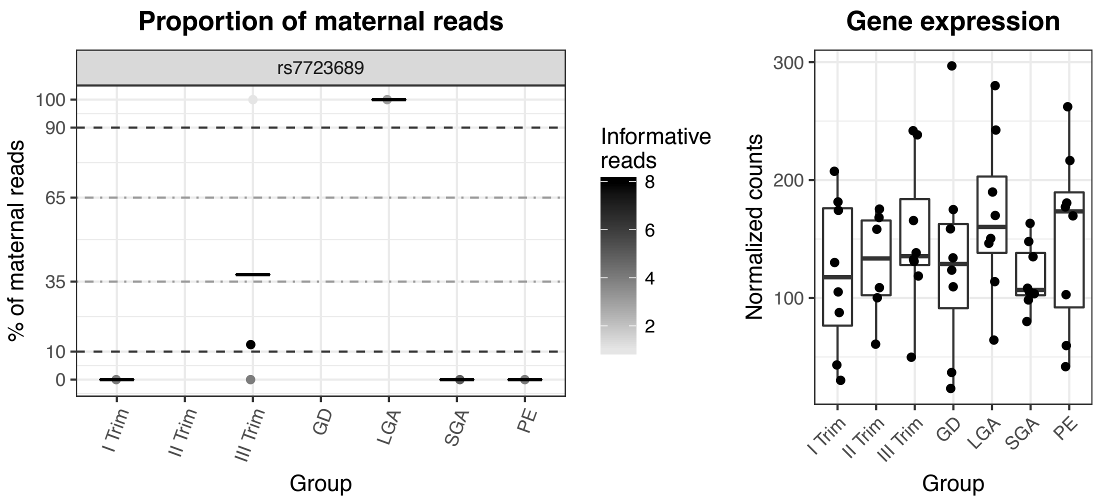

PLEKHG4B

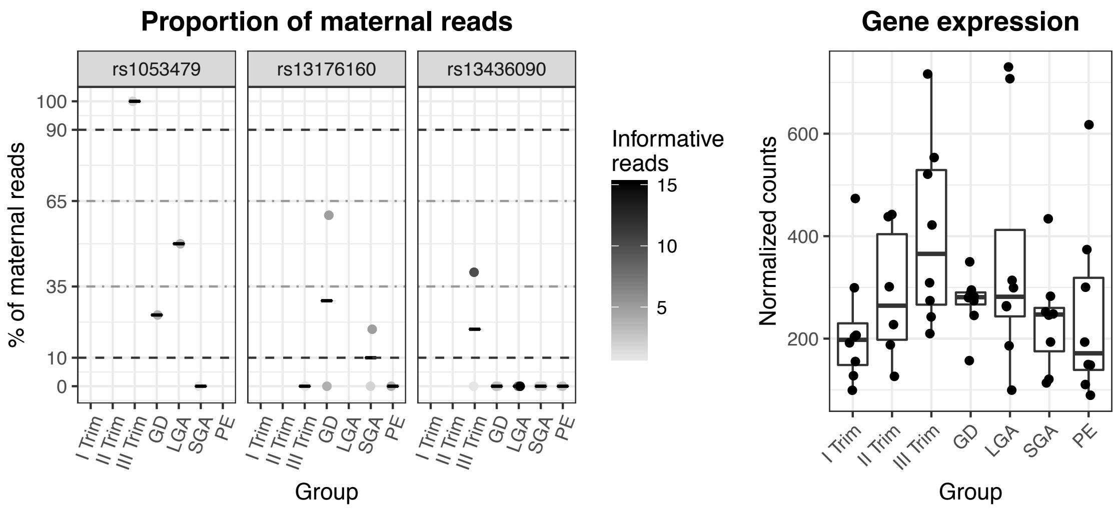

RHOBTB3

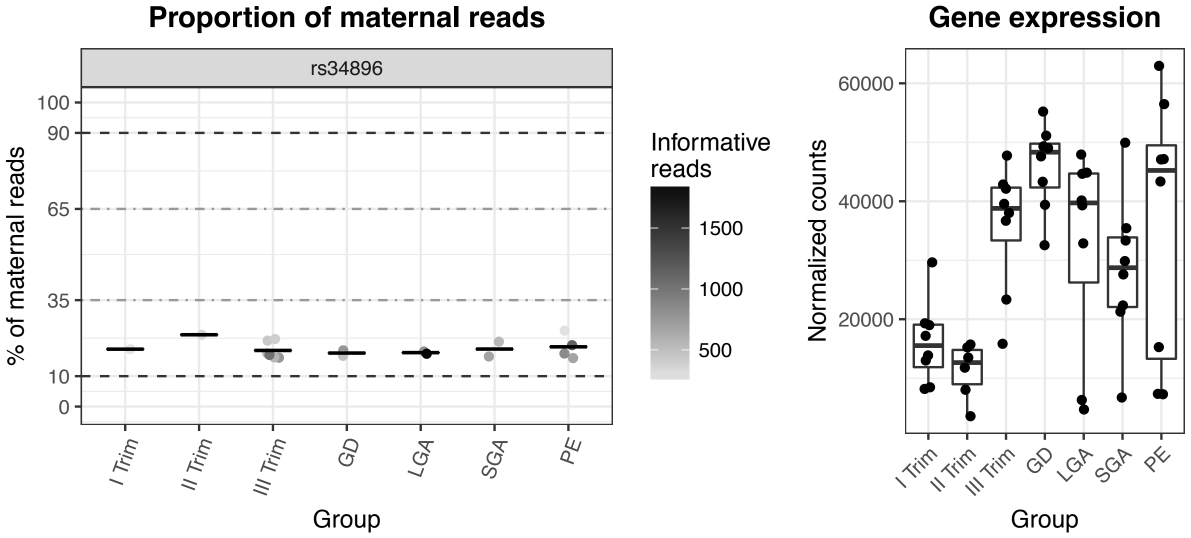

ZDBF2

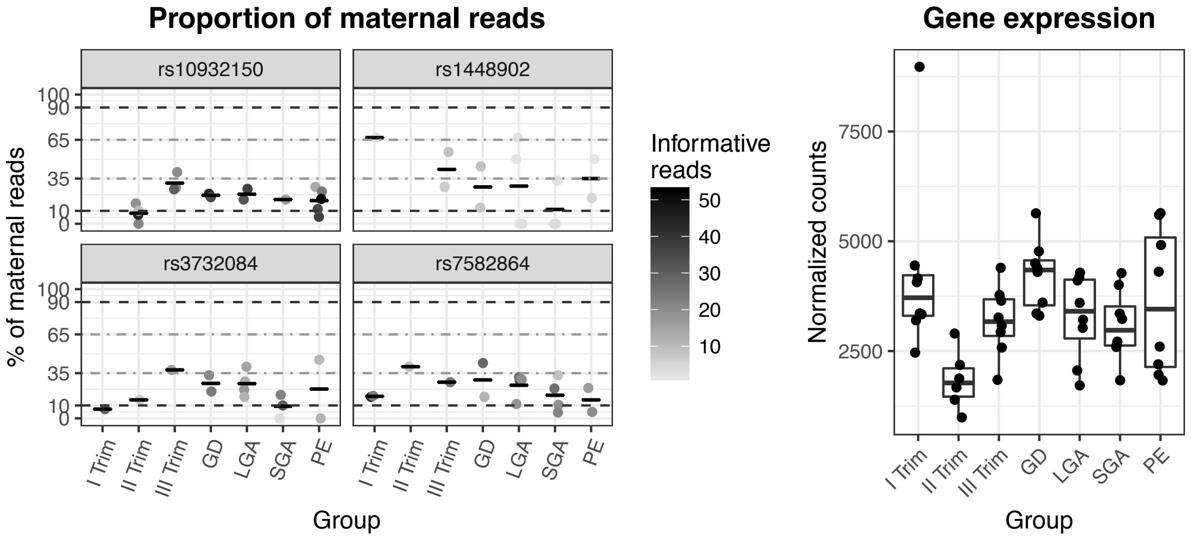

(A) Genes with biallelic parental allelic expression

AIFM2

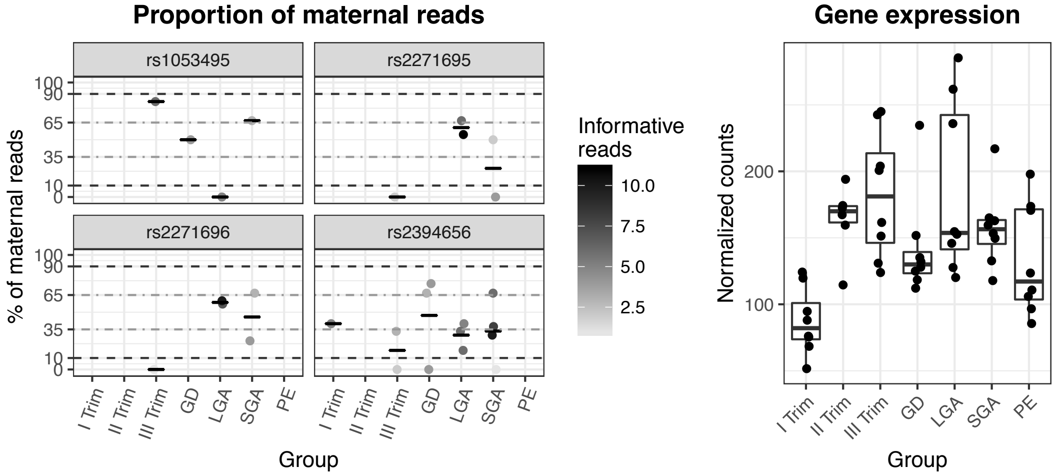

AMPD3

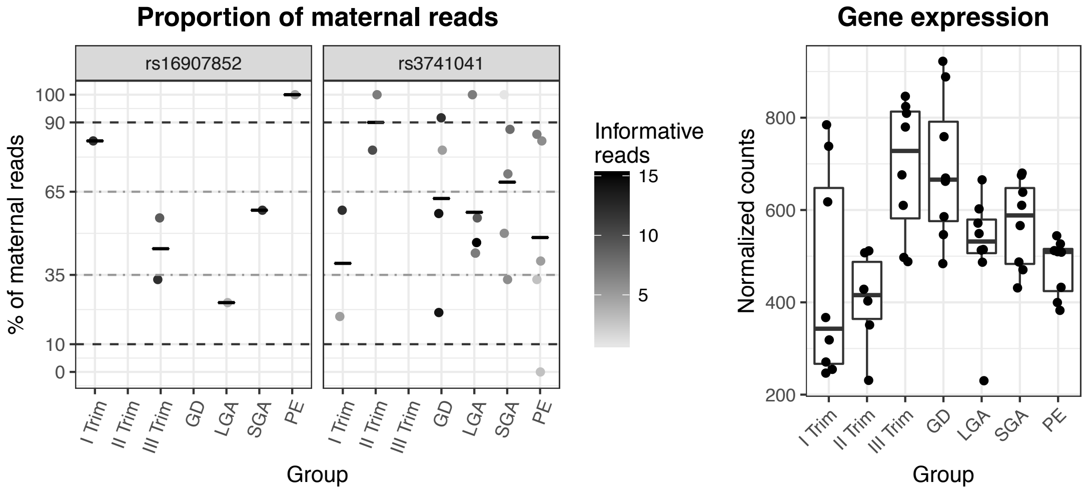

ANO1

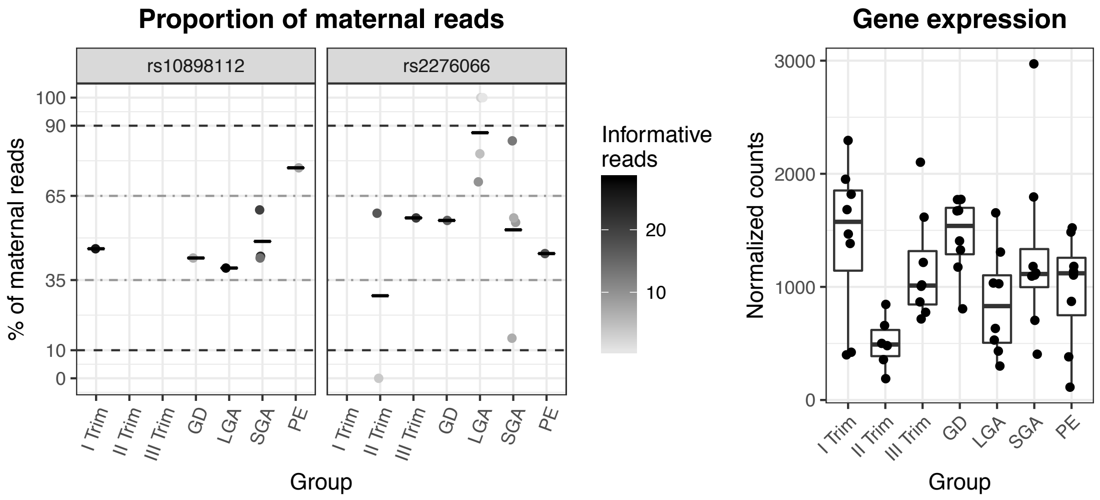

AOC1

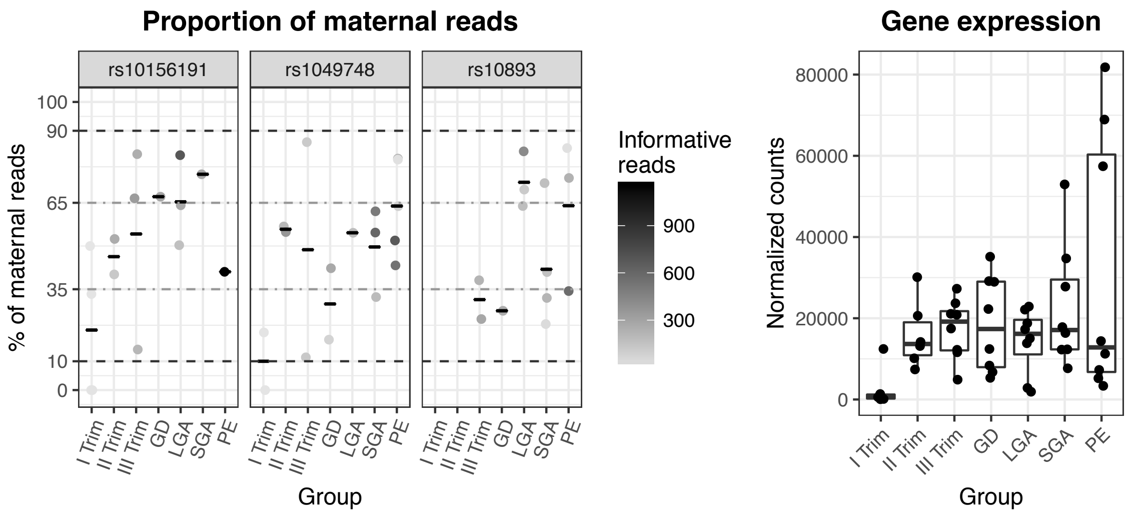

ASCL2

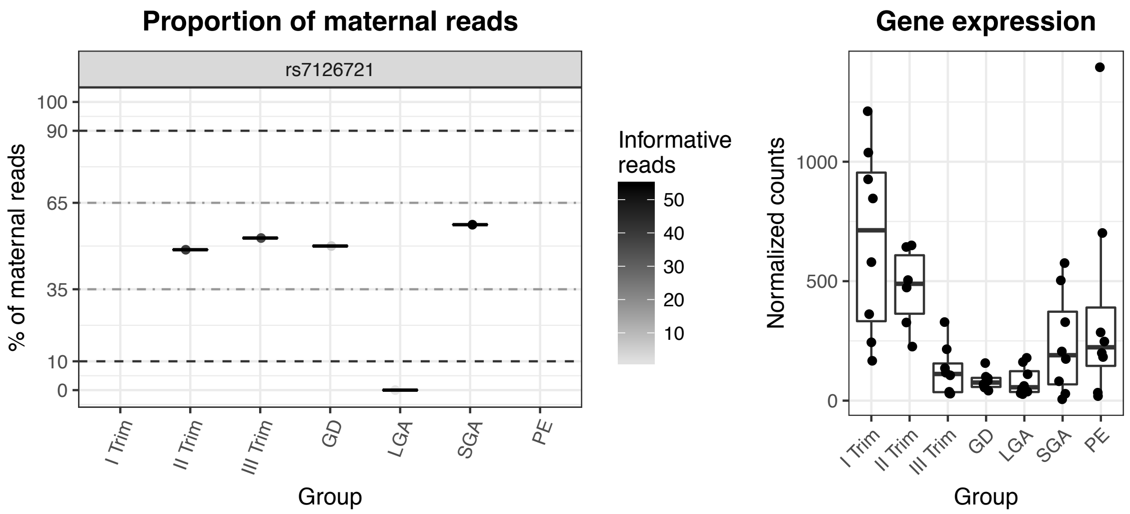

CASC5

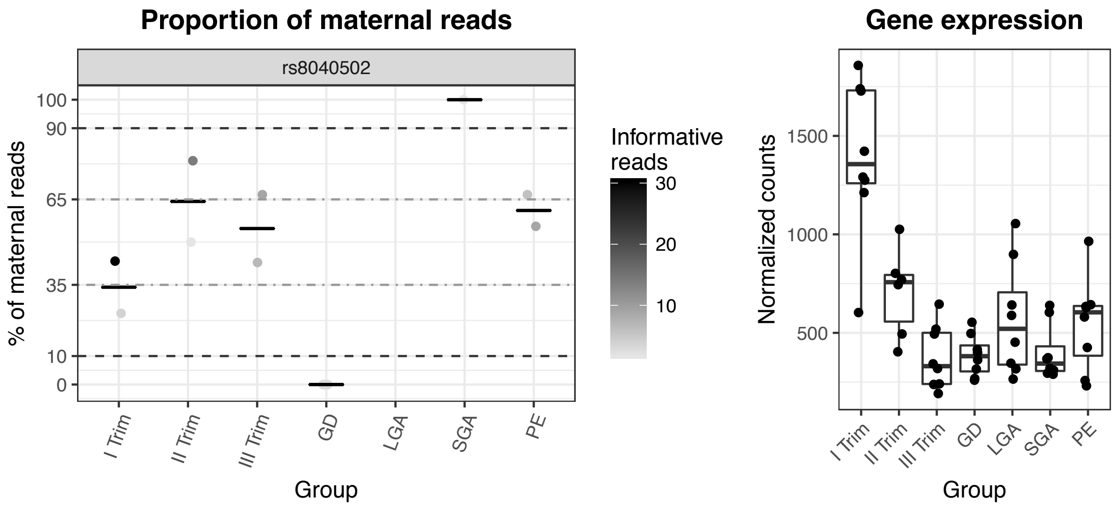

CBR1

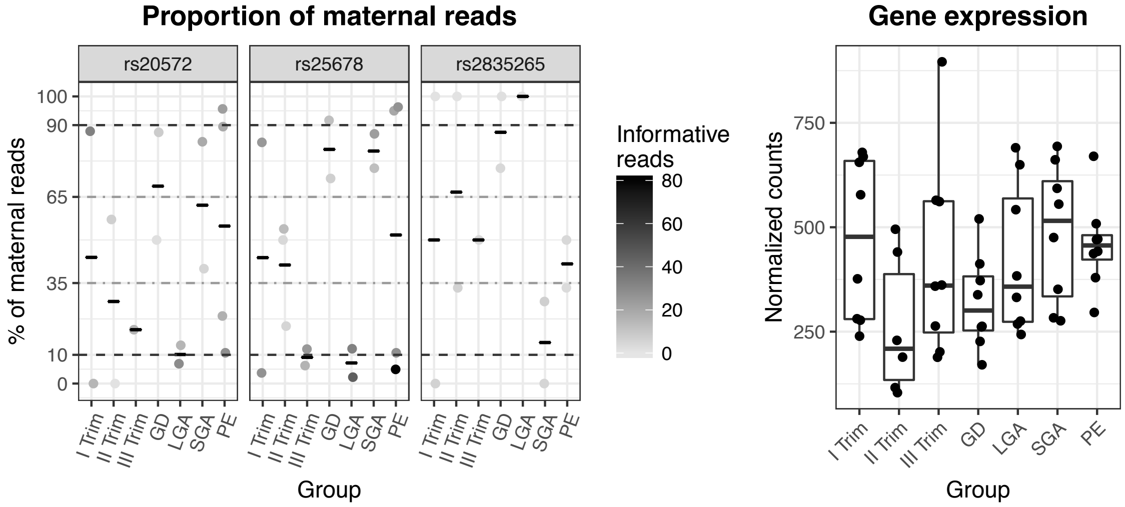

CCDC85A

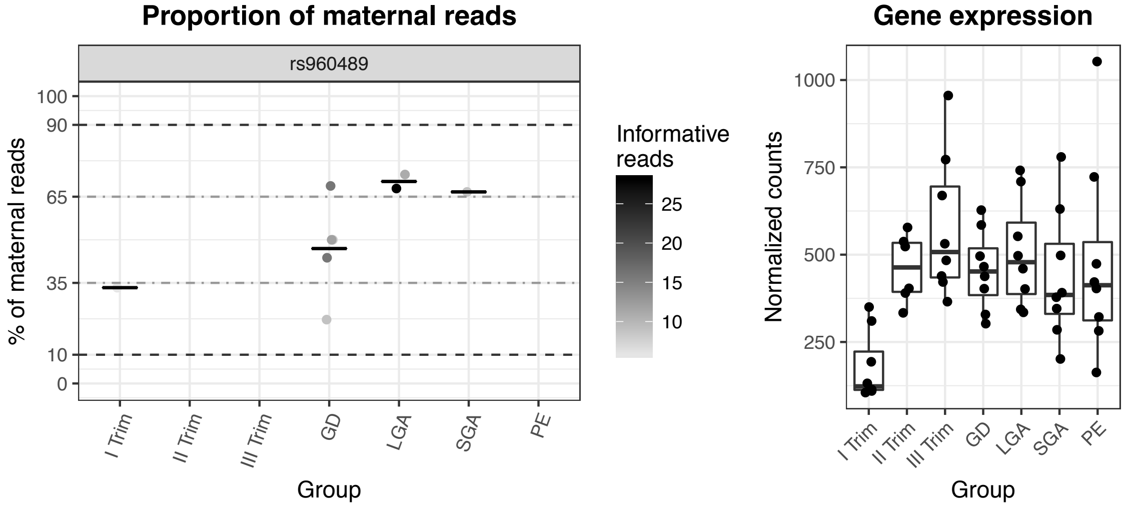

CEP63

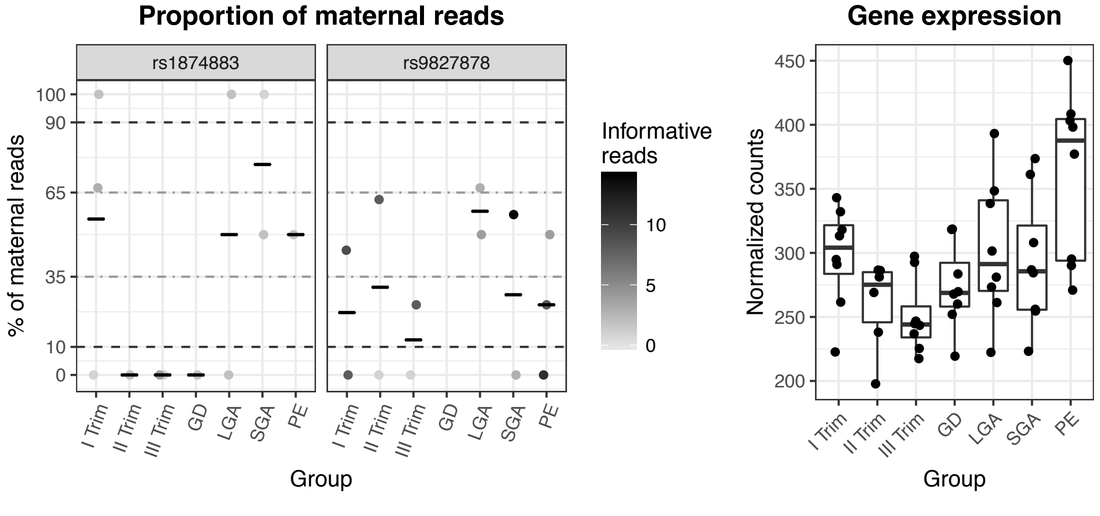

CHMP2A

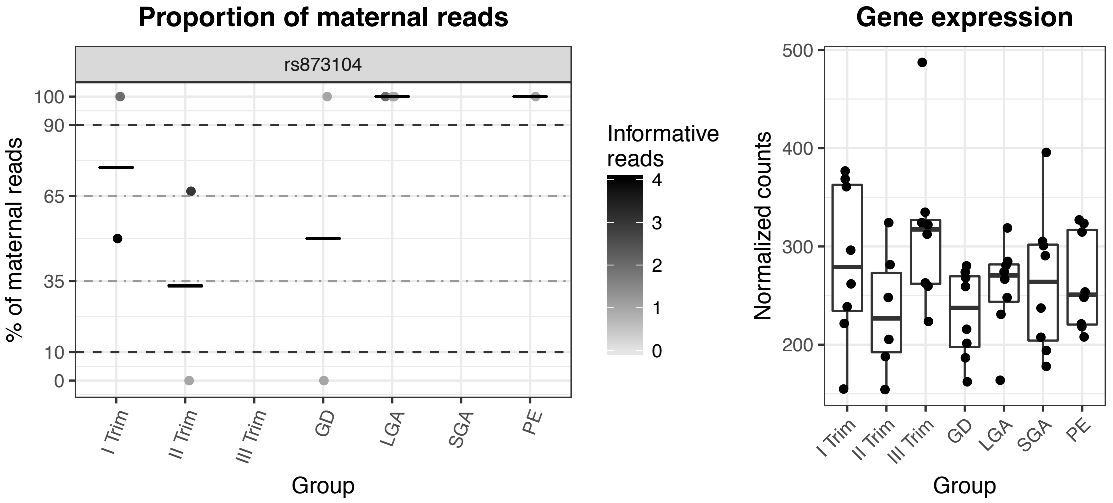

CLASP1

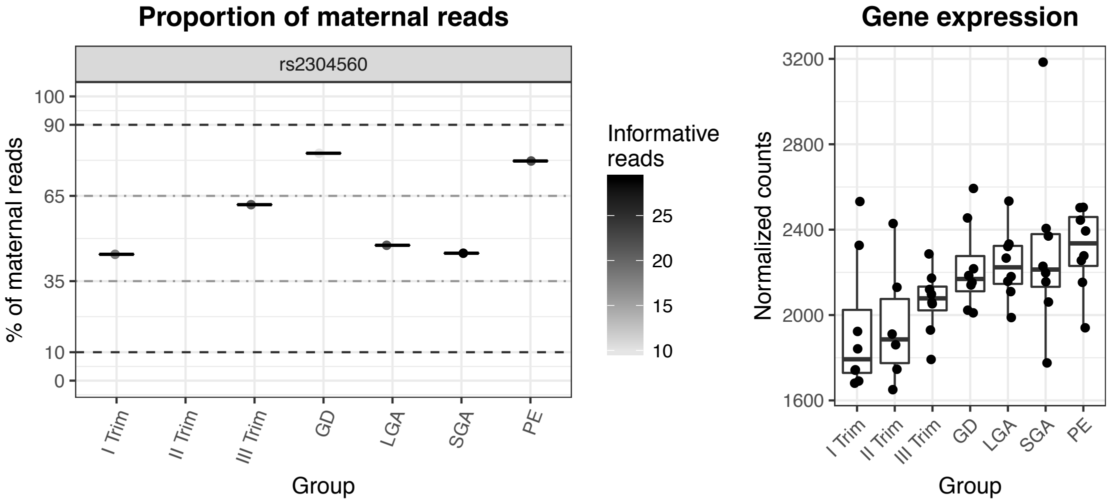

CLDN23

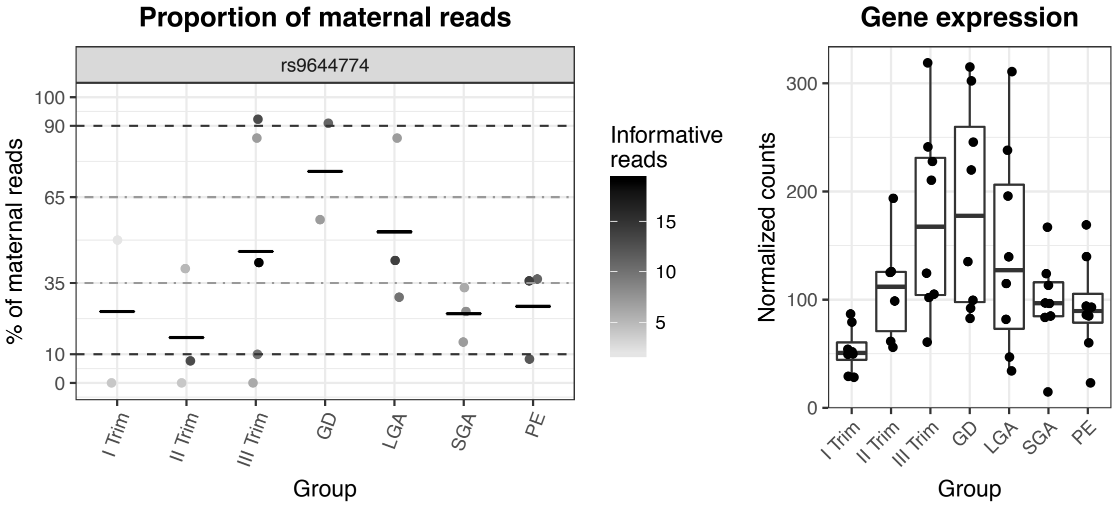

CUL7

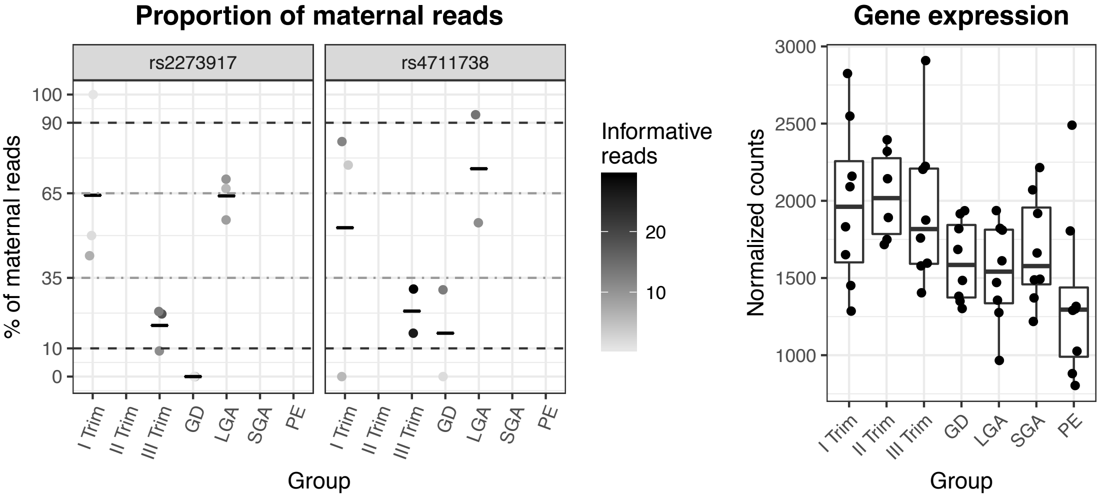

DHCR7

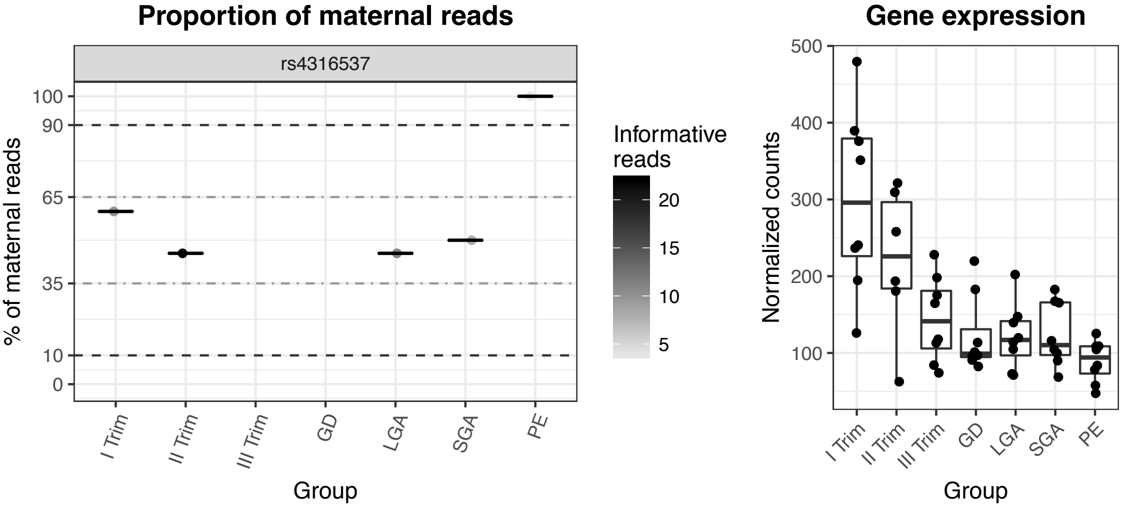

DLG5

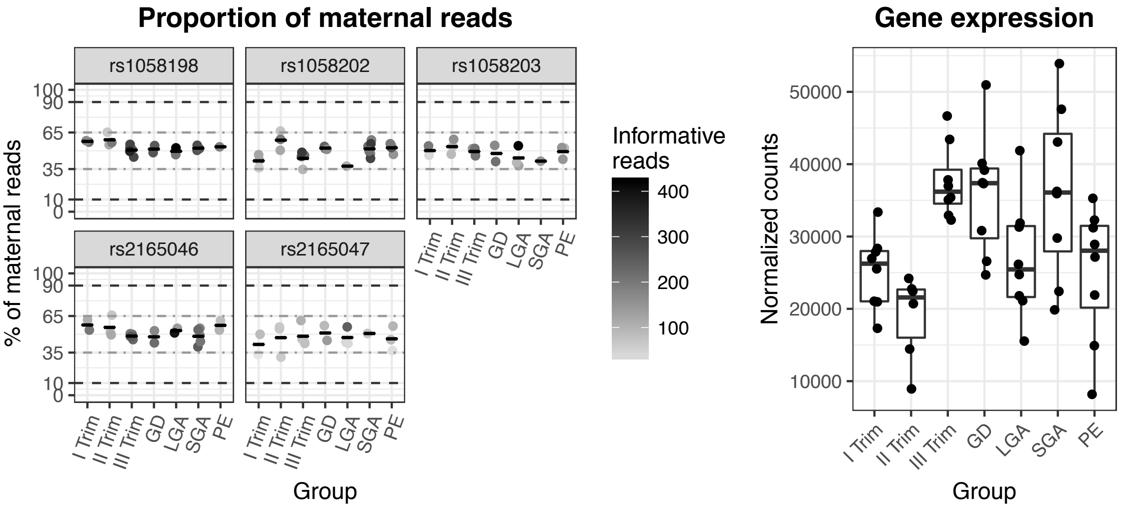

DOCK1

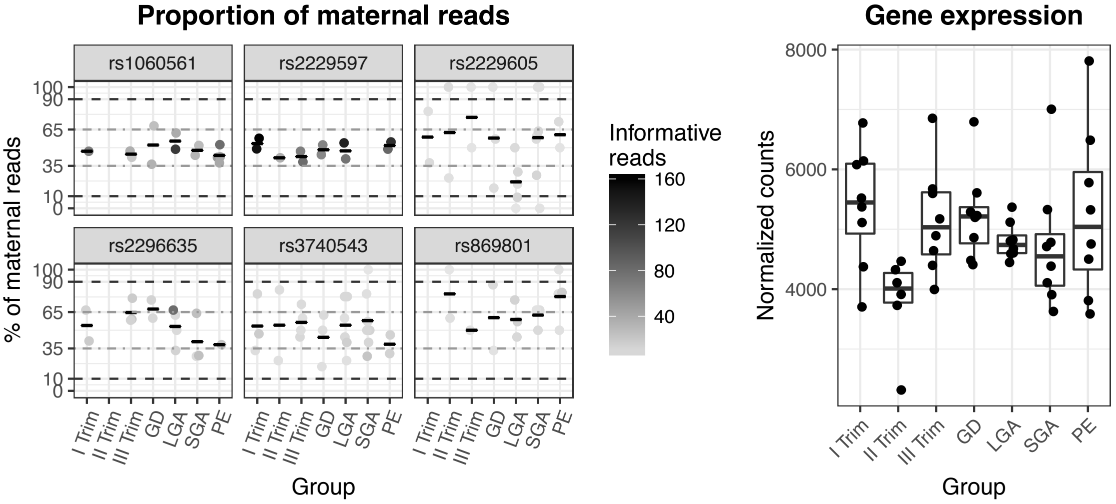

EGFL7

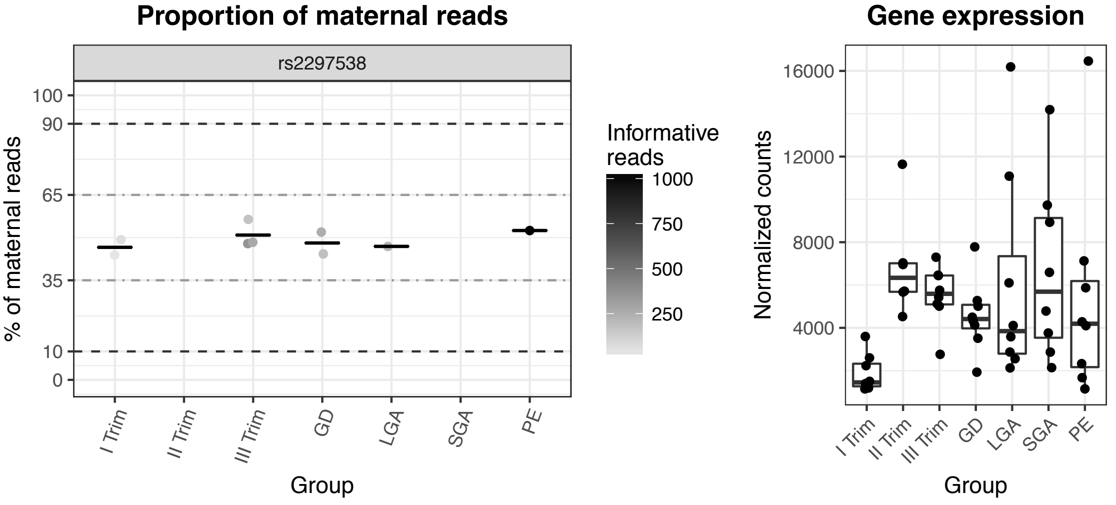

FAM20A

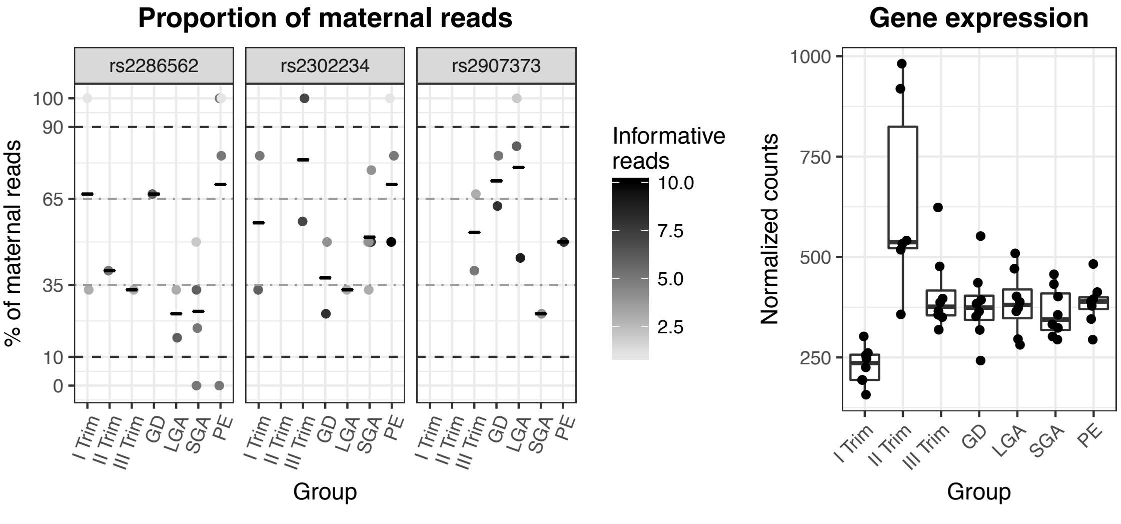

FERMT2

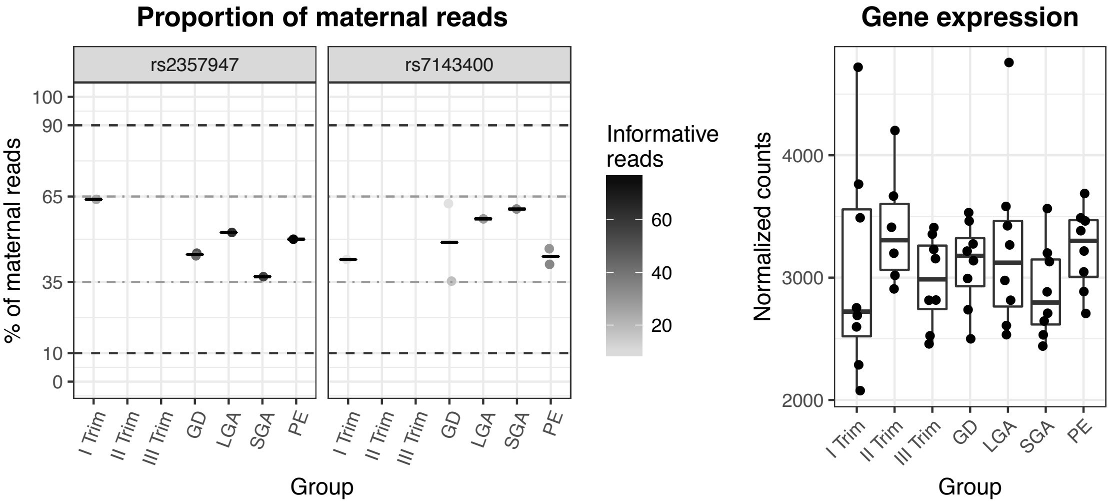

GAREM

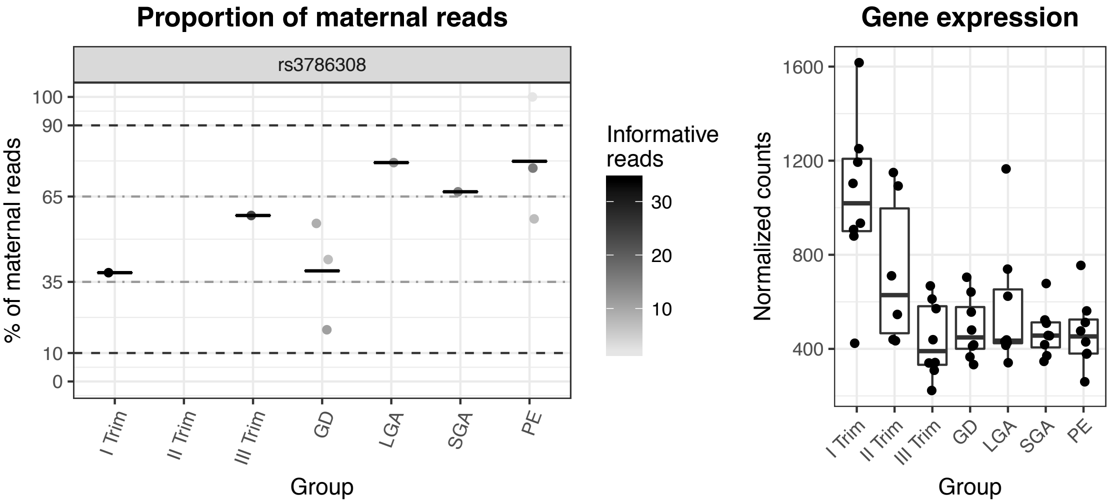

GATA3

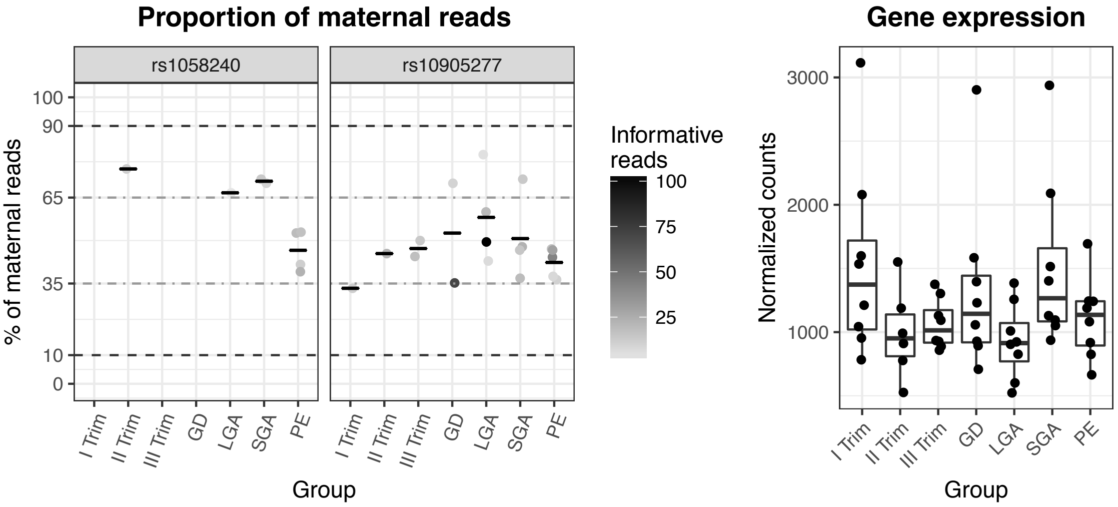

GATM

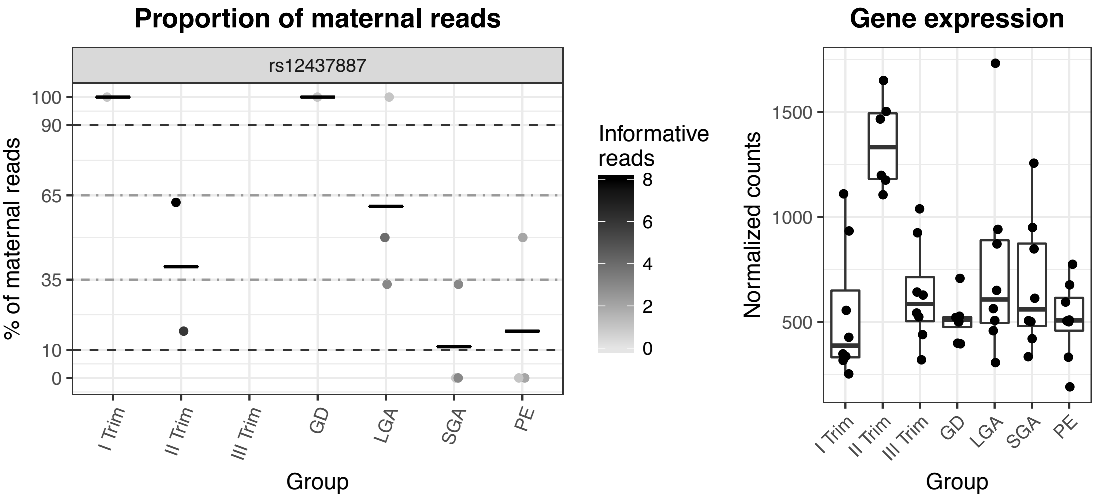

GLI3

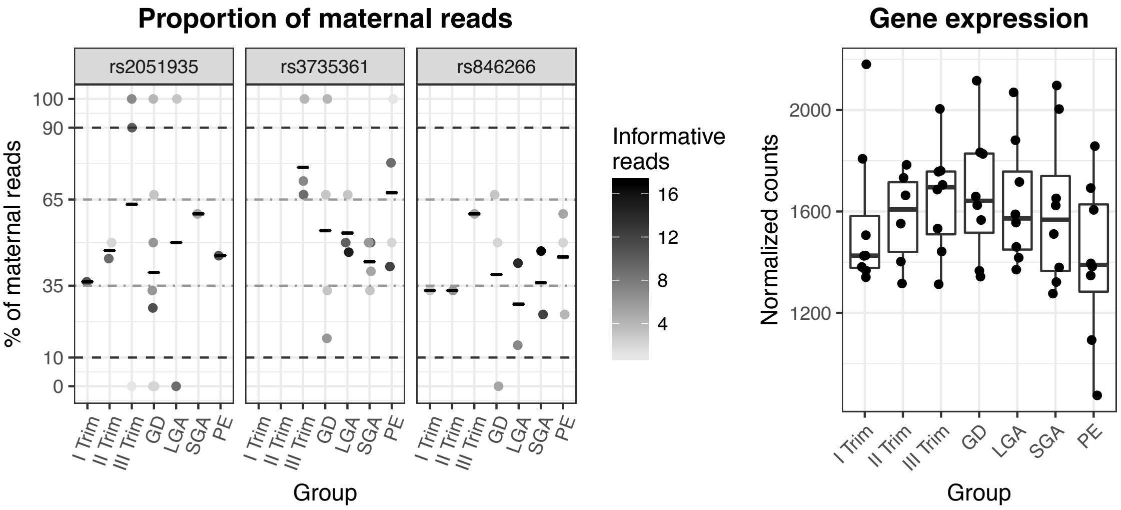

GNAS

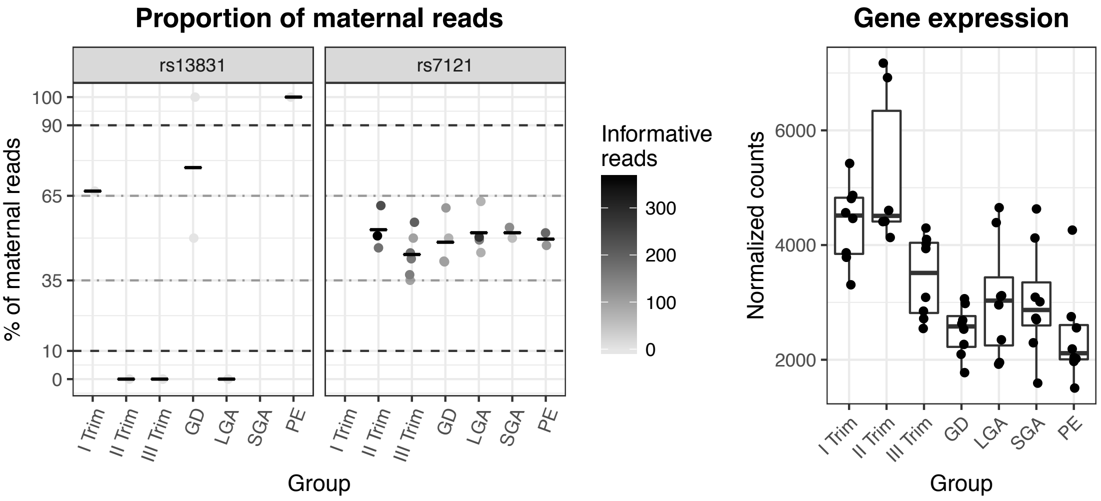

HEG1

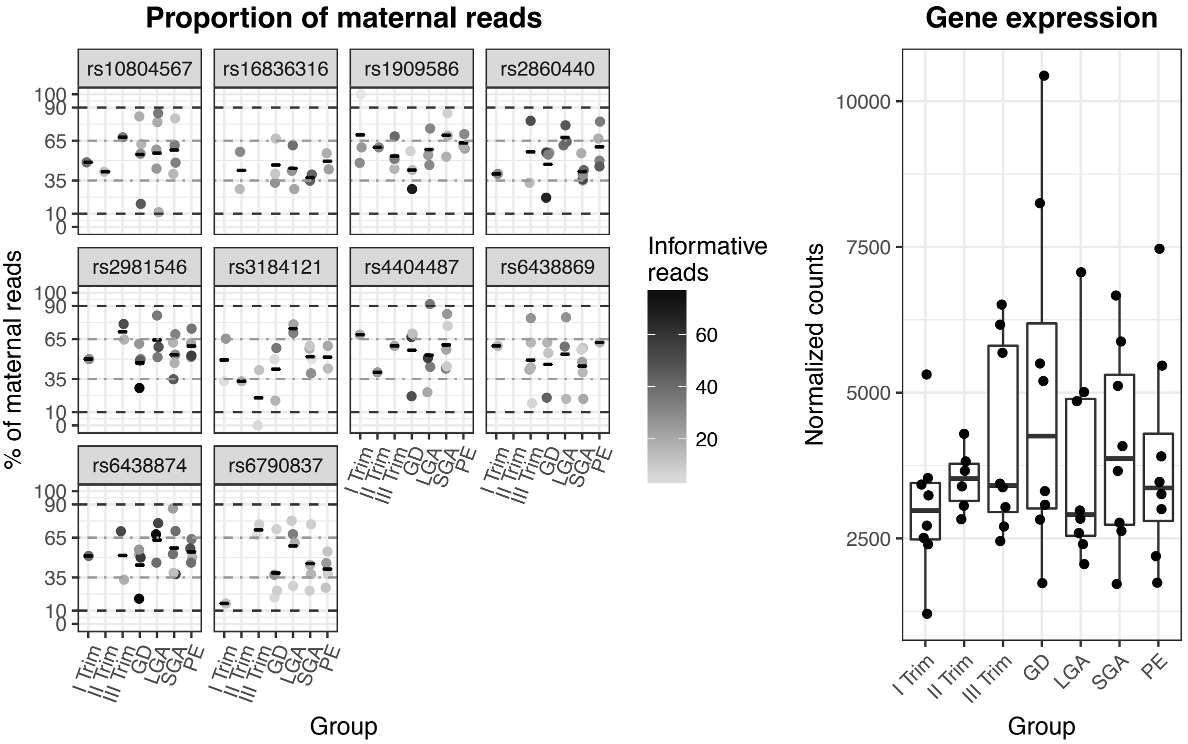

Proportion of maternal reads
rs10804567
rs16836316
rs1909586
rs2860440
rs2981546
rs3184121
rs4404487
rs6438869
rs6438874
rs6790837
% of maternal reads
100
90
65
35
10
0
I Trim
II Trim
III Trim
GD
LGA
SGA
PE
Group
Informative reads
60
40
20
Gene expression
Normalized counts
10000
7500
5000
2500
Group

ICAM1

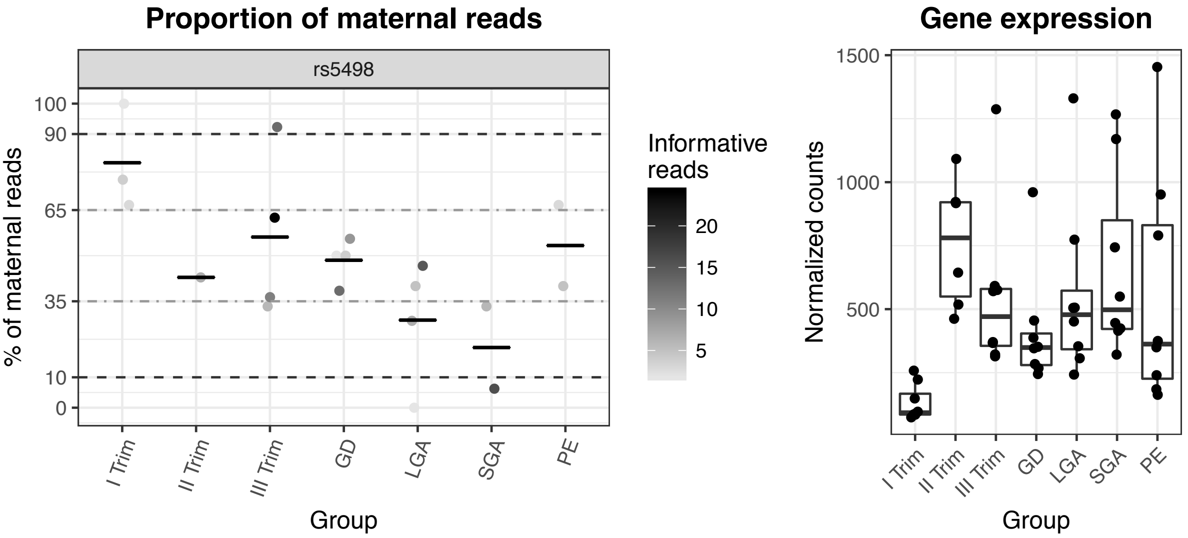

Proportion of maternal reads
rs5498
% of maternal reads
100
90
65
35
10
0
I Trim
II Trim
III Trim
GD
LGA
SGA
PE
Group
Informative reads
20
15
10
5
Gene expression
Normalized counts
1500
1000
500
Group

IGF1R

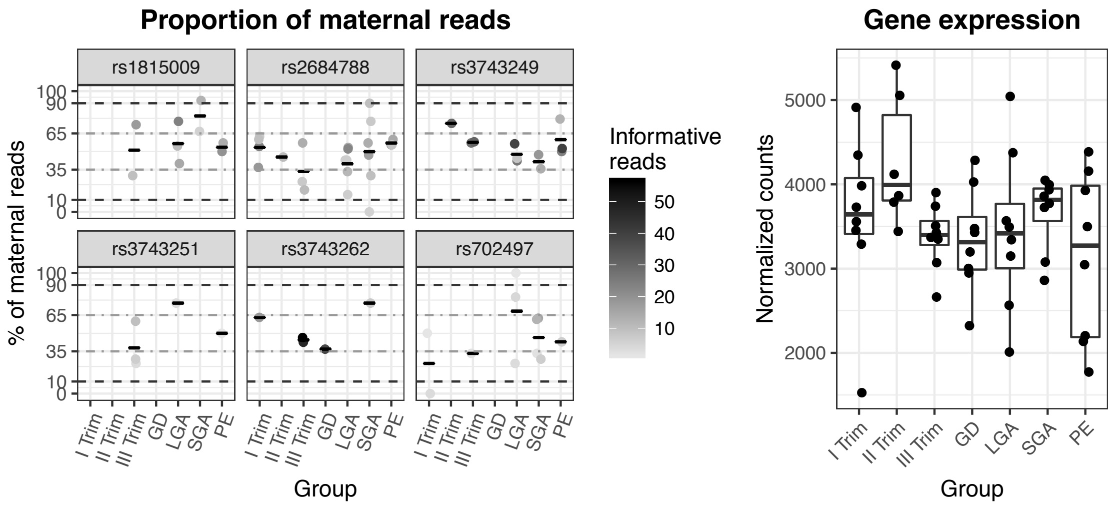

IGF2R

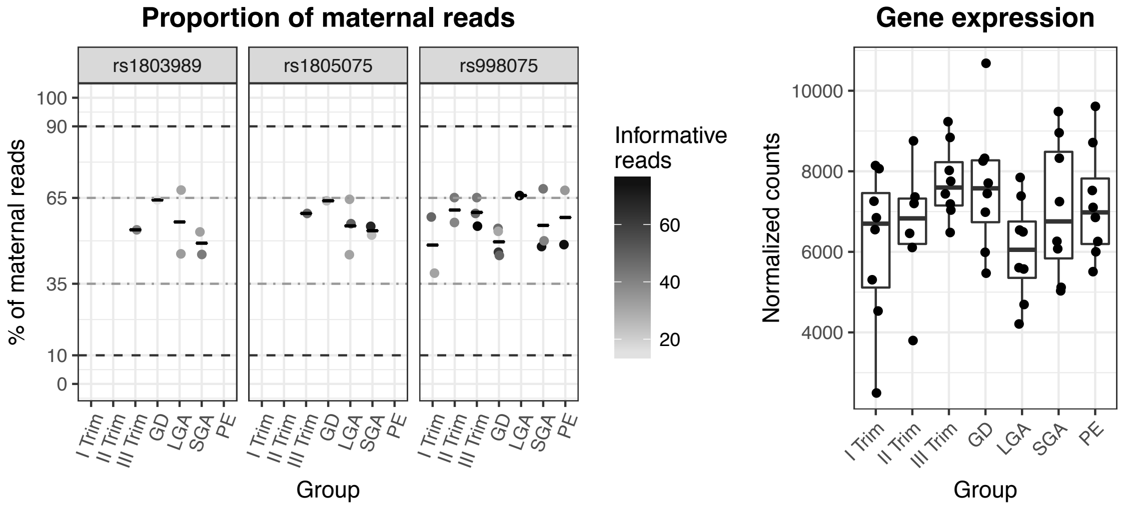

INPP5F

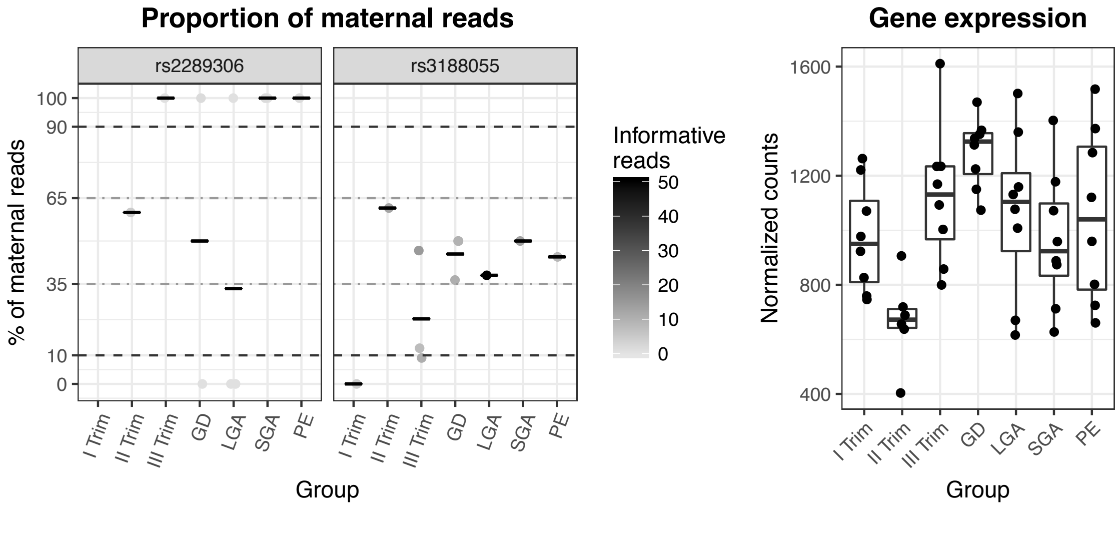

KIAA1191

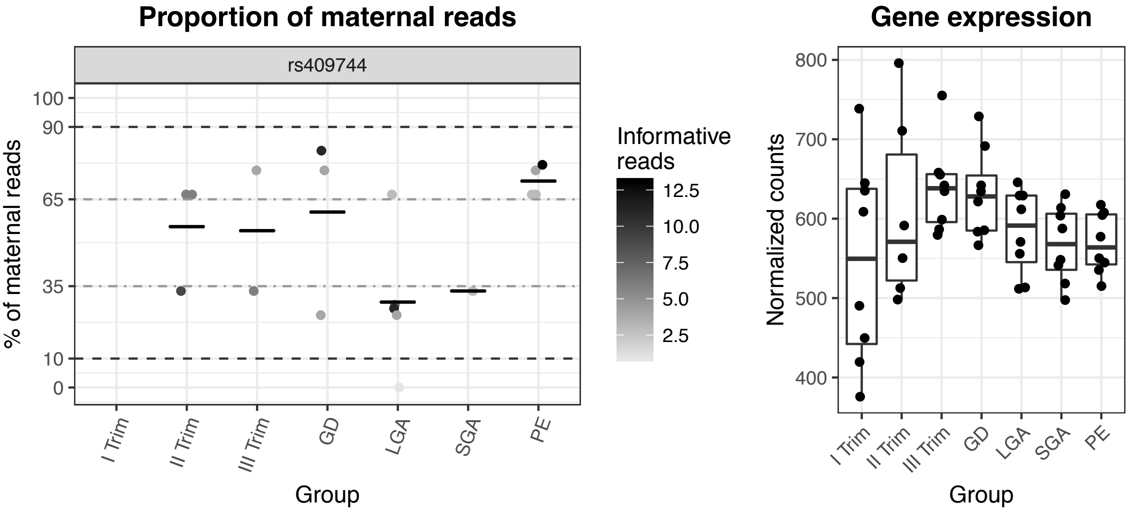

KIAA1551

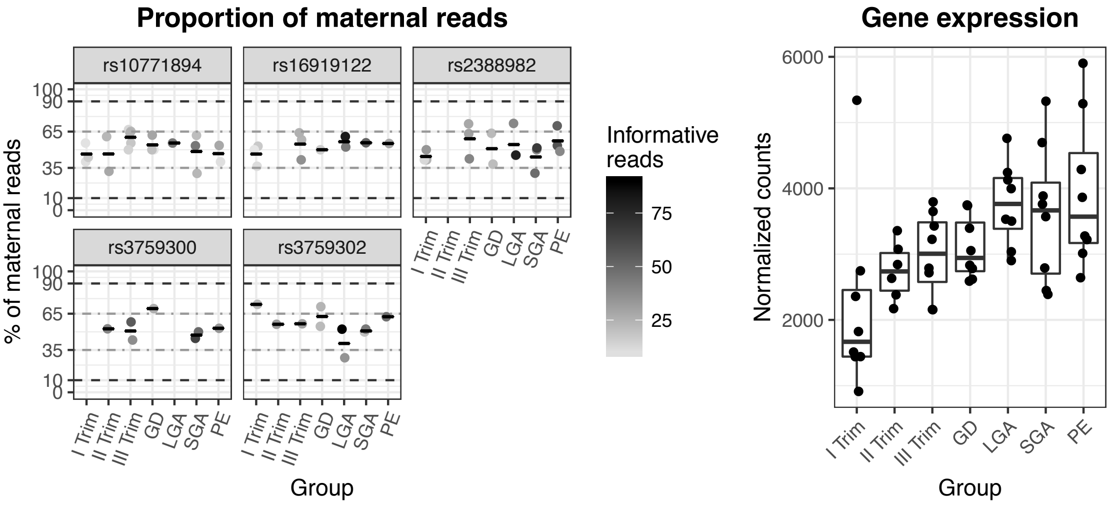

KIAA1919

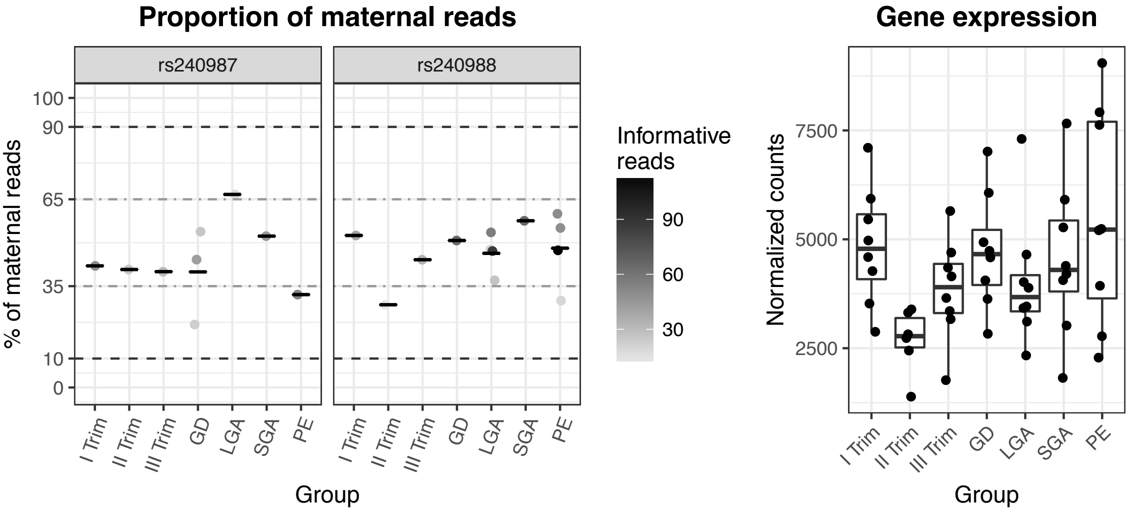

L3MBTL1

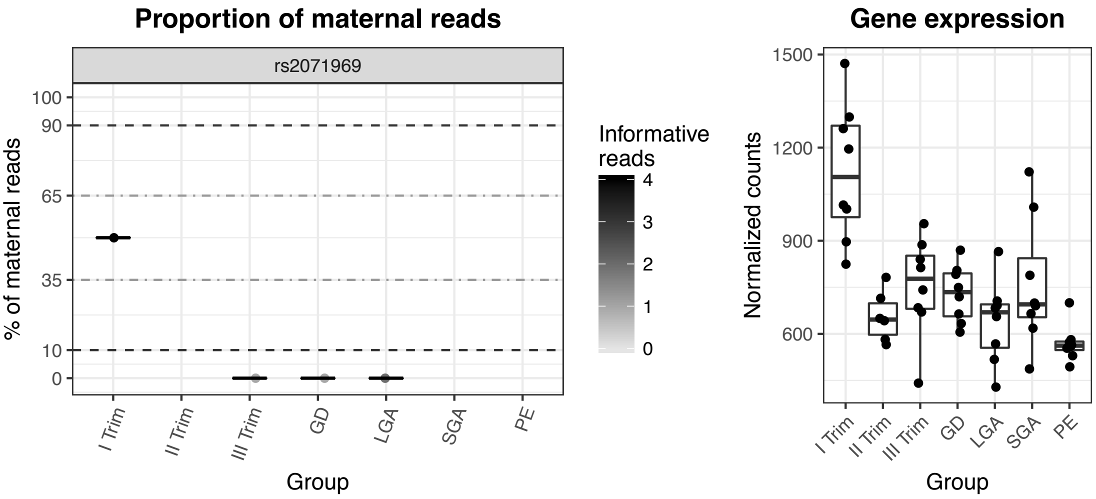

LGALS8

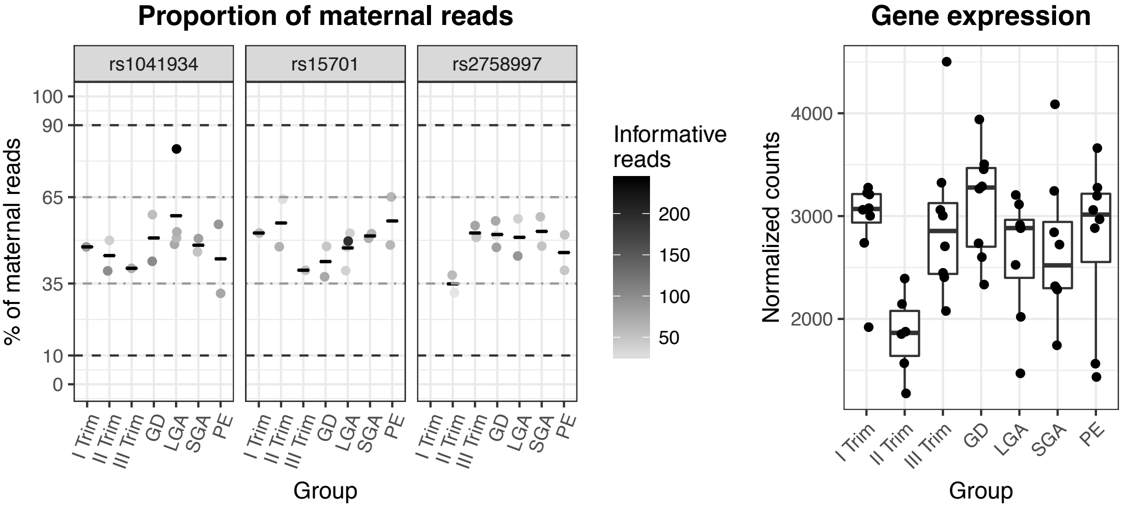

LGALS14

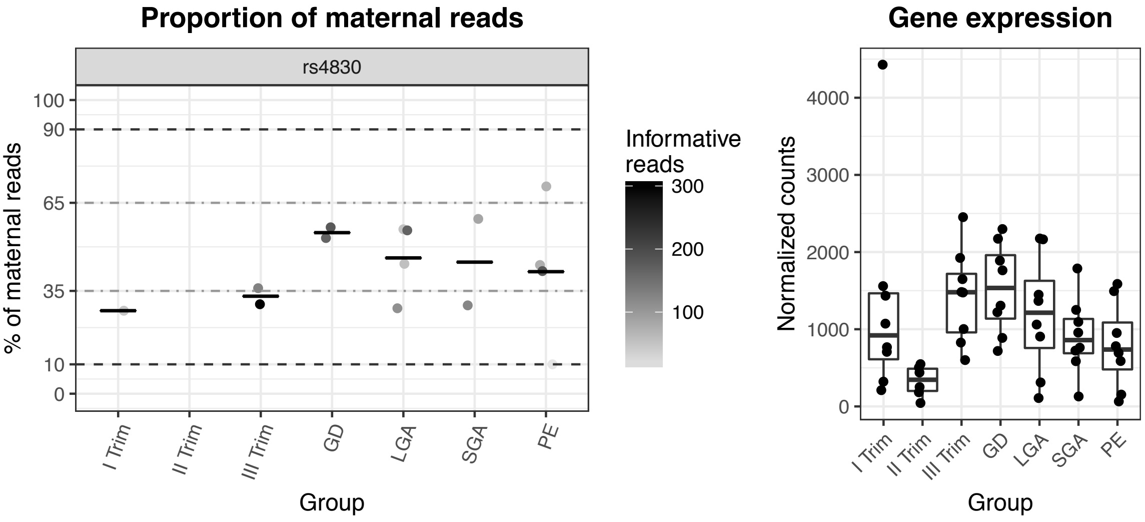

OBSCN

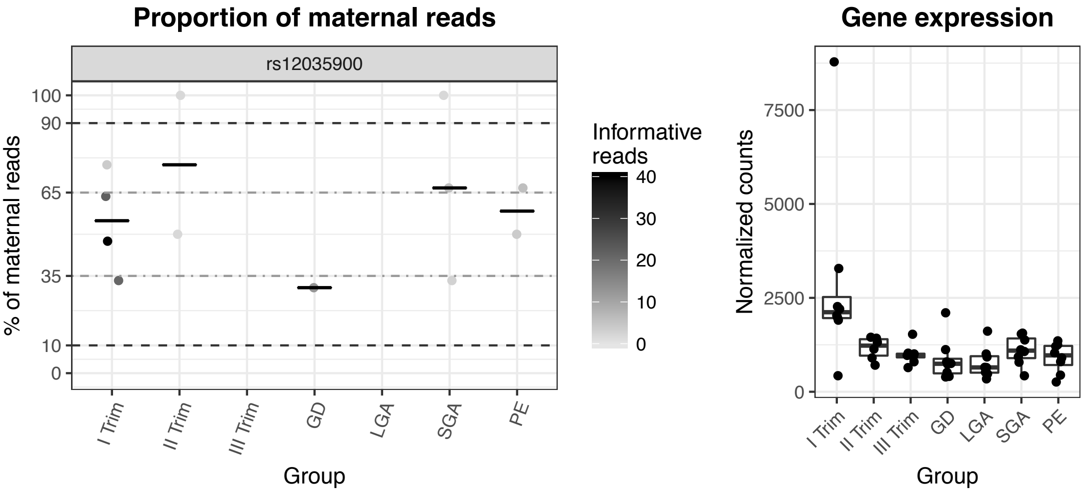

OVCH2

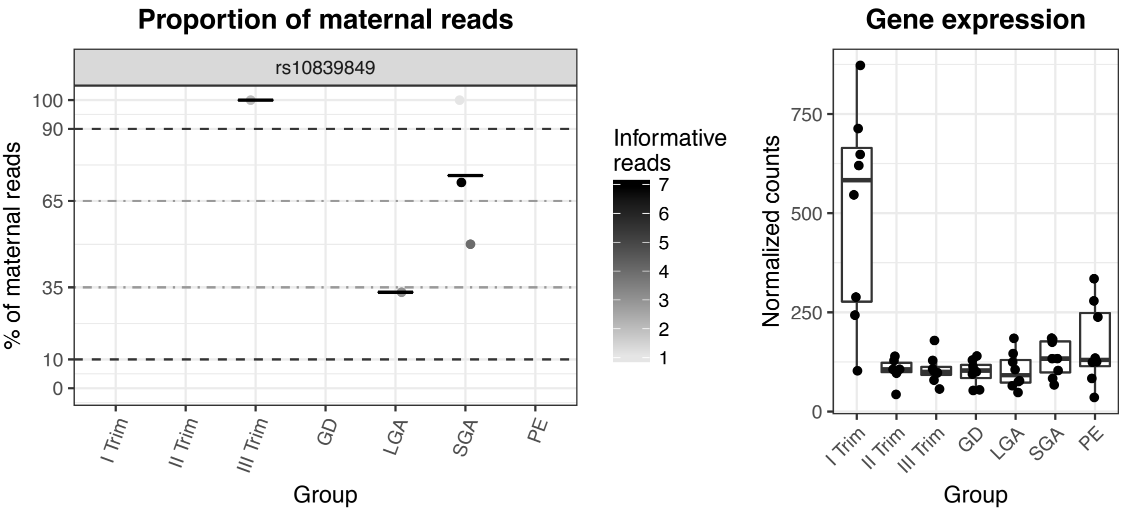

PAPPA2

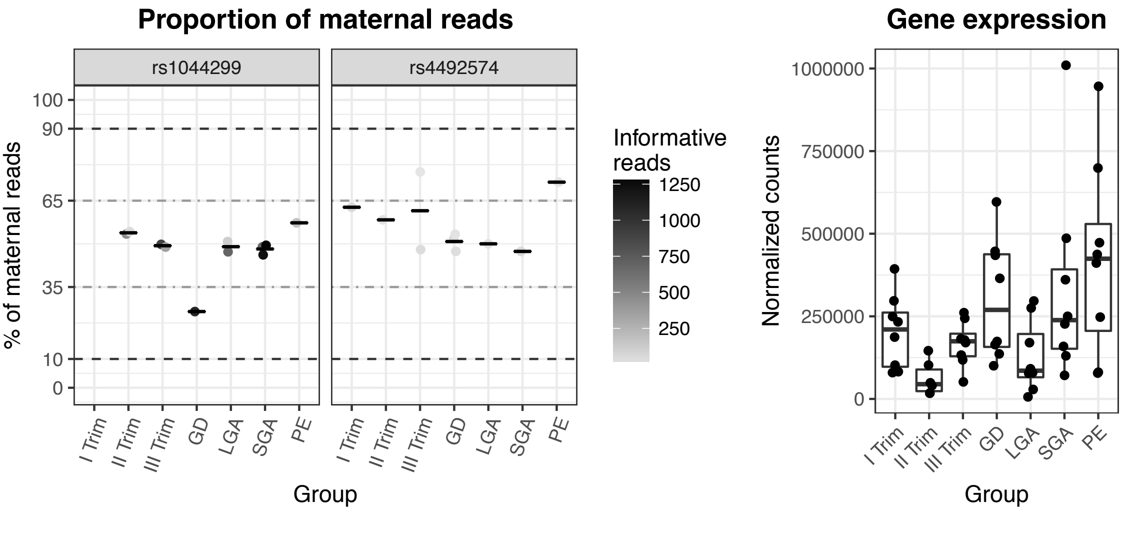

PDE4D

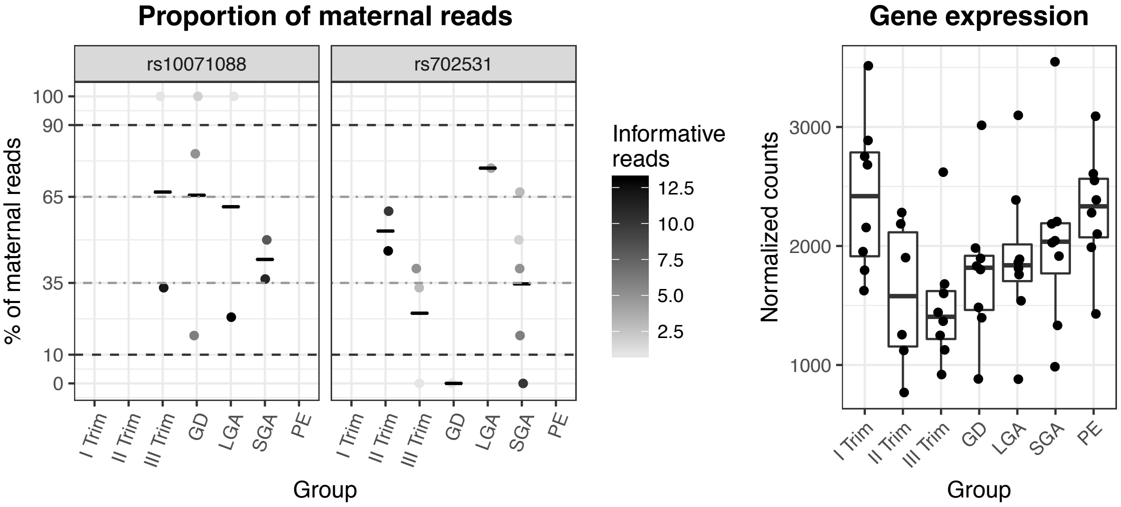

PDK4

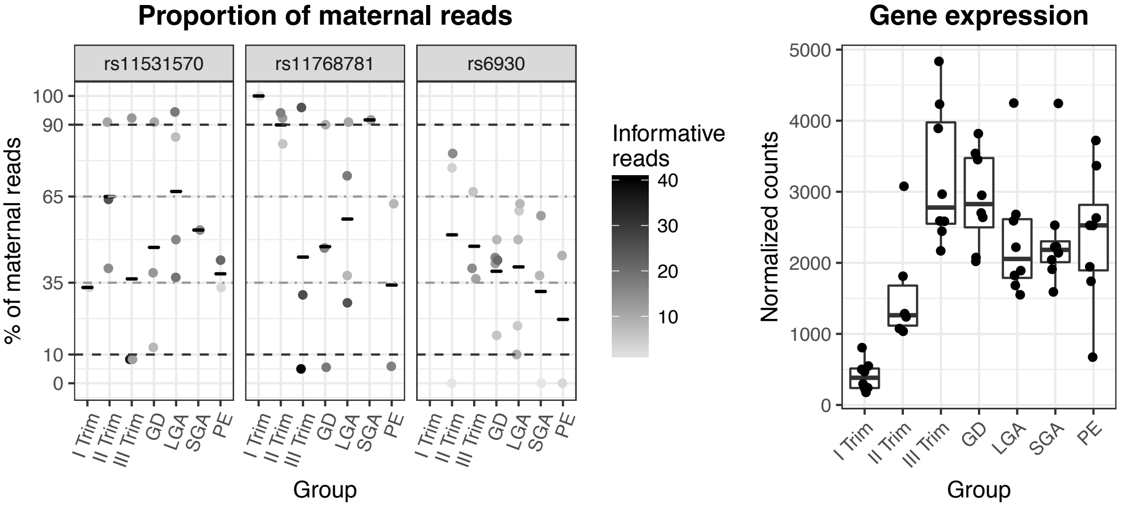

PDPR

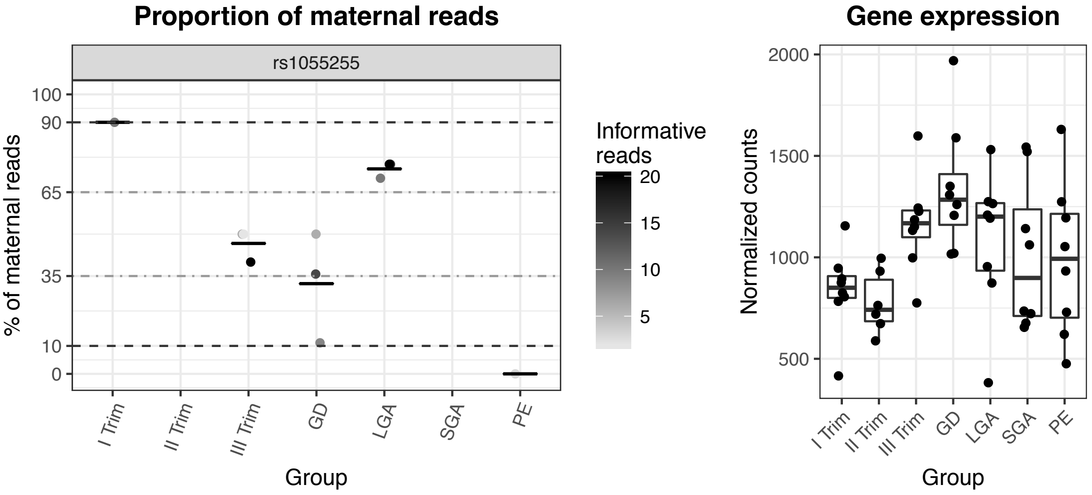

PKP3

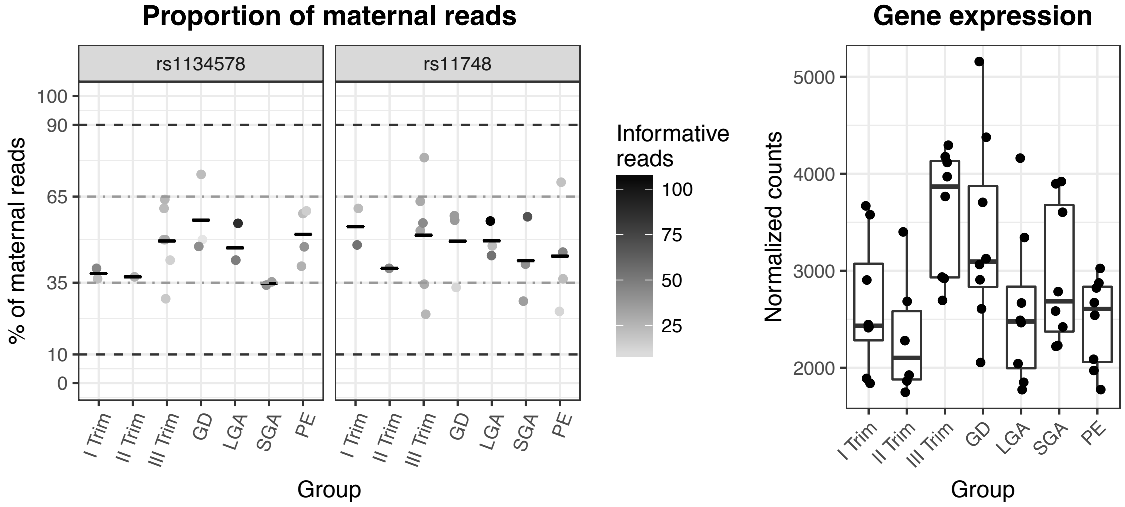

PPP1R9A

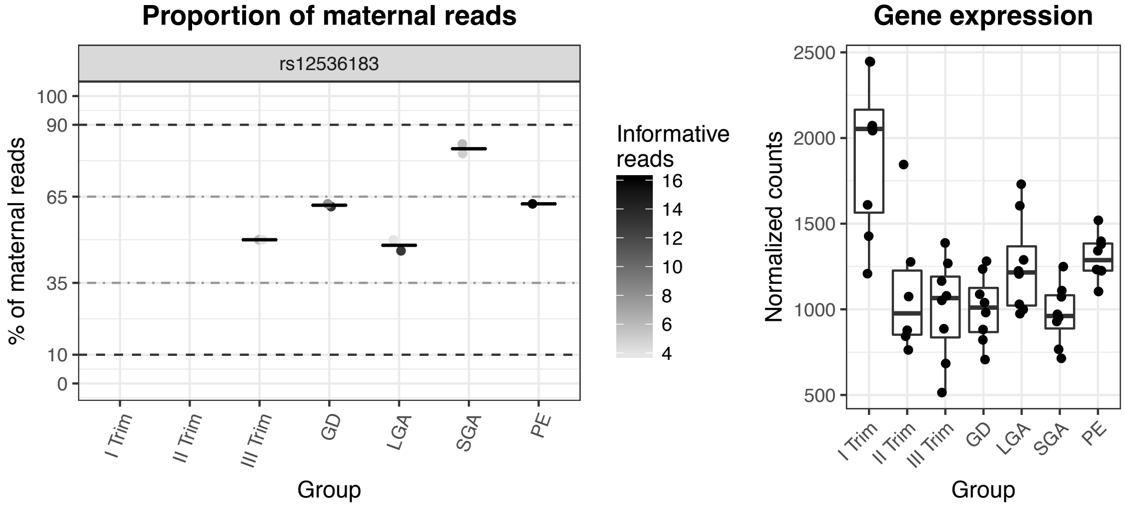

PRKAG2

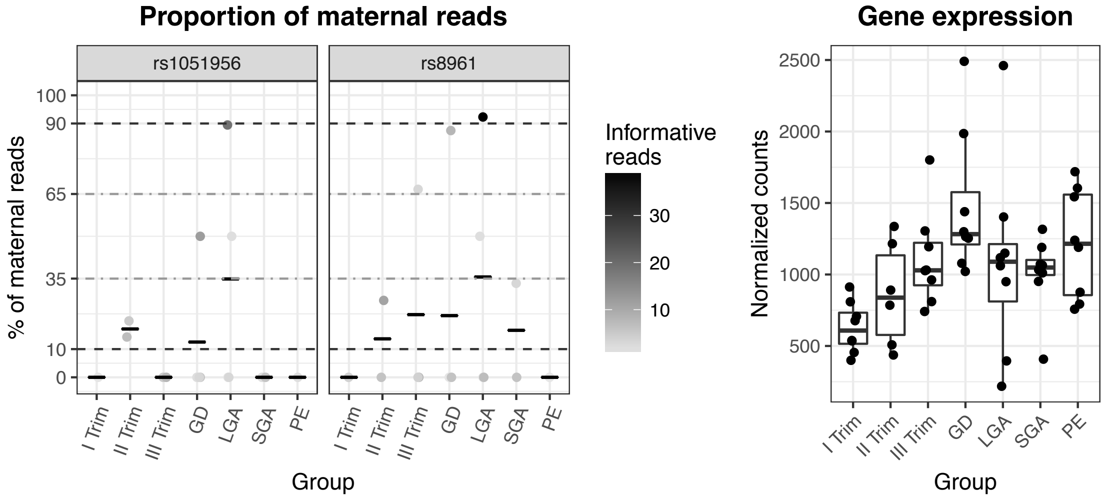

PROSER2–AS1

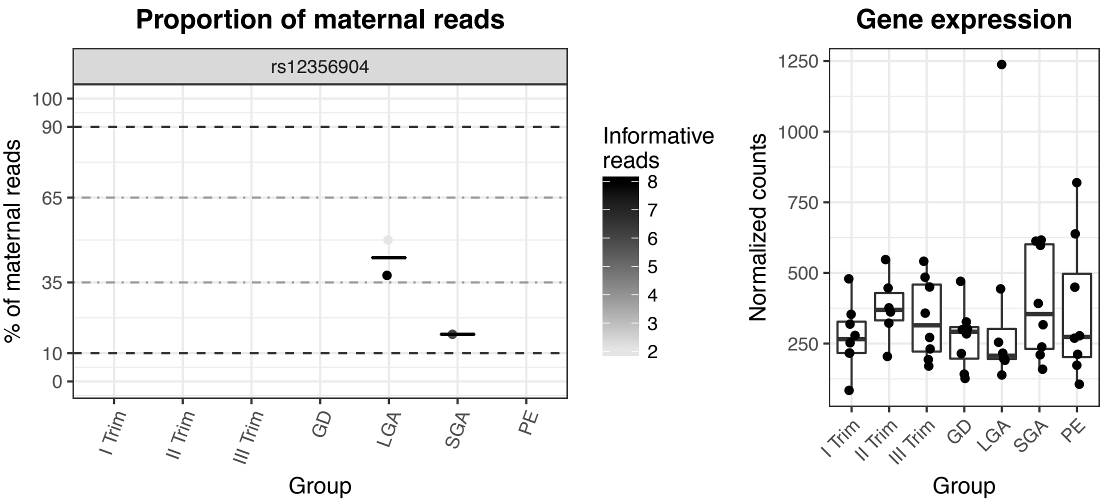

PSCA

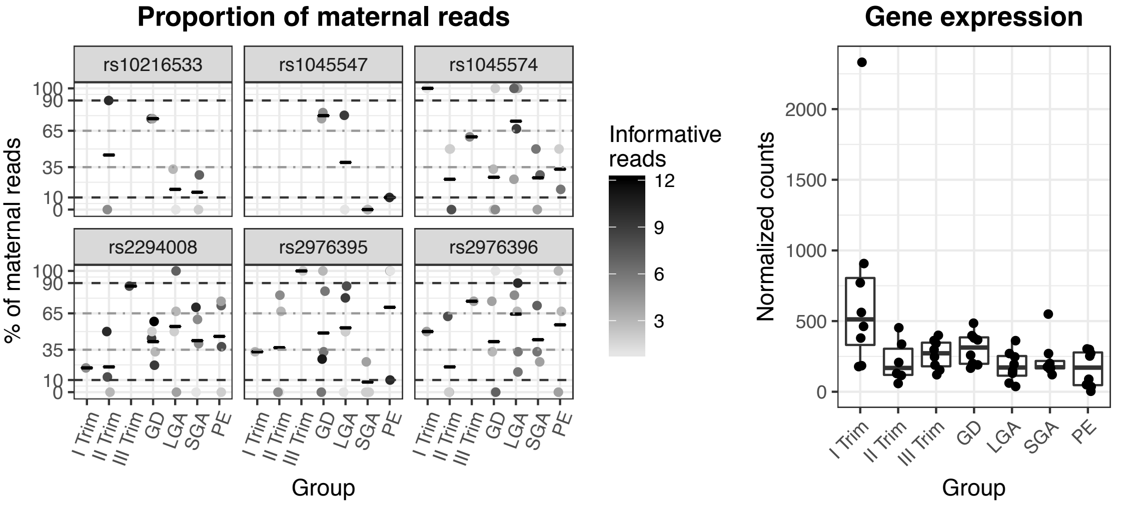

PTGFRN

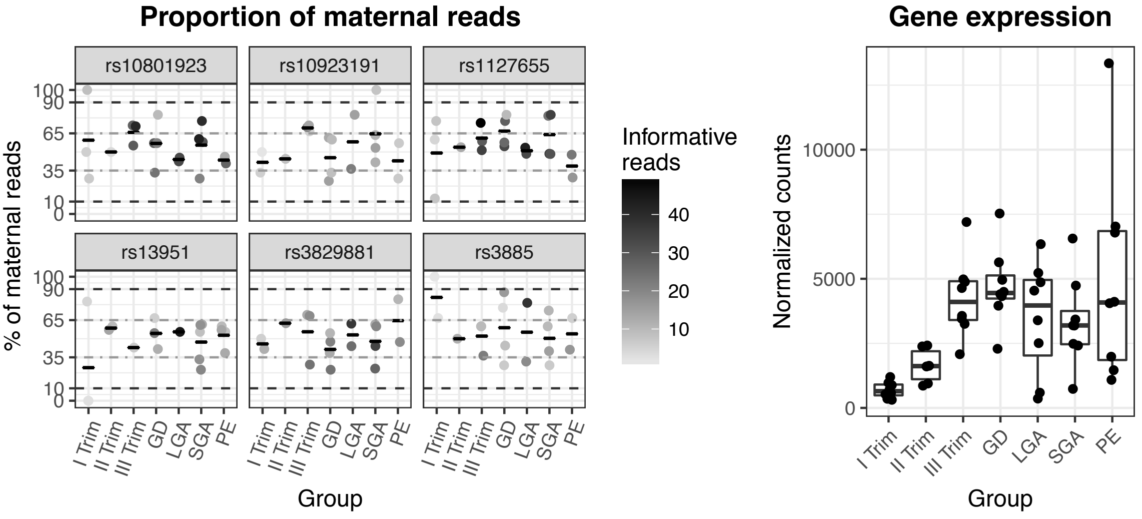

PTPN14

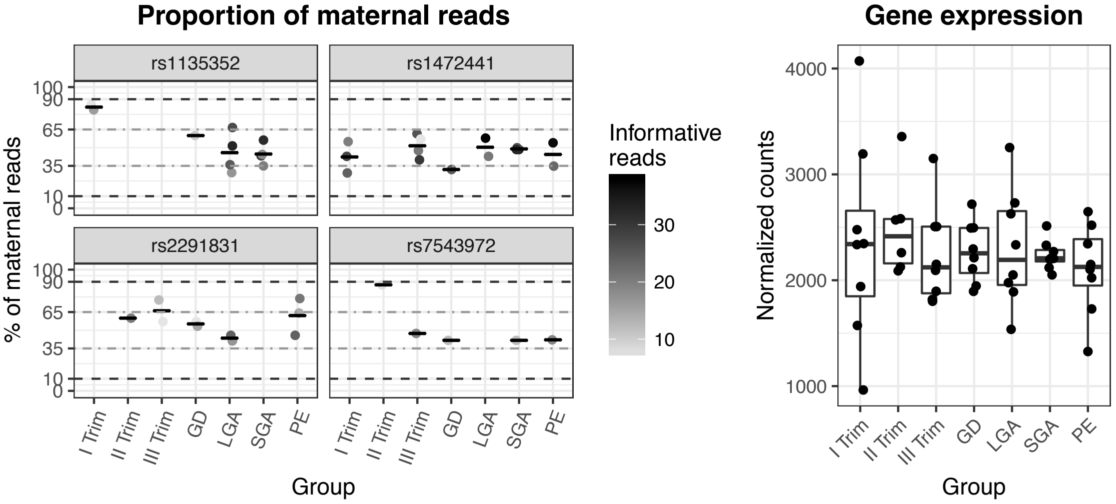

R3HCC1

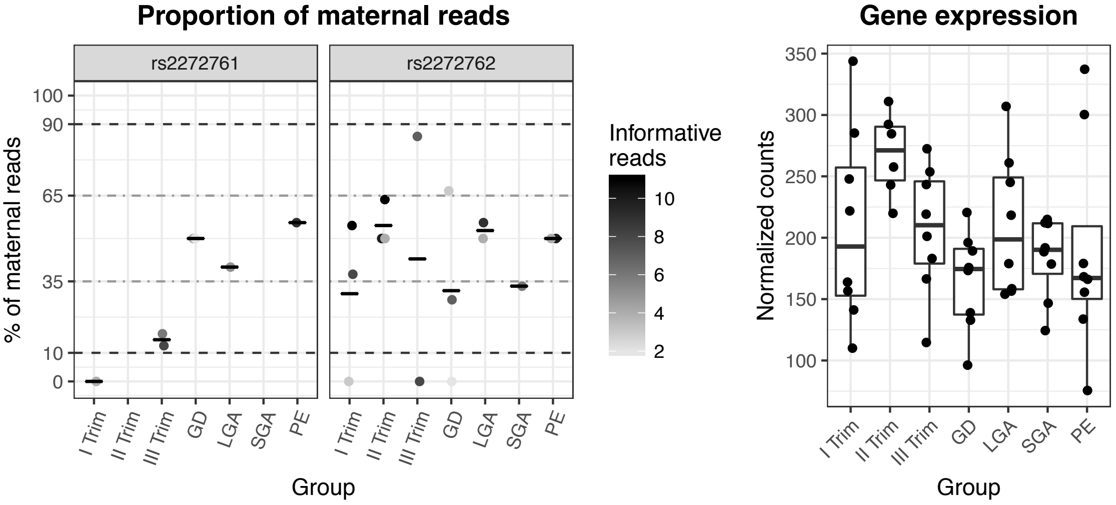

RNF141

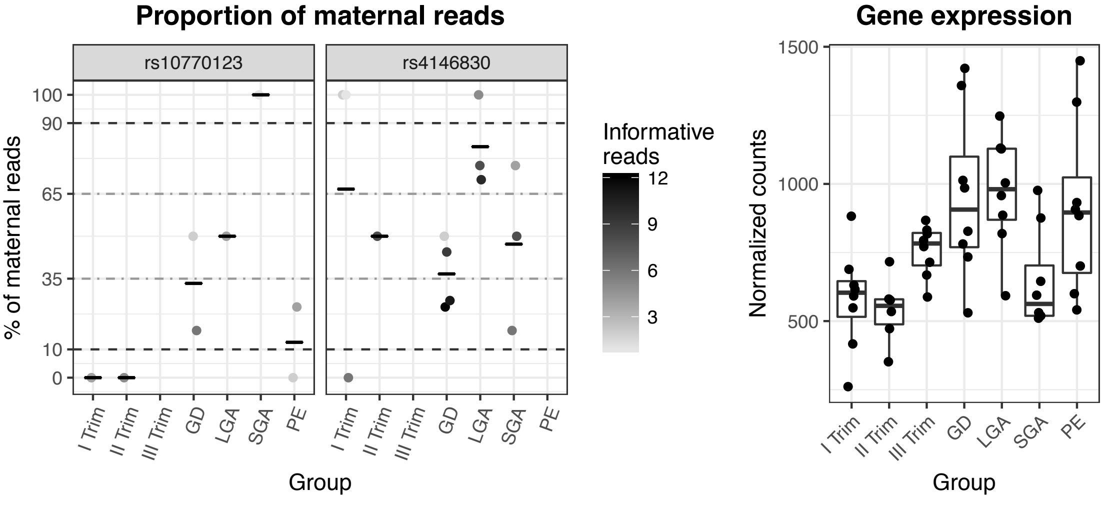

SEC16A

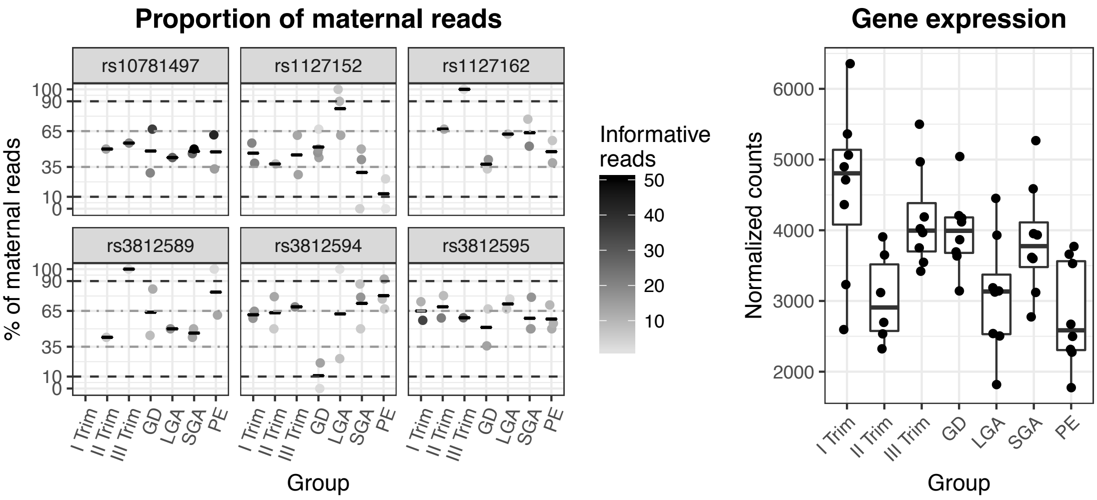

SLC4A2

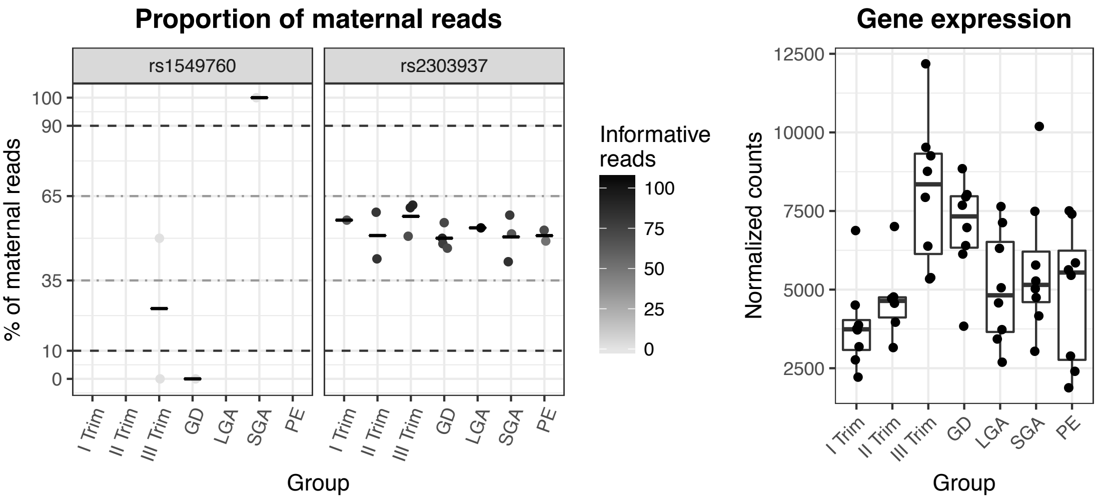

SLC4A7

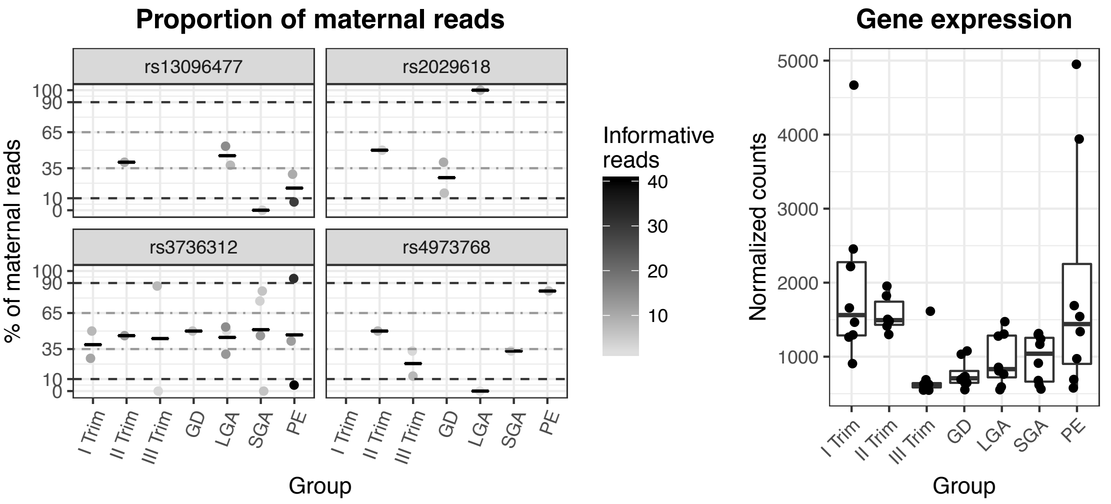

SLC22A3

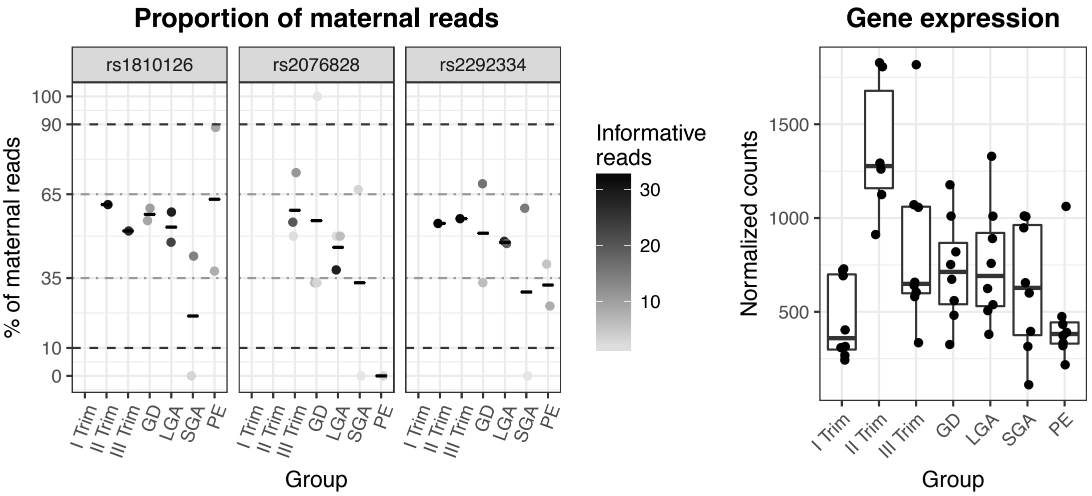

SPON2

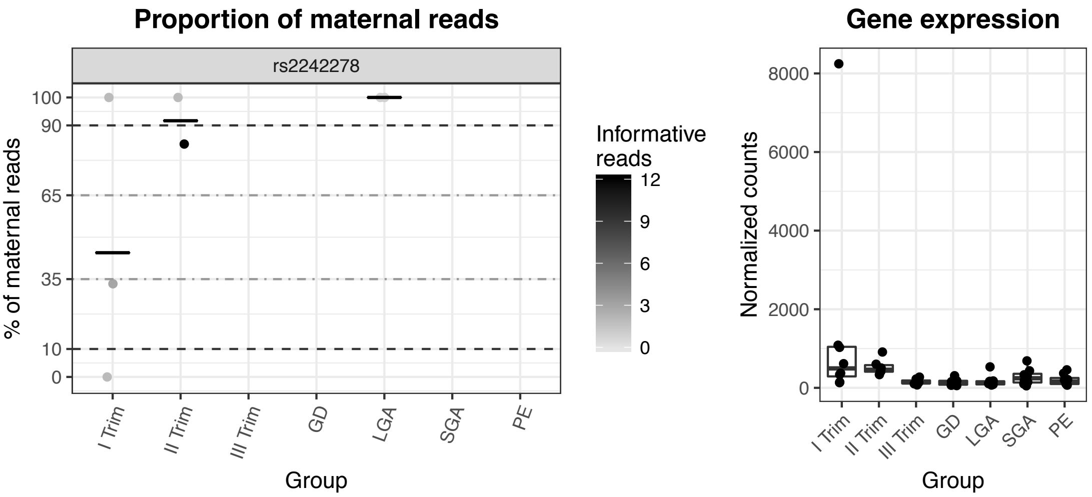

SPTLC3

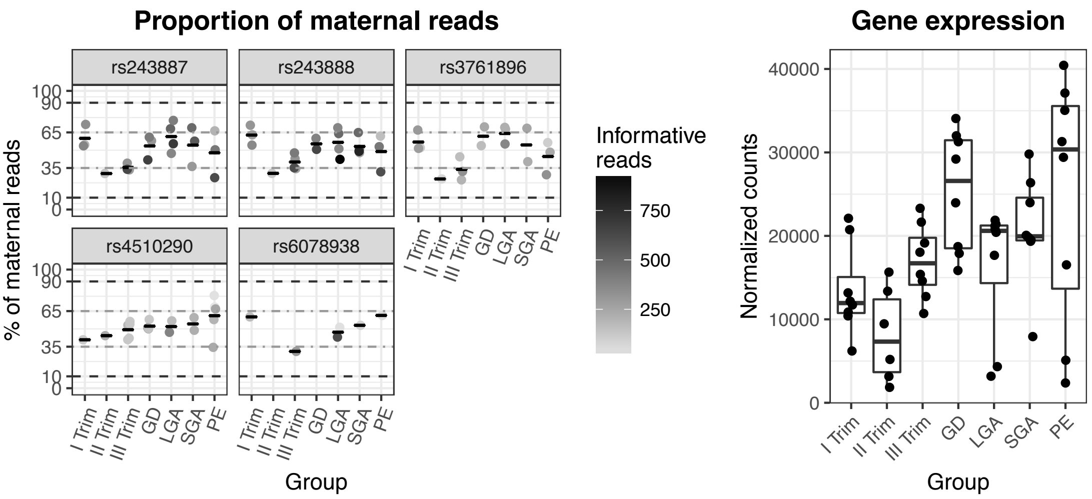

SYNE2

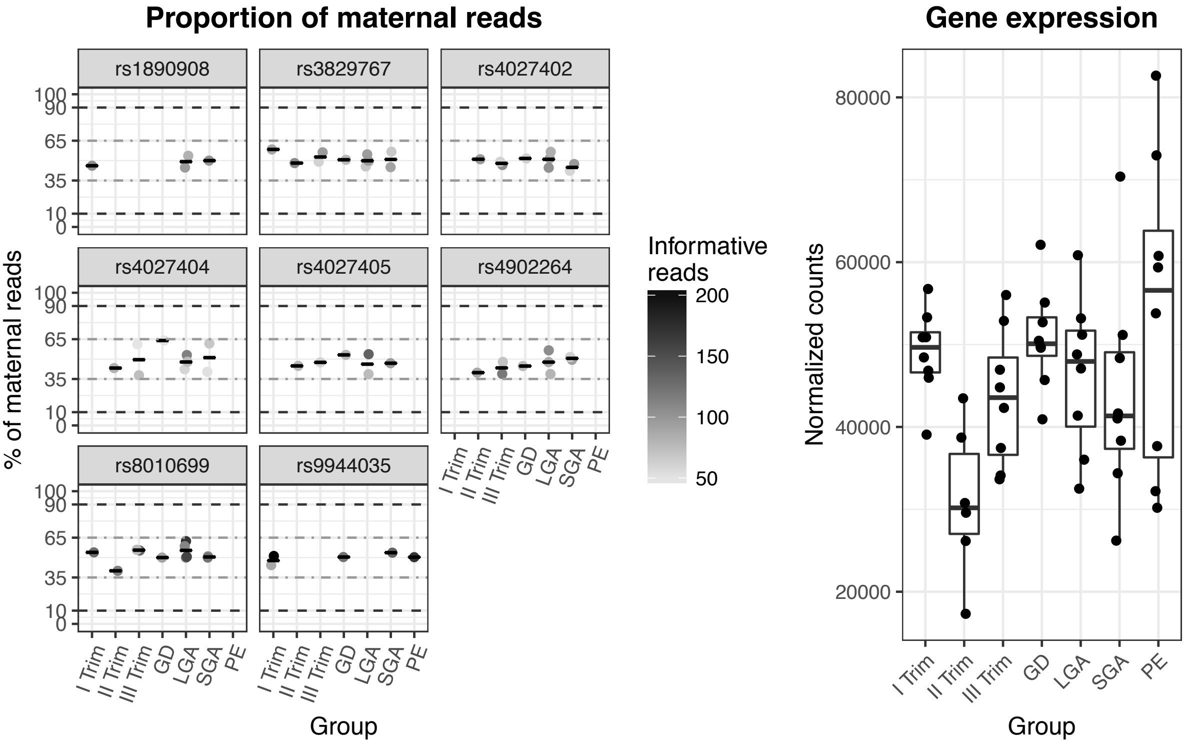

THSD7A

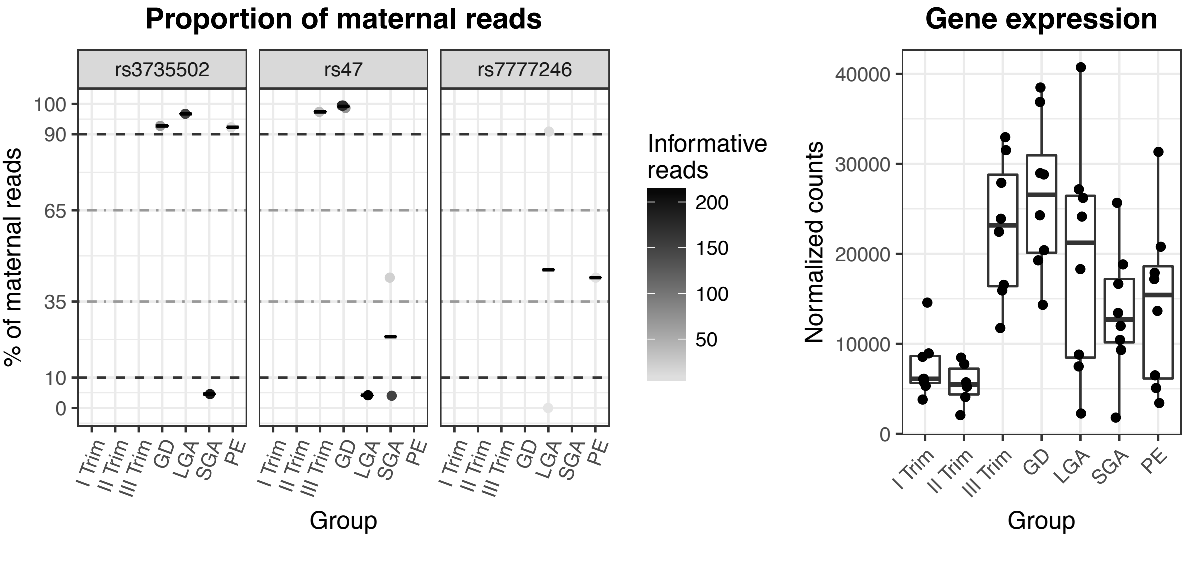

TIGAR

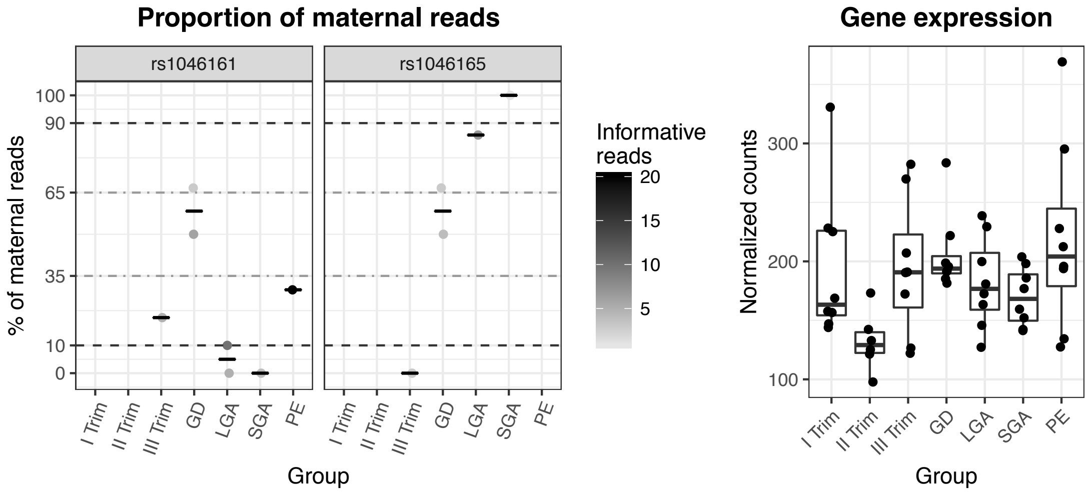

TLR3

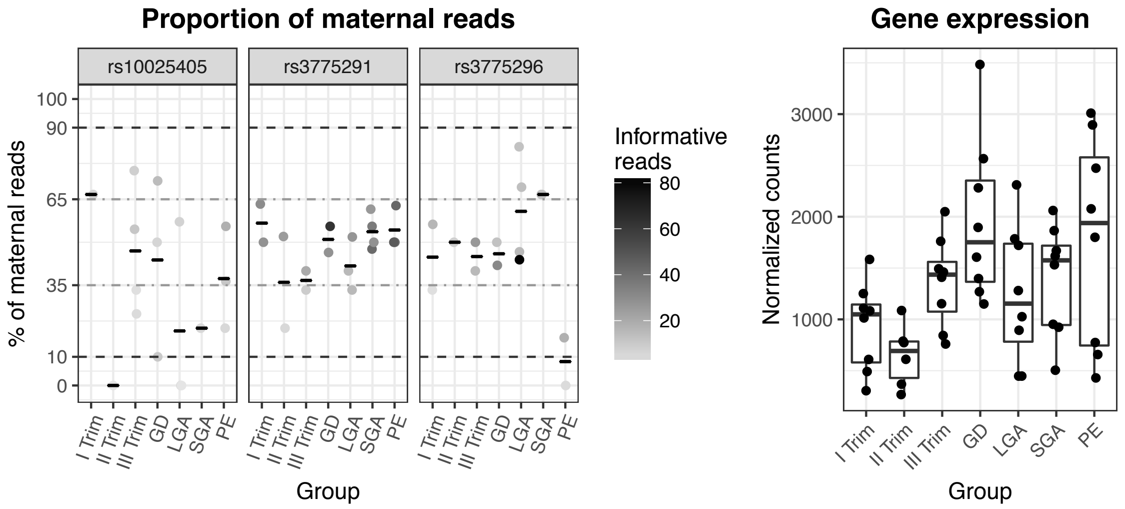

TMEM60

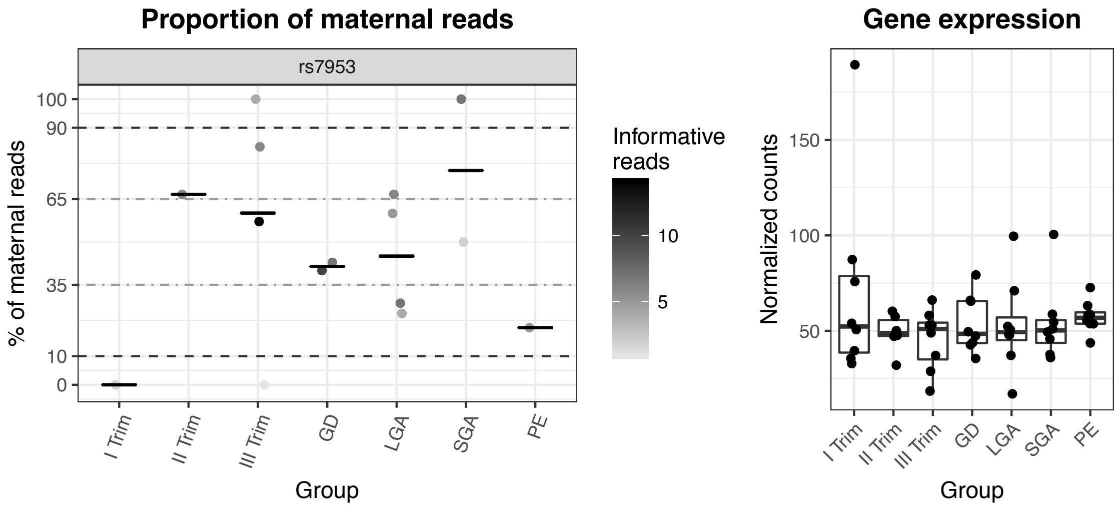

TP53

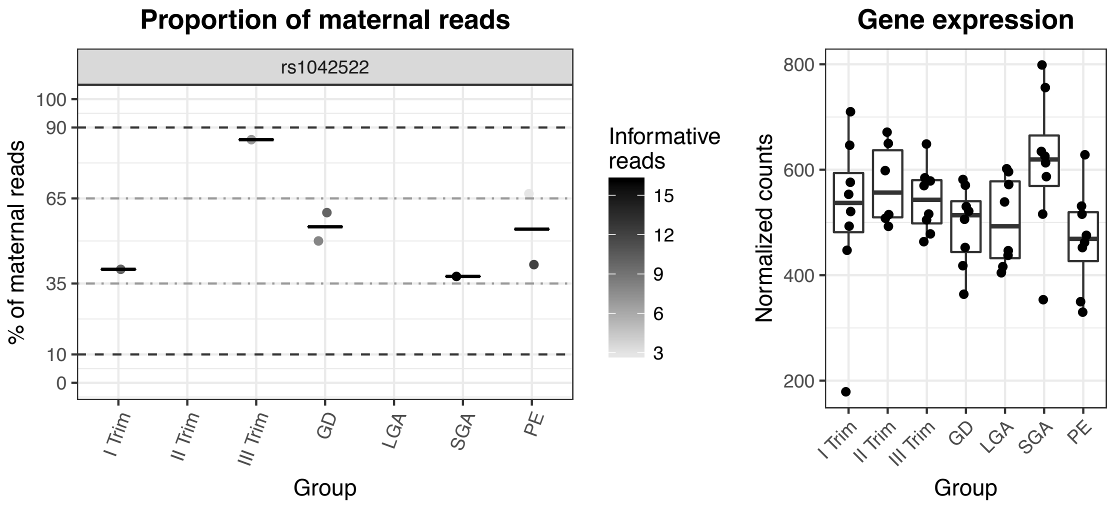

TRAPPC9

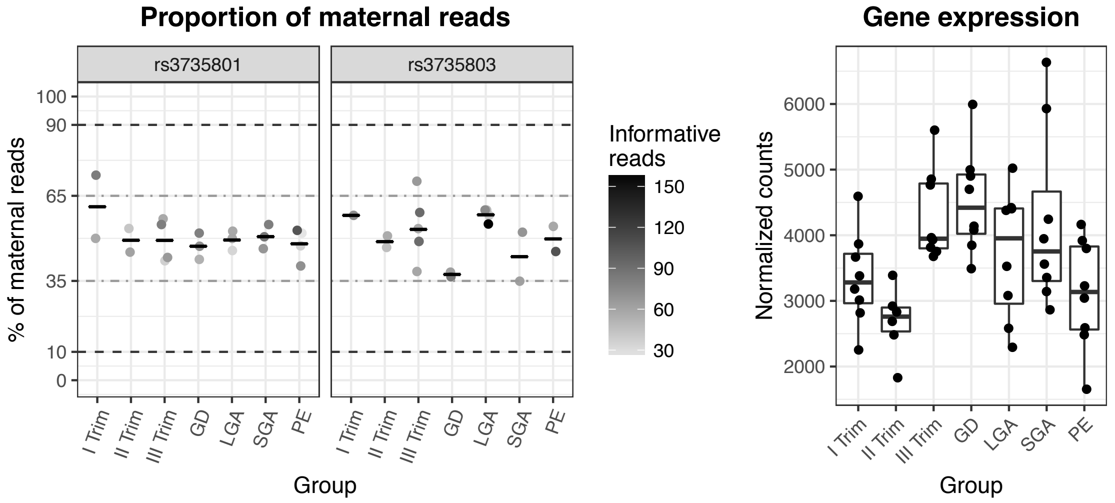

WDR27

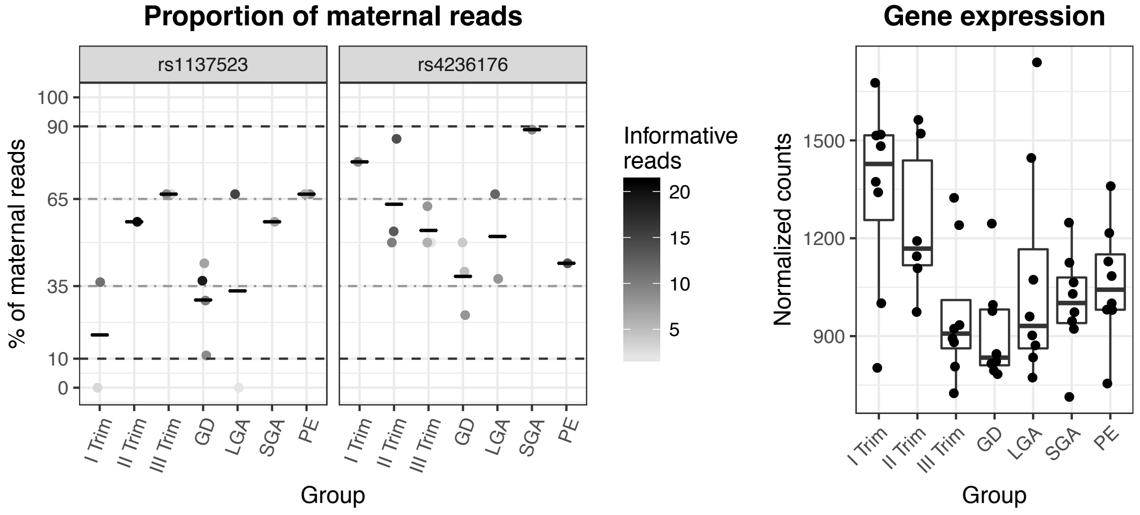

ZNF264

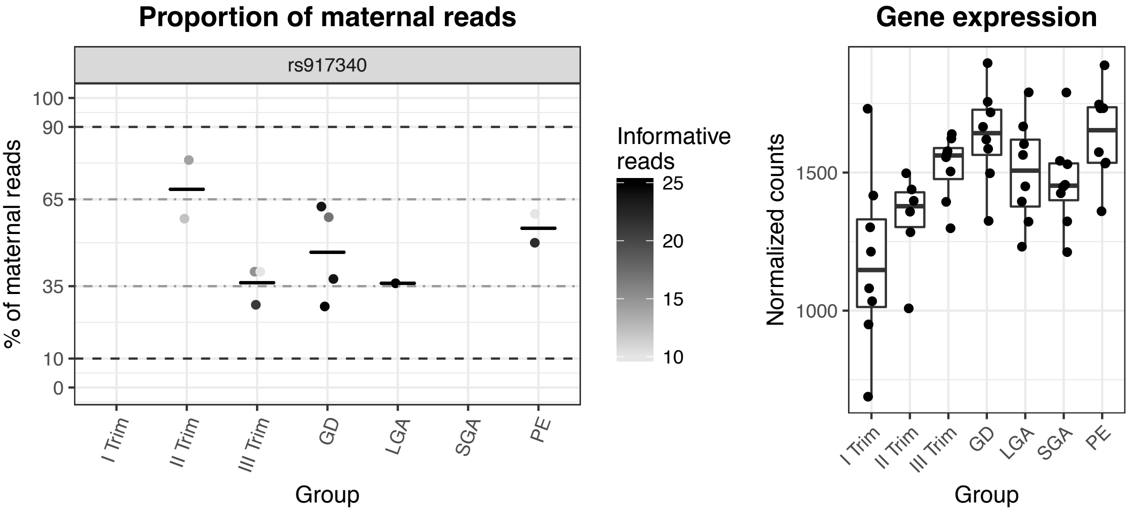

ZNF331

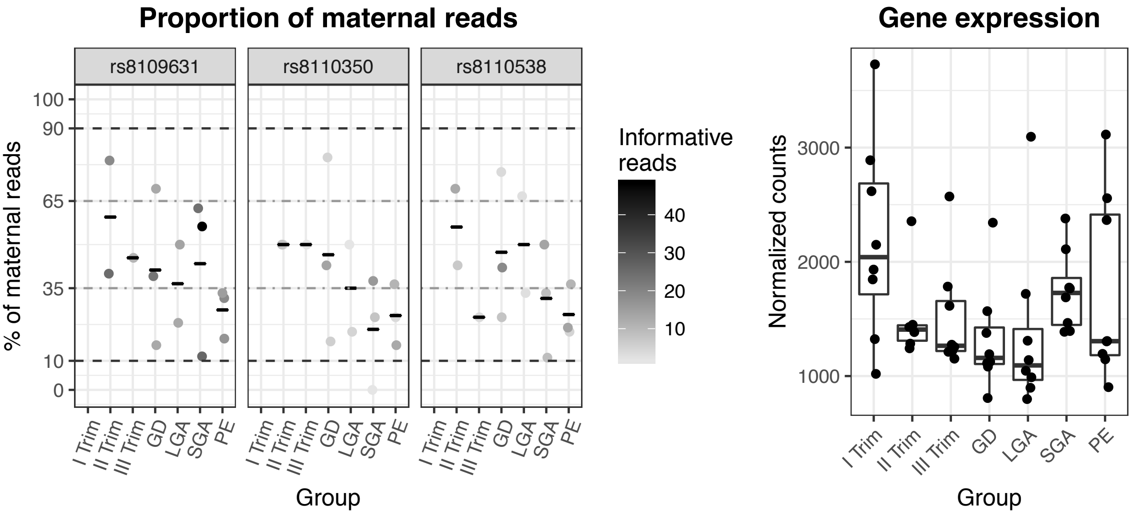

Supplement: Supplementary file 9 — Figure S2. Catalog of the parental allelic proportions and gene expression level of all analyzed 91 candidate imprinted genes across gestation (first, second, and third trimester normal pregnancy) and in cases of term pregnancy complications (preeclampsia, gestational diabetes, delivery of a small- or large-for-gestational-age newborn). (PDF 2660 kb) [file 13148_2019_692_MOESM9_ESM.pdf]
