# Supplementary material for: Parent-of-origin-specific allelic expression in the human placenta is limited to established imprinted loci and it is stably maintained across pregnancy
Source: Clin Epigenetics. 2019 Jun 26;11:94. doi: 10.1186/s13148-019-0692-3 (PMC6595585; doi:10.1186/s13148-019-0692-3)
Supplement: Supplementary file 10 — Table S7. Experimental validation of parent-of-origin-specific or biallelic expression of selected genes using RT-PCR, cloning, and sequencing. (PDF 68 kb) [file 13148_2019_692_MOESM10_ESM.pdf]

Table S7. Experimental validation of parent-of-origin specific or biallelic expression of selected genes using RT-PCR, cloning and sequencing.

		Placental	Genotypes			No of clones		Allele %		Summary	Placental expression	
Gene	SNP ID	sample	Pat	Mat	PL	Mat	Pat	Mat	Pat	Mat/Pat	RT-PCR	RNA-Seq
RTL1	rs3825569	I	GG	AG	AG	13	0	100	0	50/1	Maternal	Maternal
		II	AG	GG	AG	20	0	100	0	(98.0%)		
		III	AA	GG	AG	17	1	94	6			
	rs6575805	I	GG	AG	AG	11	0	100	0	30/1	Maternal	Maternal
		II	AG	GG	AG	13	0	100	0	(96.8%)		
		III	AG	GG	AG	6	1	86	14			
DLK1	rs1802710	I	CC	TT	CT	1	9	10	90	2/30	Paternal	Paternal
		II	TT	CC	CT	1	9	10	90	(6.25%)		
		III	CT	CC	CT	0	12	0	100			
PAPPA2	rs4492574	I	CT	TT	CT	11	9	55	45	11/9	Biallelic	Biallelic
											Paternal	Paternal
RHOBTB3	rs34896	I	AA	AG	AG	3	15	17	83	3/15	biased	biased

Mat, expression from the maternal allele; Pat, expression from the paternal allele.
