# Supplementary material for: Parent-of-origin-specific allelic expression in the human placenta is limited to established imprinted loci and it is stably maintained across pregnancy
Source: Clin Epigenetics. 2019 Jun 26;11:94. doi: 10.1186/s13148-019-0692-3 (PMC6595585; doi:10.1186/s13148-019-0692-3)
Supplement: Supplementary file 11 — Table S8. Genes that exhibit imprinting or biased parental allelic expression: literature evidence for the link to pregnancy, fetal disorders, or human disease. (PDF 107 kb) [file 13148_2019_692_MOESM11_ESM.pdf]

Table S8. Genes that exhibit imprinting or biased parental allelic expression: literature evidence for the link to pregnancy, fetal disorders or human disease.

Gene	Exp. allele	Expression[a]	Link to pregnancy, fetal or human disease	References
Placental imprinted genes				
MEG3 (ncRNA)	Mat	adrenal, placenta	IUGR, PE; vascular disease	[1, 2]
PHLA2	Mat	placenta	IUGR, PE, miscarriage, fetal death,	[1, 3–5]
RTL1	Mat	adrenal, placenta	IUGR, Fetal death	[6–8]
H19 (ncRNA)	Mat	placenta	IUGR, PE, BWS	[9–12]
PEG10	Pat	adrenal, placenta	IUGR, PE, placental defects, fetal death	[4, 13, 14]
IGF2	Pat	placenta	IUGR, miscarriage, fetal death, BWS, SRS	[1, 4, 9, 15]
MEST	Pat	placenta	GD, IUGR	[1, 16]
ZFAT	Pat	kidney, placenta	PE, autoimmune thyroid disease	[17, 18]
PLAGL1	Pat	placenta	IUGR, TNDM1	[19, 20]
DLK1	Pat	adrenal, placenta	GD, SGA	[21, 22]
AIM1	Pat	placenta	n.a.	n.a.
Genes with biased parental allelic expression in the human placenta				
KLHDC10	Mat	All tissues	n.a.	n.a.
NLRP2	Mat	Mixed tissues; high in testis	Maternal LoF cause BWS in children	[23]
GRB10	Mat	All tissues	Increased childhood glucose levels	[24]
NAA60	Mat	All tissues	n.a.	n.a.
CPXM2	Pat	Enhanced in epididymis, smooth muscle	IUGR, macrosomy; possibly implicated in multiple sclerosis	[25, 26]
MCCC1	Pat	All tissues	3-Methylcrotonylglycinuria	[27]
DCAF10	Pat	All tissues	Lung cancer	[28]
DNMT1	Pat	All tissues	Nervous system disorders, cancer	[29, 30]

RHOBTB3	Pat	All tissues	Possibly implicated in cancer	[31]
ZDBF2	Pat	Mixed tissues	IUGR, Nasopalpebral lipoma-coloboma syndrome	[32, 33]
MKRN3	Pat	Enhanced in cerebral cortex, placenta, testis	Familial idiopathic central precocious puberty	[34, 35]
GRHL1	Pat	Enhanced in esophagus, skin	Possibly implicated in cancer	[36]
NUDT12	Pat	Mixed tissues; high in parathyroid gland	Childhood asthma, chronic obstructive pulmonary disease	[37]
PLEKHG4B	Pat	Enhanced in thyroid, pituitary	n.a.	n.a.

[a] For the majority of genes, the information on the expression in human tissues/organs was derived from Proteinatlas [38]. For RNA genes, MEG3 and H19, the information on the tissue expression was derived from NCBI Gene [39].
BWS, Beckwith-Wiedemann syndrome; GD, gestational diabetes; IUGR, intrauterine growth restriction; LoF, loss-of-function; Mat, expression from the maternal allele; n.a., data not available; Pat, expression from the paternal allele; PE, preeclampsia; SGA, small-for-gestational-age newborn; SRS, Silver- Russell syndrome; TNDM1, transient neonatal diabetes mellitus 1; n.a., not available

References for Table S8.

1. McMinn J, Wei M, Schupf N, Cusmai J, Johnson EB, Smith AC, et al. Unbalanced Placental Expression of Imprinted Genes in Human Intrauterine Growth Restriction. Placenta. 2006;27:540–9. doi:10.1016/J.PLACENTA.2005.07.004.

2. Zhang Y, Zou Y, Wang W, Zuo Q, Jiang Z, Sun M, et al. Down-Regulated Long Non-Coding RNA MEG3 and its Effect on Promoting Apoptosis and Suppressing Migration of Trophoblast Cells. J Cell Biochem. 2015;116:542–50. doi:10.1002/jcb.25004.

3. Jin F, Qiao C, Luan N, Shang T. The expression of the imprinted gene pleckstrin homology-like domain family A member 2 in placental tissues of preeclampsia and its effects on the proliferation, migration and invasion of trophoblast cells JEG-3. Clin Exp Pharmacol Physiol. 2015;42:1142–51. doi:10.1111/1440-1681.12468.

4. Dória S, Sousa M, Fernandes S, Ramalho C, Brandão O, Matias A, et al. Gene expression pattern of *IGF2* , *PHLDA2* , *PEG10* and *CDKN1C* imprinted genes in spontaneous miscarriages or fetal deaths. Epigenetics. 2010;5:444–50. doi:10.4161/epi.5.5.12118.

5. De Crescenzo A, Sparago A, Cerrato F, Palumbo O, Carella M, Miceli M, et al. Paternal deletion of the 11p15.5 centromeric-imprinting control region is associated with alteration of imprinted gene expression and recurrent severe intrauterine growth restriction. J Med Genet. 2013;50:99–103. doi:10.1136/jmedgenet-2012-101352.

6. Fujioka K, Nishida K, Ashina M, Abe S, Fukushima S, Ikuta T, et al. DNA methylation of the Rtl1 promoter in the placentas with fetal growth restriction. Pediatr Neonatol. 2019. doi:10.1016/J.PEDNEO.2019.01.001.

7. Kitazawa M, Tamura M, Kaneko-Ishino T, Ishino F. Severe damage to the placental fetal capillary network causes mid- to late fetal lethality and reduction in placental size in *Peg11/Rtl1* KO mice.

Genes to Cells. 2017;22:174–88. doi:10.1111/gtc.12465.

8. Prats-Puig A, Carreras-Badosa G, Bassols J, Cavelier P, Magret A, Sabench C, et al. The placental imprinted DLK1-DIO3 domain: a new link to prenatal and postnatal growth in humans. Am J Obstet Gynecol. 2017;217:350.e1-350.e13. doi:10.1016/J.AJOG.2017.05.002.

9. Weksberg R, Shuman C, Beckwith JB. Beckwith–Wiedemann syndrome. Eur J Hum Genet. 2010;18:8–14. doi:10.1038/ejhg.2009.106.

10. Yu L, Chen M, Zhao D, Yi P, Lu L, Han J, et al. The H19 Gene Imprinting in Normal Pregnancy and Pre-eclampsia. Placenta. 2009;30:443–7. doi:10.1016/J.PLACENTA.2009.02.011.

11. Koukoura O, Sifakis S, Zaravinos A, Apostolidou S, Jones A, Hajiioannou J, et al. Hypomethylation along with increased H19 expression in placentas from pregnancies complicated with fetal growth restriction. Placenta. 2011;32:51–7. doi:10.1016/J.PLACENTA.2010.10.017.

12. Gao W, Liu M, Yang Y, Yang H, Liao Q, Bai Y, et al. The imprinted H19 gene regulates human placental trophoblast cell proliferation via encoding miR-675 that targets Nodal Modulator 1 (NOMO1). RNA Biol. 2012;9:1002–10. doi:10.4161/rna.20807.

13. Liang XY, Chen X, Jin YZ, Chen XO, Chen QZ. Expression and significance of the imprinted gene PEG10 in placenta of patients with preeclampsia. Genet Mol Res. 2014;13:10607–14. doi:10.4238/2014.December.18.2.

14. Rahat B, Mahajan A, Bagga R, Hamid A, Kaur J. Epigenetic modifications at DMRs of placental genes are subjected to variations in normal gestation, pathological conditions and folate supplementation. Sci Rep. 2017;7:40774. doi:10.1038/srep40774.

15. Gicquel C, Rossignol S, Cabrol S, Houang M, Steunou V, Barbu V, et al. Epimutation of the telomeric imprinting center region on chromosome 11p15 in Silver-Russell syndrome. Nat Genet. 2005;37:1003–7. doi:10.1038/ng1629.

16. Hajj N El, Pliushch G, Schneider E, Dittrich M, Müller T, Korenkov M, et al. Metabolic Programming of MEST DNA Methylation by Intrauterine Exposure to Gestational Diabetes Mellitus. Diabetes. 2013;62:1320–8. doi:10.2337/DB12-0289.

17. Barbaux S, Gascoin-Lachambre G, Buffat C, Monnier P, Mondon F, Tonanny M-B, et al. A genome-wide approach reveals novel imprinted genes expressed in the human placenta. Epigenetics. 2012;7:1079–90. doi:10.4161/epi.21495.

18. Shirasawa S, Harada H, Furugaki K, Akamizu T, Ishikawa N, Ito K, et al. SNPs in the promoter of a B cell-specific antisense transcript, SAS-ZFAT, determine susceptibility to autoimmune thyroid disease. Hum Mol Genet. 2004;13:2221–31. doi:10.1093/hmg/ddh245.

19. Mackay DJG, Temple IK. Transient neonatal diabetes mellitus type 1. Am J Med Genet Part C Semin Med Genet. 2010;154C:335–42. doi:10.1002/ajmg.c.30272.

20. Iglesias-Platas I, Martin-Trujillo A, Petazzi P, Guillaumet-Adkins A, Esteller M, Monk D. Altered expression of the imprinted transcription factor PLAGL1 deregulates a network of genes in the human IUGR placenta. Hum Mol Genet. 2014;23:6275–85. doi:10.1093/hmg/ddu347.

21. Li J, Zhu Q, Wang H, Han C, Zhou Q, Huang H, et al. Decreased fetal pre-adipocyte factor-1 in pregnancies complicated by gestational diabetes mellitus. Clin Chim Acta. 2014;431:93–5. doi:10.1016/J.CCA.2014.01.048.

22. Díaz M, Bassols J, Aragonés G, Mazarico E, López-Bermejo A, Ibáñez L. Decreased placental expression of pre-adipocyte factor-1 in children born small-for-gestational-age: Association to early postnatal weight gain. Placenta. 2013;34:331–4. doi:10.1016/J.PLACENTA.2013.01.011.

23. Meyer E, Lim D, Pasha S, Tee LJ, Rahman F, Yates JRW, et al. Germline Mutation in NLRP2 (NALP2) in a Familial Imprinting Disorder (Beckwith-Wiedemann Syndrome). PLoS Genet. 2009;5:e1000423. doi:10.1371/journal.pgen.1000423.

24. Sohani ZN, Anand SS, Robiou-du-Pont S, Morrison KM, McDonald SD, Atkinson SA, et al. Risk Alleles in/near ADCY5, ADRA2A, CDKAL1, CDKN2A/B, GRB10, and TCF7L2 Elevate Plasma Glucose Levels at Birth and in Early Childhood: Results from the FAMILY Study. PLoS One. 2016;11:e0152107. doi:10.1371/journal.pone.0152107.

25. Sabri A, Lai D, D'Silva A, Seeho S, Kaur J, Ng C, et al. Differential Placental Gene Expression in Term Pregnancies Affected by Fetal Growth Restriction and Macrosomia. Fetal Diagn Ther. 2014;36:173–80. doi:10.1159/000360535.

26. Gil-Varea E, Urcelay E, Vilariño-Güell C, Costa C, Midaglia L, Matesanz F, et al. Exome sequencing study in patients with multiple sclerosis reveals variants associated with disease course. J Neuroinflammation. 2018;15:265. doi:10.1186/s12974-018-1307-1.

27. Baumgartner MR, Almashanu S, Suormala T, Obie C, Cole RN, Packman S, et al. The molecular basis of human 3-methylcrotonyl-CoA carboxylase deficiency. J Clin Invest. 2001;107:495–504. doi:10.1172/JCI11948.

28. Yan H, Bi L, Wang Y, Zhang X, Hou Z, Wang Q, et al. Integrative analysis of multi-omics data reveals distinct impacts of DDB1-CUL4 associated factors in human lung adenocarcinomas. Sci Rep. 2017;7:333. doi:10.1038/s41598-017-00512-1.

29. Baets J, Duan X, Wu Y, Smith G, Seeley WW, Mademan I, et al. Defects of mutant DNMT1 are linked to a spectrum of neurological disorders. Brain. 2015;138:845–61. doi:10.1093/brain/awv010.

30. Ramassone A, Pagotto S, Veronese A, Visone R, Ramassone A, Pagotto S, et al. Epigenetics and MicroRNAs in Cancer. Int J Mol Sci. 2018;19:459. doi:10.3390/ijms19020459.

31. Long M, Simpson JC. Rho GTPases operating at the Golgi complex: Implications for membrane traffic and cancer biology. Tissue Cell. 2017;49:163–9. doi:10.1016/J.TICE.2016.09.007.

32. Monteagudo-Sánchez A, Sánchez-Delgado M, Mora JRH, Santamaría NT, Gratacós E, Esteller M, et al. Differences in expression rather than methylation at placenta-specific imprinted loci is associated with intrauterine growth restriction. Clin Epigenetics. 2019;11:35. doi:10.1186/s13148-019-0630-4.

33. Chacón-Camacho OF, Sobreira N, You J, Piña-Aguilar RE, Villegas-Ruiz V, Zenteno JC. Exome sequencing identifies a de novo frameshift mutation in the imprinted gene *ZDBF2* in a sporadic patient with Nasopalpebral Lipoma-coloboma syndrome. Am J Med Genet Part A. 2016;170:1934–7. doi:10.1002/ajmg.a.37683.

34. Simon D, Ba I, Mekhail N, Ecosse E, Paulsen A, Zenaty D, et al. Mutations in the maternally imprinted gene MKRN3 are common in familial central precocious puberty. Eur J Endocrinol. 2016;174:1–8. doi:10.1530/EJE-15-0488.

35. de Vries L, Gat-Yablonski G, Dror N, Singer A, Phillip M. A novel MKRN3 missense mutation causing familial precocious puberty. Hum Reprod. 2014;29:2838–43. doi:10.1093/humrep/deu256.

36. Frisch SM, Farris JC, Pifer PM. Roles of Grainyhead-like transcription factors in cancer. Oncogene. 2017;36:6067–73. doi:10.1038/onc.2017.178.

37. Dekker HT den, Burrows K, Felix JF, Salas LA, Nedeljkovic I, Yao J, et al. Newborn DNA-methylation, childhood lung function, and the risks of asthma and COPD across the life course. Eur Respir J. 2019;:1801795. doi:10.1183/13993003.01795-2018.

38. Uhlén M, Fagerberg L, Hallström BM, Lindskog C, Oksvold P, Mardinoglu A, et al. Tissue-based

map of the human proteome. Science (80-). 2015;347:1260419–1260419. doi:10.1126/science.1260419.

39. NCBI Gene. https://www.ncbi.nlm.nih.gov/gene.
